# Arabidopsis phenotyping through geometric morphometrics

**DOI:** 10.1093/gigascience/giy073

**Published:** 2018-06-18

**Authors:** Carlos A Manacorda, Sebastian Asurmendi

**Affiliations:** 1 Instituto de Biotecnología, CICVyA, INTA, Nicolas Repetto y de los Reseros s/n, Hurlingham, (1686) Buenos Aires, Argentina; 2 CONICET, Nicolas Repetto y de los Reseros s/n, Hurlingham, (1686) Buenos Aires, Argentina

**Keywords:** Arabidopsis, Geometric Morphometrics, Procrustes Analysis, landmarks, phenotyping, ORMV, TuMV

## Abstract

**Background:**

Recently, great technical progress has been achieved in the field of plant phenotyping. High-throughput platforms and the development of improved algorithms for rosette image segmentation make it possible to extract shape and size parameters for genetic, physiological, and environmental studies on a large scale. The development of low-cost phenotyping platforms and freeware resources make it possible to widely expand phenotypic analysis tools for Arabidopsis. However, objective descriptors of shape parameters that could be used independently of the platform and segmentation software used are still lacking, and shape descriptions still rely on *ad hoc* or even contradictory descriptors, which could make comparisons difficult and perhaps inaccurate. Modern geometric morphometrics is a family of methods in quantitative biology proposed to be the main source of data and analytical tools in the emerging field of phenomics studies. Based on the location of landmarks (corresponding points) over imaged specimens and by combining geometry, multivariate analysis, and powerful statistical techniques, these tools offer the possibility to reproducibly and accurately account for shape variations among groups and measure them in shape distance units.

**Results:**

Here, a particular scheme of landmark placement on Arabidopsis rosette images is proposed to study shape variation in viral infection processes. Shape differences between controls and infected plants are quantified throughout the infectious process and visualized. Quantitative comparisons between two unrelated ssRNA+ viruses are shown, and reproducibility issues are assessed.

**Conclusions:**

Combined with the newest automated platforms and plant segmentation procedures, geometric morphometric tools could boost phenotypic features extraction and processing in an objective, reproducible manner.

## Background

Plant phenotyping, the process of recording quantitative and qualitative plant traits, is essential to the study of plant responses to the environment [1]. A survey of the International Plant Phenotyping Network [2] plant scientists found that most participants think that plant phenotyping will play an important role in the future. The selected topics of interest were the assessment of abiotic, biotic, and multiple stress and the model plant *Arabidopsis thaliana*. Recently, many new techniques have been developed to facilitate and improve quantitative plant phenomics (i.e., the full set of phenotypic features of an individual), going from destructive to nondestructive and even high-throughput phenotyping (the use of cameras and automated platforms to automatically extract phenotypic features on hundreds of plants per day) [3–5]. Whereas the throughput is an important aspect of phenotyping, spatial and temporal resolutions, as well as accuracy, should be considered [6].

Freely available software that overcomes the difficult task of image rosette segmentation (i. e., the computational separation of the living plant tissue from the substrate background) is still under investigation and has been developed by different means [7–11]. These software packages allow the assessment of several rosette parameters such as area, perimeter, and other more complex descriptors. However, the persistence of *ad hoc* descriptors [12, 13] and lack of a gold standard could give rise to reproducibility issues, because of different growing substrate-segmentation algorithm combinations. Moreover, different approaches sometimes give the same name to different parameters (e.g., “roundness” in ImageJ, [14] vs. [10]) or different names to the same parameter (e.g., “solidity” in [11] equals “compactness” in [7, 10] and “surface coverage” in [5]). The need to develop objective, mathematically, and statistically sound and more accurate shape descriptors in plants has been stressed in recent reviews on the topic [15–17]. Nonetheless, image dataset analyses require a conceptual and statistical corpus of knowledge that is not always present in a plant biologist's research field. Plant phenotyping relies on skills and technologies that are used to characterize qualitative or quantitative traits regardless of the throughput of the analyses [1]. One such knowledge corpus is morphometrics [18].

Traditional morphometric analyses, such as measures and ratios of length, depth, and width, were widely used in evolutionary biology, taxonomy, and similar studies throughout the 20th century. At the end of that century, the seminal work of Thompson [19] was reevaluated in light of multivariate analysis and novel mathematical developments [20, 21], giving rise to modern geometric morphometrics (GM), which was called a “revolution” in morphometrics [22–24].

GM combines geometry, multivariate morphometrics, computer science, and imaging techniques for a powerful and accurate study of organismal forms. This family of methods in quantitative biology is proposed to be the main source of data and analytical tools in the emerging field of phenomics [25]. Formally, GM is “a collection of approaches for the multivariate statistical analysis of Cartesian coordinate data, usually (but not always) limited to landmark point locations” [26]. Landmark methods have been successfully applied to various species and have the advantage of being easy to understand [27]. In addition to enhanced statistical power and better descriptive and graphical tools, GM allows researchers to decompose form in size and shape. The whole configuration of the organism under study is analyzed, rather than relying on the description of relative displacements of pairs of points. GM is now a mature discipline that has been widely applied in biology [28–30] (see [31] for a review). For example, barley seeds [32] and grapevine leaves [33] and oak leaves [34, 35] were studied using GM methods.

Plant viruses cause important worldwide economic losses in crops [36]. Symptoms include plant stunting, changes in leaf morphology, and, in some cases, plant death [37]. These symptoms vary depending on various aspects, including genetic compatibility and environmental conditions. Given a particular host-virus interaction, different viral strains trigger different symptomatology, which is more or less subtle for the observer to distinguish [38–40]. Comparing the severity of qualitative viral symptoms (i.e., the degree to which infected plants depart from healthy controls in some observable phenotype, often referred to as the aerial parts of the plant such as leaves, stems, or rosettes) is a difficult task; it is performed mainly by visually rating symptoms (e.g., [41]). Consequently, morphological differences could be difficult to describe and reproducibility issues could arise.

*Arabidopsis thaliana* has been extensively used in studies on the influences of environmental factors on plants, paving the way to the development and testing of experimental techniques and data analysis methods [42]. The Arabidopsis rosette is a nearly two-dimensional (2D) structure in the vegetative phase [11], thereby facilitating image acquisition and interpretation. Here, a case study is proposed where GM tools are applied to study and quantitatively describe morphometric changes triggered in *A. thaliana* plants by RNA viruses belonging to two unrelated families. It is proposed that a particular selection of landmarks be located in the Arabidopsis rosette during its vegetative phase. The study spans from the early stages of viral infection to later periods when symptoms are detectable with the naked eye. Comparisons are made between the discriminant power of computer-assisted classification and the expert human eye. Symptom severity triggered by both viruses is also compared based on the relative morphometric changes induced relative to healthy controls. Changes in allometric growth, phenotypic trajectories, and morphospace occupation patterns are also investigated. Size analyses are also performed. Throughout this work, several bioinformatics resources were applied in order to both extract the higher degree of information available and to exemplify different and complementary possibilities that GM offers for the accurate description of shape in Arabidopsis.

In this work, we aim to introduce the use of GM tools for analysis of the Arabidopsis rosette. Its purpose is to statistically quantify the shape differences between treatments in order to establish objective global comparisons on a matter that is usually subjective, virus phenotype severity. Viral infections are used as case studies to exemplify the potential usefulness of these techniques to quantitatively reveal shape changes using this plant model. As such, it is not intended to offer a complete introductory explanation of each GM tool, an objective that is beyond the scope of this article. Such a task has been performed by [35]. For a complete introductory explanation of GM tools applied in biological systems, refer to [43]. Software used in this work frequently has its own user’s manual and informative examples [44–46]. Nevertheless, for the purpose of facilitating the comprehension of this work to newcomers in the field of GM, each tool is briefly described in the Materials and Methods section.

## Materials and Methods

### Plant growth conditions

*Arabidopsis thaliana* Col-0 seeds were stratified at 4°C for 3 days. Plants were grown under short-day conditions (10 hours light/14 hours dark cycle, T(°C) = 23/21, Hr(%) = 60/65, and a light intensity of 150 μE m-2 s-1) in a controlled environmental chamber (Conviron PGR14; Conviron, Winnipeg, Manitoba, Canada). Plants were grown in individual pots in trays, and treatments were assigned to plants in all trays. One experiment was performed with oilseed rape mosaic virus (ORMV) and two independent experiments were carried on with turnip mosaic virus-UK1 strain (TuMV-UK1).

### Virus infection assays

ORMV [47] was maintained in *Nicotiana tabacum* (cv. Xhanti nn), and infective sap was obtained after grinding infected leaves with mortar and pestle in 50 mM phosphate buffer, pH = 7.5. TuMV-UK1 strain (accession number X65978) [48] was maintained in infected *A. thaliana* Col-0. Fresh sap was obtained immediately prior to use in order to inoculate plants with sodium sulfite buffer (1% K_2_HPO_4_ + 0.1% Na_2_SO_3_ [wt/vol]). Mock-inoculated plants were rubbed with carborundum dust with either 50 mM phosphate buffer, pH = 7.5, or sodium sulfite buffer, respectively. Plants were mechanically inoculated in their third true leaf at stage 1.08 at 21 days post-sowing [49]. This was done because the leaves were almost fully developed by the time of the procedure and therefore constituted a source tissue for the export of virions to the rest of the plant. The number of plants assigned to each treatment in each experiment was: Mock = 23 and ORMV = 17, Mock = 27 and TuMV = 14 (TuMV 1st experiment) and Mock = 14 and TuMV = 8(TuMV 2nd experiment)

### Image acquisition

Zenithal photographs of individual plants growing in pots were taken with a Canon PowerShot SX50HS camera mounted on a monopod at maximum resolution. Specimens were imaged at different days post-inoculation (DPI), spanning 3 to 12 DPI. Photographs were taken at the same time of day on successive days to minimize error. A ruler was placed next to each plant at each image acquisition, and only the plant's central part (60–80 mm) was taken into account to avoid image distortion at the edges of the photograph [50].

### Landmark configuration and digitization

At the heart of GM analyses is the concept of landmarks. Landmarks are points that can be located precisely over a structure and correspond in a one-to-one manner among all the specimens included in a study [51]. There is no absolute landmark configuration on any given structure. The choice of the number of landmarks and their configuration depend on the hypothesis being tested [52]. Here, the focus was on the phenotypic impact of viral infections on the Arabidopsis rosette over time. Hence, short-day conditions were chosen to maintain the vegetative phase and delay flowering (when stems and reproductive organs could mask morphological features of leaves in zenithal photographs), allowing the plant’s aerial part to remain near 2D during the experiment. Landmarks were chosen based, in part, on the relative ease of recognition in an Arabidopsis rosette in the vegetative phase. Landmark choice was also made to encompass, as broadly as possible, the phenotypic changes experienced by the plant during the infection. Chosen landmarks were present from early stages of infection to later stages and placed in regions that experience dramatic changes when infected [39]. Also, selected landmarks could take into account relevant morphological changes induced by stresses or distinctive phenotypes of different ecotypes such as relative shortening or lengthening of petioles and laminae or relative lateral displacement of leaves [7, 10, 11]. Moreover, the landmarks chosen are probably less prone to manual digitization error than, e.g., a landmark situated in the middle of the laminae or placed somewhere along the leaf’s contour. This is a task that seems rather complicated given the serrated nature of Arabidopsis leaves and the fact that the degree and placement of serration changes along the development of successive leaves [53–55]. A relatively reduced number of landmarks can be used to describe complex forms [35, 56].

Also, the selection of landmarks is based on the observation of the following five basic principles, including the basic requirements for 2D approximation [43]:

1. Homology (in the sense of correspondence of points). The points on one specimen correspond (as the “same” point) to that point on all individuals.
2. Adequate coverage of the form (or comprehensive coverage). Landmarks should be chosen so that they encompass the structure over which the biological hypothesis of interest is being tested and are functional to the specific aim of a study.
3. Repeatability. The same landmarks should be easily identified in the same structure in order to reduce digitization error (a component of measurement error).
4. Consistency of relative position. This attribute guarantees that landmarks do not interchange relative positions.
5. Coplanarity. When digitizing real, three-dimensional (3D) structures under the 2D approximation, landmarks should be placed as close as possible onto an imaginary plane to reduce the distortion associated with that approximation.

**Figure 1:**
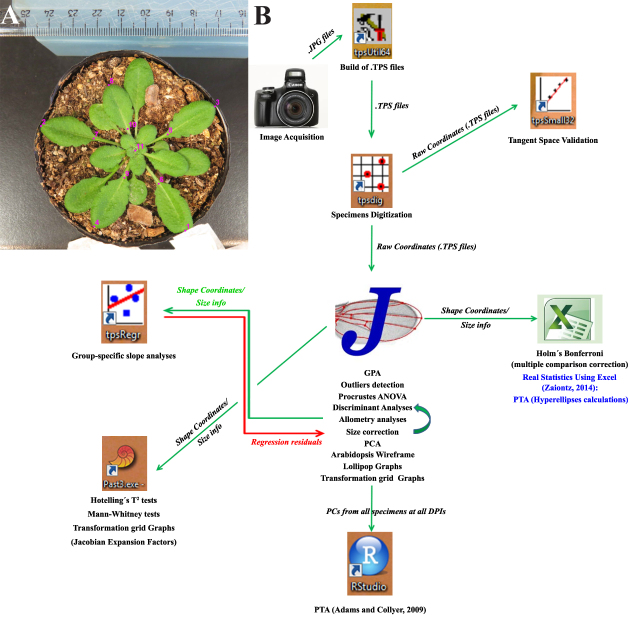
**(A)** Landmark configuration in a representative Arabidopsis rosette. An 8-DPI mock-inoculated rosette is shown. **(B)** Analysis flowchart showing the different software used in this study, with the main features extracted from each one that is listed below the corresponding icon. See the Main Text and Materials and Methods for details.

The TPSUtil software (a member of the TPS series of GM tools [46] that prepares the data for further analyses) was used to create .TXT files with a .TPS extension from the directories containing the .JPG images. These were used to load the .JPG images in TPSDig2. Opening these .TPS files with TPSDig2 allows the user to proceed with the digitization of landmarks. An 11-landmark configuration for the Arabidopsis rosette is shown in Fig. 1A. The 11 landmarks were digitized in the same order on each picture, after setting a scale factor with a ruler, at each DPI. This scale factor is set in TPSDig2 selecting two points placed at a known distance between them in the photograph and allows for the correction of possible differences in distances from the camera objective to the specimen under study (from one day to the next, for instance). The scale factor is important to measure centroid size, among other possible measures, but has no effect on landmark coordinates, which remain in pixel units [46].

Following Bookstein's criteria [57], landmark 11 (which is situated at the center of the rosette) is a type 1 landmark because it is the intersection of the base of all petioles, i.e., its juxtaposition and, hence, is very locally defined. Type 1 landmarks are frequently considered as optimal [43, 57]. Landmarks 1–5 (which are located at the tip of leaves 8–12 and are the maximum of curvature of that structure) and landmarks 6–10 (which are located at the intersection of the petiole and the lamina of each leaf from 8–12) cannot be unambiguously assigned due to the continuous nature of the leaf curvature and are type 2 landmarks. Leaves below leaf 8 were not chosen for landmark placement for three main reasons: they are hidden for younger leaves at later stages of infection; these old leaves had almost finished their growth by the time the first photographs were taken (and the form covered by them would be a less informative one for the process of shape and size change upon viral infection); and the senescence process of older leaves leads to morphological changes derived from dehydration and death. Younger leaves (beyond leaf 12) were not chosen because they were not present at the earlier stages of infections, therefore, violating the requisite of repeatability of landmarks.

Average specimen digitization time was about 1 minute. The output of TPSDig2 is a .TPS file containing information about specimen name, scale factor, and raw coordinates of each landmark for all digitized specimens. Landmark digitization was repeated 1 week later in order to estimate the digitization component of the measurement error for each specimen.

### Workflow

A flowchart of data analyses is shown in Fig. 1B. Image datasets for all DPIs and both treatments were handled and digitized for further analyses using the TPSUtil and TPSDig2 software packages that generate .TPS output files. Several freeware packages can be used to extract shape information from .TPS files [43]. Here, MorphoJ software [44] was chosen mainly because of its ease of use and comprehensive tools available. MorphoJ creates new datasets from several file extensions including .TPS. The “Supplementary File ORMV.morphoj” was created and 16 datasets were generated, one for each DPI and digitization instance. Specimens were classified according to ID, treatment, DPI, and digitization for each dataset. Combinations of classifiers were also made in order to perform further grouped analyses. Other complimentary analyses and shape change visualizations were performed by exporting output files from MorphoJ to other software packages. TuMV analyses were done in the same way.

### Procrustes fit and outlier detection

The first step of shape analysis in GM consists of extracting shape coordinates from raw data obtained at the digitization step. The standard procedure in GM studies is the generalized Procrustes analysis (GPA).

Procrustes procedures are performed in order to remove from the specimens all information that is not relevant for shape comparisons, including size. Specimens are first translated at the origin (“superimposed”) by subtracting the coordinates of its centroid from the corresponding (X or Y) coordinates of each landmark. Then, differences in size are removed by rescaling each specimen to the mean centroid size (CS), which is calculated as the square root of the summed squared distances of each landmark from the centroid. Differences in rotation are eliminated by rotating specimens, thus, minimizing the summed squared distances between homologous landmarks (over all landmarks) between the shapes. MorphoJ performs a full Procrustes fit that is a variant of the analysis that is more conservative and resistant to outliers of shape.

In a few recent studies that focused on flowers [58–60] or leaves [61], asymmetry in plants was examined by comparing GM with those in animals [51]. Although we did not study the asymmetry issue, it must be briefly considered. In Arabidopsis, the arrangement of organs along the stem (phyllotaxy) follows a predictable pattern, the Fibonacci series. Phyllotaxy orientation can be clockwise or counterclockwise [62]. There is no preferred orientation of Arabidopsis rosettes; in this study, 20 were clockwise and 21 were counterclockwise. This is an example of antisymmetry, where (following Klingenberg’s [51] explanation) “most individuals are asymmetric, but differ in the directions of the asymmetries so that there is a mix of 'left-sided' and 'right-sided' individuals.” This creates a bimodal distribution that should be considered because clockwise and counterclockwise rosettes are biological enantiomorphs and must not be directly superimposed by GPA. Fortunately, MorphoJ automatically performs reflections on every specimen when executing a GPA; therefore, it is not a problem at this stage. However, care must be taken with different software. Alternatively, rosettes can be reflected using TPSDig2 to leave all clockwise or counterclockwise rosettes prior to landmark digitization.

Although the full Procrustes fit performed by MorphoJ is considered to be more resistant to outliers of shape [44], there could still be specimens that divert from the rest to a great extent. The “Find Outliers” option in the “Preliminaries” menu provides an indication of how unusual an individual is relative to the others in the sample (using Mahalanobis distance in larger samples). The user can, therefore, consider subtracting this specimen from the rest as an outlier. A GPA was run for each dataset (each comprising one of the DPIs and one digitization replicate), and outliers were evaluated separately in each of the 16 datasets.

### Assessment of the tangent space approximation

For a given M-dimensional structure with K landmarks (here, M = 2 and K = 11), an individual’s shape can be visualized as a point in an M x K multidimensional space (a hypersphere). After centering and rescaling, three dimensions are lost and shapes are said to be in a preshape space; they are not rotated yet. The distance in the hypersphere surface at which rotation differences between shapes are minimal is called the Procrustes distance (the conventional measure of a morphometric distance in GM [63]). Afterward, a reference (average) shape is selected and all other shapes are rotated to minimize distances relative to it, generating a shape space and losing one more dimension (remaining 2K-4). Because distances over curved multidimensional spaces are non-Euclidean, conventional tools of statistical inference cannot be used. Fortunately, a good approximation to Euclidean distances for most biological shapes is possible by projecting shape points to a tangent Euclidean space (for a visual explanation, see [43]). This assumption should, however, be tested when analyzing new data. TPSSmall is used to determine whether the amount of variation in shape in a dataset is small enough to perform statistical analyses in the linear tangent space approximate to Kendall's shape space, which is nonlinear. Basically, this task is performed by comparing the Procrustes distances obtained using both shape spaces. Since TPSSmall does not perform reflections, datasets analyzed with TPSDig2 were opened again and specimens reflected when necessary to leave all clockwise rosettes. Two data subsets were created for each DPI, one with mock-inoculated plants and the other with ORMV-infected plants. Next, the datasets were combined across DPIs using the “Append files” option of TPSUtil to create three main datasets—mock, ORMV, and all plants.

### Testing digitization error and variation between treatments using Procrustes ANOVA

As mentioned previously, two digitization instances were performed on each plant at each DPI in order to evaluate digitization error. This procedure is important because digitization error should always account for far less variance in the subsequent analyses than specimens and treatments do [35]. The differences between the samples and particularly between the treatments are the ones worth investigating, not the human error in landmark placement. Purposely, datasets for each DPI were combined and subjected to a hierarchical analysis of variance (ANOVA). In MorphoJ this is a Procrustes ANOVA, with “Treatment” as an additional main effect, “ID” for the individuals, and “Digitization” as the Error1 source (the last term is equivalent to the Residuals here, as only one source of error is being quantified). One Procrustes ANOVA was performed separately for each DPI.

In Procrustes ANOVA, variance is partitioned by means of hierarchical sum of squares (SS), which implies that each effect is adjusted for effects that appear earlier in the hierarchy. This takes into account the nested structure of the data and, therefore, allows the quantification of differences in Treatments and individuals (plants) regardless of Treatment. (This ssue is crucial if the design is unbalanced, i.e., with unequal sample sizes, in part, because hypothesis tests are more robust to the assumptions of normality and equal variance when the design is balanced. Although the design here is unbalanced (24 mock and 17 ORMV plants), it is considered a minor problem for designs that are not extremely unbalanced and/or do not involve more than one factor [43, 64]. It should, however, be taken into account when multiple factors are studied, requiring special calculations for obtaining the correct SS. MorphoJ also recommends use of data that are as balanced as possible (see [43] for a discussion on the topic). The variance unexplained by any of these effects (Treatment and Individual) is digitization error, and it is estimated using the differences between digitizations. Hence, total variance was decomposed into main (Treatment) and random (ID and Digitization) components and was expressed as a percentage of total variance for each DPI. Statistical significance is provided by Goodall *F* tests [65] for size and shape. The parametric Goodall *F* test assumes isotropic variation (the assumption that there is an equal amount of variation around each landmark), which is often violated in biological studies [66]. For many practical applications, it is possible to use the approach based on Procrustes distances to assess the relative magnitudes of effects. Hoever, when making statistical inferences, the multivariate analysis of variance (MANOVA) approach is used [44]. For this reason, MorphoJ includes a multivariate test (Pillai trace) for shape.

### Ordination methods and shape change visualization

#### PCA (Principal Component Analysis)

Once shape variables (the 22 Procrustes coordinates) are extracted for all specimens at each DPI, it is a useful option to plot differences between individuals and treatments. However, patterns of variation and covariation between lots of variables are difficult to interpret, and shape variables are not statistically independent [43]. PCA (Principal Component Analysis) is a technique that simplifies those patterns and, therefore, makes them easier to interpret. When a PCA is performed, the original, possible correlated set of shape variables are mathematically transformed to create a new set of orthogonal and independent variables (known as principal components [PCs]) that are a linear combination of the original variables. PCs do not covary but carry all the original shape information. As each PC explains sequentially less shape variance, this approach is often used to restrict the analysis to the first few PCs that account for most of the total variance [43]. However, some recent developments in the GM field [67] propose that PCA should be, at the least, carefully interpreted since the biological meaning of the PC axes are difficult to assess. Here, PCA is used conservatively to discuss relative shape distances between individuals belonging to different groups, as advised by Howells [68]. It is also important to remember that PCA is useful for studying distances between individuals, not groups, and that although it is a powerful descriptive tool, it does not involve any statistical test. Therefore, the relative separation of groups in a PCA plot does not allow one to draw conclusions about significant differences (or its absence).

#### Visualization of shape changes

A brief description of common GM visualization tools is needed in order to accurately interpret the results. After the GPA, every configuration in the sample is optimally aligned to the average configuration and nearly optimally aligned to every other configuration in the sample [69]. GPA has already removed differences attributable to size, position, and orientation from configurations. All differences that remain are shape variation. Accordingly, shape differences are found using the relative displacements of the landmarks from one shape to another nearby in shape space [69].

A key concept to bear in mind is that it is fundamentally wrong to consider landmarks as displacements in an isolated manner [43, 69] (see example in [35]). This is because all the landmarks in the GPA jointly determine the alignment of each configuration in relation to the mean shape. Then, the variation in the position of each landmark after superimposition is relative to the positions of all other landmarks. Although a shift is shown at every landmark, these shifts are relative to all other landmarks. Lollipop and wireframe graphs are based on these assumptions (see the Results section).

Shape variation could be depicted by means of transformation grids, which are mathematically constructed following the thin-plate spline technique [43, 57, 69]. Briefly, landmarks of a starting shape are placed on a grid of an imaginary infinitely thin metal plate. Landmarks of a target configuration are placed on another grid with equal characteristics, and both metal sheets are superimposed. Each landmark in the starting shape (e.g., mean shape) is linked to its homologous in order to reach the target configuration, and the deformation caused in the spline is calculated, finding the smoothest interpolating function that estimates energy changes in the spline between landmarks. Importantly, unlike lollipop or wireframe graphs, transformation grids distribute the change in landmark positions to the space between landmarks, when no objective information is available. Then, whereas a powerful descriptive tool, transformation grids must be carefully interpreted, especially regarding regions of the object that do not have landmarks nearly positioned [43, 69]. More details and examples are given in the Results section.

#### Discriminant analysis

Discriminant analysis (DA) is mathematically related to PCA. It finds the axes that optimize between-group differences relative to within-group variation. It can be used as a classification tool [43]. Here, it is used for testing treatments by using tests for sample mean differences, including an estimate of the accuracy of shape in predicting groups. The capability of DA to correctly assign specimens to treatments was assessed along with the experiment using the averaged datasets for each DPI. In MorphoJ, DA analysis was requested, selecting “Treatment” as the classification criterion. By default, DA in MorphoJ performs a parametric Hotelling T-square test (multivariate equivalent of the Student *t*test). Here, requested permutation tests were also performed for the Procrustes and Mahalanobis distances with 1,000 random runs. Procrustes and Mahalanobis distances show how far shapes from one group are from the mean of the other group.

### Allometric patterns and size correction

The covariation between a size variable and shape variables is called allometry. Isometry, by contrast, is the condition where size and shape are independent of each other and usually serves as the null hypothesis. These concepts are rooted in the Gould-Mosimann school of allometry that conceptually separates size and shape [70]. Although size had been removed from forms after GPA, thus leaving shape differences free of it, a consistent trend in change of shape with size may be possible. Allometry can be statistically tested by tests of multivariate regression.

When groups are present, a single regression line through all groups cannot be fit to test allometry because lines could have group-specific slopes or intercepts [35]. To test whether an allometric component is present in each group, separate regressions were performed for each treatment and DPI, with Procrustes coordinates and ln(CS) as dependent and independent variables, respectively. Permutation tests were requested with 10,000 runs.

When at least one group has regression slopes that are different from zero, several tests could be done in order to control for size and repeat analyses in order to assess whether differences in shape are actually the result of size variation only [34, 35, 43, 70]. A multivariate analysis of covariance (MANCOVA) (with treatments as groups, shape coordinates as dependent variables, and ln(CS) as the independent variable) was run from 3 to 8 DPI. The MANCOVA is first run to allow each group to have its own slope. Next, the regression analysis is run again, but this time it fits a MANCOVA with the slopes constrained to be the same in each group (i.e., parallel). Although the percentage of variance explained (% SS) for the regression of the first model is always higher than the second one (constrained by the premise to keep parallel the slopes), the allometric trajectories could be considered to be parallel if differences are small. TPSRegr (v.1.41) provides multivariate and permutation tests for the assessment of that difference [46]. Afterward, the MANCOVA model tests if slopes are parallel but separate or if they are coincident (same Y-intercept), and a common regression slope including individuals from both treatments could be fit. This allows correction for size and testing, e.g., if DAs are improved after removing the “size-effect” [35].

### Phenotypic trajectory analyses and morphospace occupation patterns

Whereas the comparison of allometric vectors allows testing of whether shape change is altered at definite DPIs during ORMV infection, a more complete view of ontogenetic alterations needs to measure phenotypic evolution across multiple levels. It allows ontogenetic patterns to be characterized as phenotypic trajectories through the morphospace, rather than phenotypic vectors. The method proposed by Adams and Collyer [71] “may also be used for determining how allometric or ontogenetic growth trajectories differ, or for quantifying patterns in other data that form a time-sequence” [71]. Briefly, phenotypic trajectories have three attributes: size, direction, and shape.

Trajectory size (*MD*) quantifies the path length of the phenotypic trajectory expressed by a particular group across levels. This represents the magnitude of phenotypic change displayed by that group. If trajectories of two or more groups compared over comparable time periods differ in trajectory size, then it indicates differences in rates of morphological change.

Trajectory direction (*θ*) is a multivariate angle that describes the general orientation of phenotypic evolution in the multivariate trait space. Statistical comparisons of trajectory direction can be used to provide an assessment of patterns of convergence, divergence, and parallelism.

Trajectory shape (*D_Shape_*) describes the shape of the path of phenotypic evolution through the multivariate trait space. This information is useful because it indicates whether there are differences in how each group occupies the morphospace through the time period.

Phenotypic trajectory analyses (PTA) starts from the PCs for all specimens at all DPIs. They were obtained from the “Combined dataset 3–12 DPI, averaged by ID DPI” of the Supplementary File ORMV.morphoj. The R script developed by Adams and Collyer [71] was run in RStudio [72, 73].

However, as has been pointed out by Ciampaglio et al. [74], no one method of disparity measurement is sufficient for all purposes. The use of a combination of techniques should allow a clearer picture of disparity to emerge. With this aim, another available approach to compare shape trajectories through multivariate morphospace was used. Originally developed to study unequal morphological diversification in a clade of South American fishes [75], this approach is useful because it allows us to investigate whether a group “explores” a different amount of morphospace than others, in addition to possible differences in magnitude of phenotypic evolution. Moreover, density parameters could be calculated to determine whether the amount of morphological change is more or less constrained in the morphospace.

The method was adapted to the present study. As there is not a phylomorphospace and both treatments lack a “common ancestor,” but each plant follows its own independent ontogenetic path, nodes and branches do not exist. Rather, each plant possesses its own trajectory without points in common. Therefore, morphological trajectories were calculated for all plants taking these considerations into account. For this purpose, the “Combined dataset 3–12 DPI, averaged by ID DPI” of the Supplementary File ORMV.morphoj was subdivided by ID. Forty new datasets (mock- and ORMV-inoculated plants from the same previously performed Procrustes fit) were obtained and Procrustes coordinates and eigenvalues from the seven PCs obtained were exported to an Excel spreadsheet.

### Statistical analyses

Except as otherwise stated, shape analyses were performed using MorphoJ [44] and the TPS series [46], as described in the main text. Paired Hotelling tests for intratreatment inter-DPI shape change analyses and Mann-Whitney tests for rosette growth analysis were executed in PAST [45]. PTAs based on Adams and Collyer [71] were run in R [72]. Excel 2010 was used for Holm-Bonferroni sequential test for multiple comparisons [76, 77] and hyperellipses calculations using Real Statistics for Excel 2010 (ver. 4.14) [78].

## Results

Morphometrics aims at analyzing the variation and covariation of the size and shape of objects, defining altogether their form. Shape and form might be confusing words, used as synonyms in many languages [13]. Hereafter, the GM definition of shape in the sense [20] that it is “all the geometric information that remains when location, scale and rotational effects are filtered out from an object” is used.

### Landmark configuration, Procrustes fit, and outliers detection

Figure 1A shows an 11-landmark configuration for the Arabidopsis rosette. Plants were inoculated in their third true leaf (24 plants were mock inoculated and 17 were ORMV infected), and images were acquired starting 3 days post-inoculation (DPI) to 12 DPI (see Materials and Methods section).

After executing a full Procrustes fit of each dataset, they were inspected for the presence of outliers. The shape of one mock-inoculated plant (M2) diverted the most from the rest in 11 of 16 datasets and was excluded from all datasets for successive analyses. Thus, 23 mock-inoculated and 17 ORMV-infected plants were used for the subsequent morphometric analyses.

Afterward, datasets were combined and the “Combined dataset 3–12 DPI” was created with 640 observations included following a common GPA. Then, a wireframe was created that connects consecutive landmarks. This tool aids visualization, as will be explained later. Next, the “Combined dataset 3–12 DPI” was subdivided by DPI. This creates one dataset for each DPI; each one has two digitization outputs for each plant.

### Assessment of the tangent (Euclidean) space approximation

By using the combined datasets for mock-inoculated, ORMV-infected, and all plants (see Materials and Methods section), TPSSmall (v.1.33) was run to compare statistics for distance to reference shape both in Tangent (Euclidean) and Procrustes (Kendall’s) shape space for both treatments separately and for all plants together (Supplementary Table S1). The results showed that maximum Procrustes distances from mean (reference) shape were 0.371 (ORMV), 0.405 (mock), and 0.400 (ALL). Mean Procrustes distances from mean (reference) shape were 0.168 (ORMV), 0.186 (mock), and 0.184 (ALL). This indicates a closer arrangement of ORMV shapes in shape space relative to mock-inoculated plants. Tangent and Procrustes distances were highly similar (Supplementary Table S1), and regressions through the origin for distance in tangent space, Y, regressed onto Procrustes distance, X, showed slopes >0.98 and correlations >0.9999 for all groups (Supplementary Table S1, Supplementary Fig. S1). These results are in line with several similar analysis performed on a variety of biological forms [35, 79–81]. Thus, the projections of shapes in Kendall’s shape space onto a tangent Euclidean shape space are good approximations for the studied shapes.

### Testing digitization error and variation between treatments using Procrustes ANOVA

Eight separate hierarchical (Procrustes) ANOVAs were performed to assess digitization error at each DPI. The analysis was executed simultaneously for both size and shape. Results are shown in Supplementary Table S2.

Explained variance (as a % SS) for which individuals accounted was 17.93 to 99.95 for size and 61.00 to 96.74 for shape over all DPIs. By contrast, explained SS (%) for digitization error ranged from 0.01 to 0.12 for size and 0.40 to 1.15 for shape and were almost always two orders of magnitude smaller than individual SS. Thus, digitization error was negligible throughout the digitization process. Furthermore, the results shown in Supplementary Table S2 revealed that for size, the Individual (ID) effect was highly significant at each DPI as evidenced by Goodall *F*test (*P* < 0.0001). Treatment effect was insignificant from 3 to 5 DPI; however, starting from 6 DPI, the virus affected plant size (0.0001 < *P* < 0.03).

**Figure 2:**
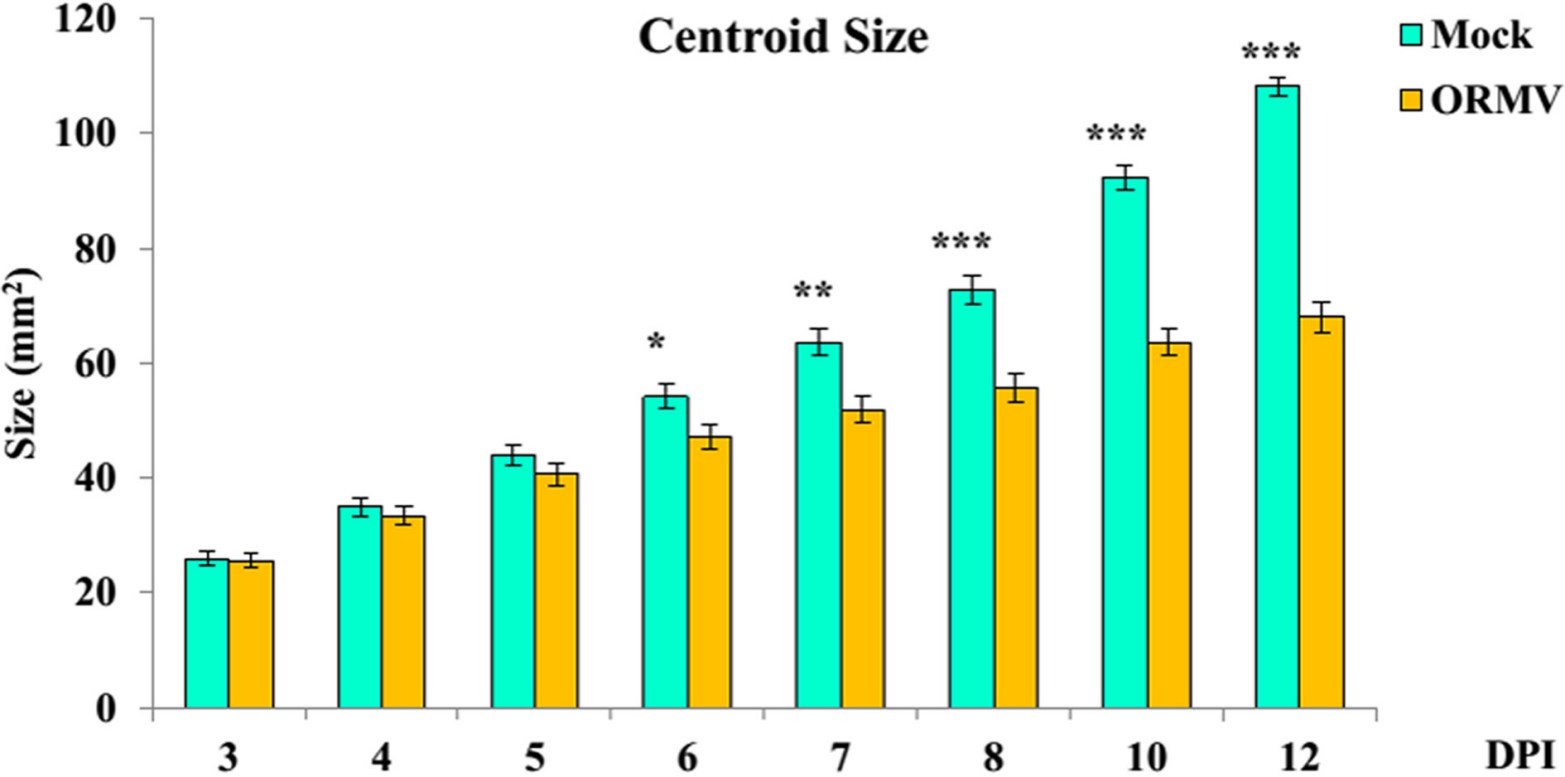
Mean centroid size for mock- and ORMV-inoculated plants across the experiment. Error bars indicate +/- standard error. * = *P* < 0.05; ** = *P* < 0.01; *** = *P* < 0.0001, Mann-Whitney tests.

For shape, similar results were obtained. Indeed, the Individual effect was also highly significant at each DPI as evidenced by Goodall *F*test (*P* < 0.0001) and MANOVA results (*P* < 0.0001). Treatment impacted earlier in shape than size, as differences in shape were evident as soon as 5 DPI (*P* = 0.0008, multivariate test). The infection also had an increasingly proportionally higher impact along the experiment, reaching 82.35 and 38.29 of the explained % SS at the end of the experiment (12 DPI) for size and shape, respectively. Accordingly, ORMV induced a relative growth stagnation that was progressively more accentuated along the experiment (Fig. 2).

### Ordination methods and shape change visualization

#### PCA

**Figure 3:**
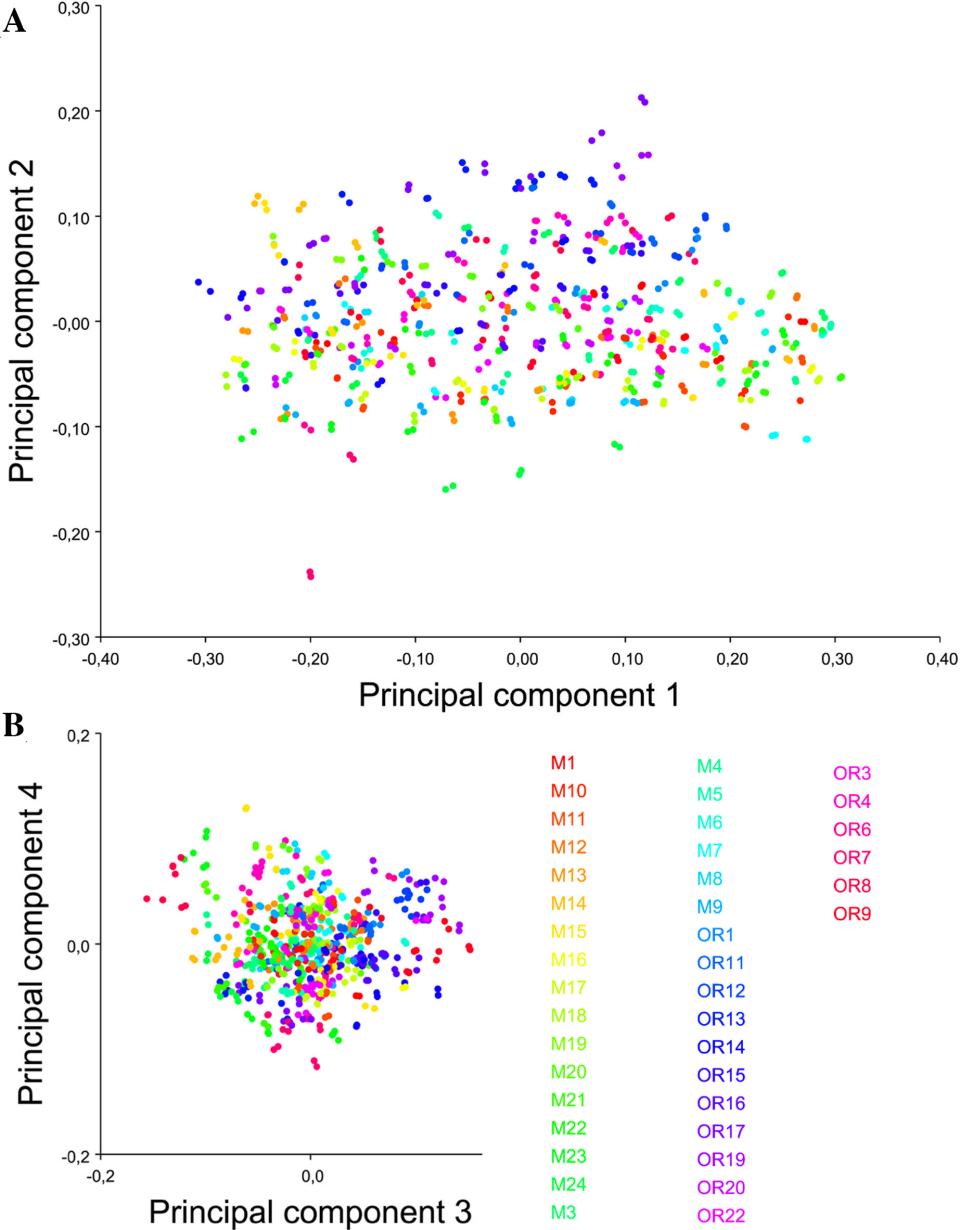
Shape variation including all observations and replicas. PCA scatter plots of **(A)** PC1 vs. PC2 and**(B)** PC3 vs. PC4. Equally colored dots represent both digitizations of the same specimen for all DPIs. The scale factor for this graph is directly the magnitude of the shape change as a Procrustes distance in any given direction; the same scaling was used for all axes.

First, PCA was used to assess error measurement (previously quantified by Procrustes ANOVA; Supplementary Table S2). A covariance matrix was created for the “Combined dataset 3–12 DPI” and then a PCA was performed. Scatter plots were generated for the first four PCs, which together account for 87.2% of total variance (Fig. 3). The proximity of equally colored points indicates a small digitization error.

As digitization error explained a negligible percentage of variance, digitizations were averaged within specimens for each DPI. From the “Combined dataset 3–12 DPI,” the “Combined dataset 3–12 DPI, averaged by ID DPI” dataset was created, which contains all 320 averaged observations. The averaged data were used to find the directions of maximal variance between individuals by requesting a PCA. Three types of graphs were obtained: PC shape changes (a diagram showing the shape changes associated with the PCs), Eigenvalues (histogram showing the percentages of total variance for which the PCs account), and PC scores (scatterp lot of PC scores) (Supplementary File ORMV.morphoJ).

**Figure 4:**
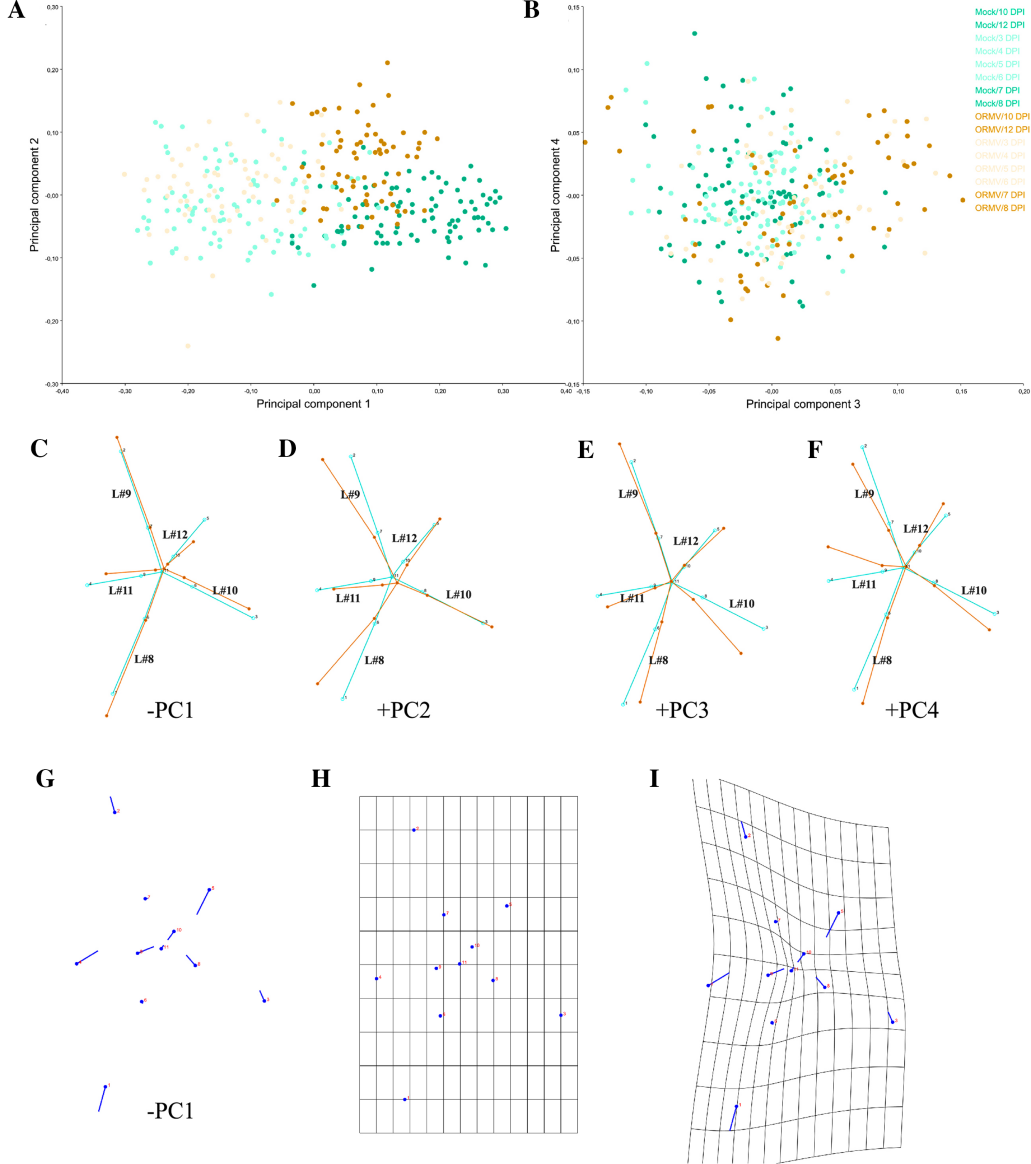
Shape variation between specimens (averaged by measurement replicates). PCA scatterplots of **(A)** PC1 vs. PC2 and **(B)** PC3 vs. PC4, which together explain 87.4% of variance. Pale dots = juvenile (3–6 DPI) plants. Dark dots = mature (7–12 DPI) plants. **(C-F)** Wireframe graphs showing shape changes from the starting (average) shape (bluish green) to the target shape (orange) for the first four PCs. Negative (PC1) and positive (PCs 2–4) components are shown, respectively. Here and throughout this work, leaf number is indicated in the wireframe in black. **(G)** Lollipop graph for the –PC1 component. Lollipops indicate starting position of landmarks with dots. **(H-I)** Transformation grids for **(H)** the starting shape and for **(I)** the target shape (–PC1). Shape changes (C-G and I) are magnified 2x for better visualization.

PC1 and the first four PCs accounted for 64.2% and 87.4% of total variance, respectively. PC scatter plots show specimen distribution along the axes of maximum variance (Fig. 4A, 4B). Dots corresponding to early (3–6 DPI) and later (7–12 DPI) stages were colored in a lighter or darker tone, respectively, to aid visualization. The results showed that PC1 is likely an axis related to development (change in shape related with age), because clearly separated rosettes from early (mostly negative values) to late (positive values) stages of the experiment (Fig. 4A). Moreover, at later stages, ORMV-infected plants had fewer positive scores on this axis; this suggests that infected plants retained a more juvenile (pedomorphic) shape. Positive extremes of PC2–4 are related to ORMV shapes.

Also, by using the shape coordinates of the 320 averaged observations, we then investigated whether plants changed their shapes between successive DPIs within treatments. Intratreatment paired comparisons of shape are possible using a paired Hotelling test (a multivariate analog of the paired *t*test). A strong effect of time on shape was evident from the start of the experiment, and differences were extremely statistically significant for mock plants (Supplementary Table S3). From this point, GM visualization tools are used to better understand what these relative positions on scatter plots mean with respect to shape differences.

#### Visualization of shape changes

Wireframe graphs (Fig. 4C-4F) can be requested for each PC of interest from the “PC shape changes” tab by right-clicking on the displayed image and changing the type of graph. Wireframe graphs connect the landmarks with straight lines for the starting and target shapes by using a previously created Arabidopsis wireframe, thus showing the relative displacements of landmarks from a mean shape. Negative values of PC1 mostly correspond to juvenile (and infected) shapes; positive values of PC1 belong to healthy controls and adults. Hence, by depicting the –PC1 component, target shapes are given negative values (Fig. 4C). The –PC1 explains the relative shortening of leaves #11 (the space limited by landmarks 4, 9 and 11) and #12 (landmarks 5, 10 and 11). This makes sense, since younger plants have yet to develop these relatively new leaves. Petioles of leaves #10, #11 and #12 are particularly relatively shortened. Relative to these shortenings, older leaves (#8 and #9) are longer but, interestingly, only its laminae, since its petioles are not relatively elongated. Taken together, PC1 reveals that ORMV impaired the elongation of newer leaves to their normal extent. PC2 (Fig. 4D) associates with relative radial displacements of leaves; tips of leaves #8 and #9 (landmarks 1 and 2) come close together, lowering the typical angle between successive leaves from near 137.5° to close to 90°. These relative displacements determine that leaves #9 and #10 form an exaggerated angle of near 180°. PC3 (Fig. 4E) is also mostly associated with radial changes in the infected rosette: leaf #10 is relatively displaced towards leaf #8 and the main effect is, again, the increase of the angle between leaves #9 and #10 to near 180°. PC4 (Fig. 4F) explains less proportion of total variance (4.5%) and is mostly related to the relative displacement of the lamina of leaf #11 toward leaf #9 almost without altering its petiole, which functions as a hinge. Leaves #9 and #10 are, as a combination of the effects depicted by PC2 and PC3, both relatively displaced toward leaf #8. Together, the wireframe visualization of the first four PCs (which account for more than 87% of total variance) shows that ORMV induces the relative shortening of laminae and (especially) petioles of the newest leaves. This shortening is related to a pedomorphic shape. Furthermore, this analysis also demonstrates that ORMV provokes the relative displacement of leaves #9 and #10 toward leaf #8.

Displacement vectors (called “lollipop graphs” in MorphoJ) are arrows drawn between a landmark in a starting shape and the same landmark in a target shape. The dot in the lollipop represents the starting position, and the vector is represented by a line departing from it (but in some software, the inverse convention is followed, i.e., PAST). Although these visualizations are being displaced in the GM literature in favor of more advanced tools [69], here, the case for –PC1 is presented, showing the relative displacements of landmarks (Fig. 4G). It can be directly compared with Fig. 4C.

Finally, Fig. 4H and 4I show exemplified transformation grids for –PC1. Figure 4H depicts the starting (mean) shape, whereas Fig. 4I shows the transformed grid for –PC1. The compression of the grid in the central zone is the result of the relative displacement of the space between landmarks 3, 8, and 11 (leaf #10); 4, 9, and 11 (leaf #11); and 5, 10, and 11 (leaf #12) toward the center of the rosette. In addition, grid stretching is detected around landmarks 1 and 2 and reveals the relative expansion of laminae of leaves #8 and #9, since its petioles remain relatively immobile; landmarks 6 and 7). As stated previously, visualization with these grids should be cautiously interpreted since the interpolation function deforms the grid between places where no landmark is placed (and no information about even the existence of tissue is guaranteed). Therefore, such visualizations need to be interpreted cautiously in regions that are relatively far from landmarks [69]. To assess these changes in more detail, PCA analyses were performed for each DPI. The “Combined dataset 3–12 DPI, averaged by ID DPI” was subdivided by DPI performing a common Procrustes fit, thus creating eight new datasets (DPIs) (raw data in Supplementary File ORMV.morphoJ). Covariance matrices were generated, and a PCA was performed for each DPI dataset. PC1 and the first four PCs accounted for 27% to 43% and 78% to 84% of total variance, respectively. PCs beyond PC4 accounted for 5% or less of variation each. Shape change visualization showed that PC1 gradually separated specimens belonging to different treatments. Mock-inoculated plants were progressively more aligned with positive PC1 values. PC2 was more generally related to ORMV-infected plants in its positive values. Relative shortening of younger leaves and petioles and relative displacement of leaves towards leaf #8 were progressively more accentuated (Supplementary Fig. S2).

#### Discriminant analysis

**Figure 5:**
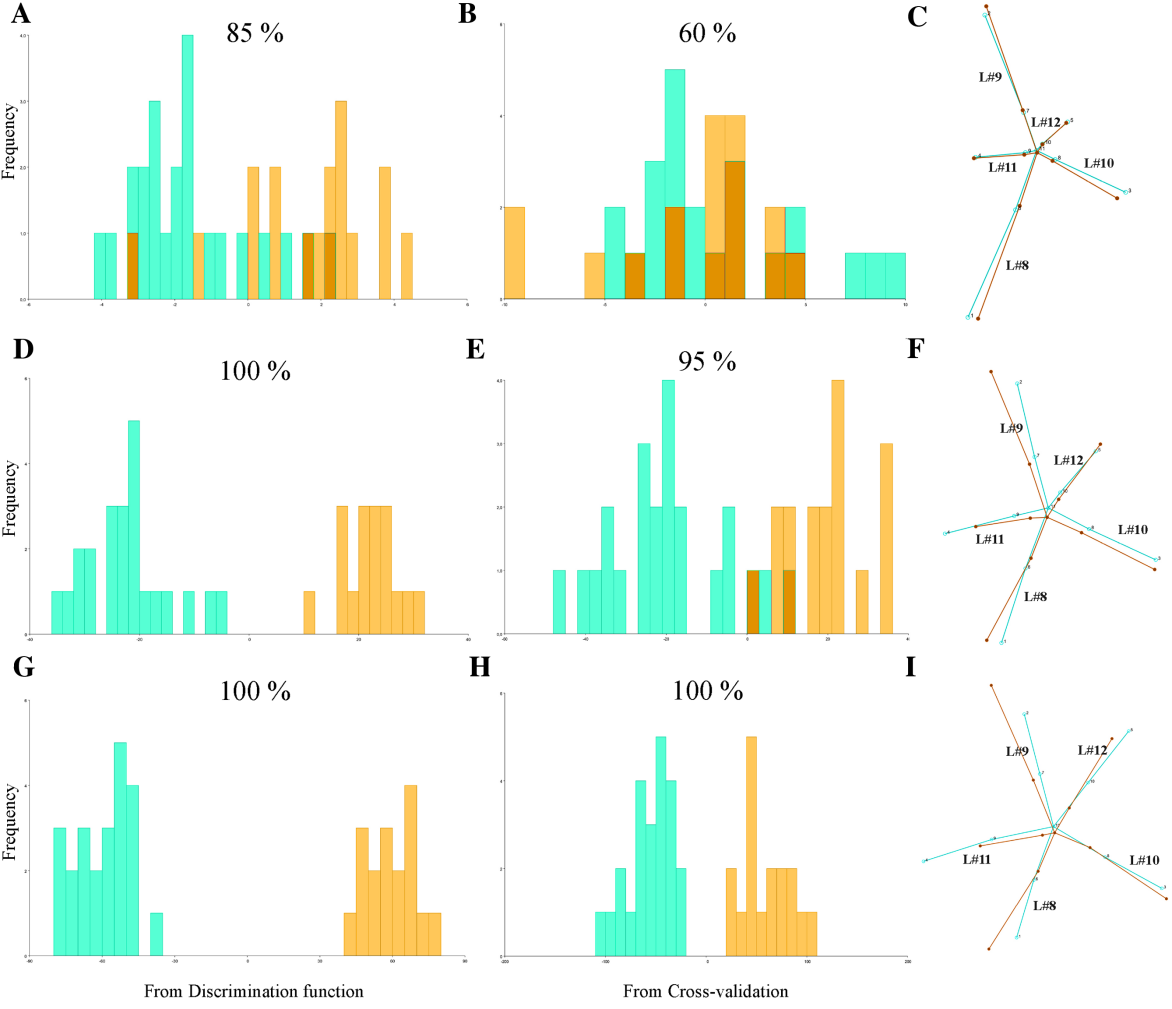
Discriminant analyses of shape variation between treatments at 3 **(A- C)**, 7 **(D-F)**, and 12 **(G-I)**DPI. Frequencies of discriminant scores obtained by resubstitution rates of assignments **(A, D, G)** and a jackknife cross-validation **(B, E, H)** are shown using histogram bars with percentages of correct assignments above each graph. Wireframes comparing mean shapes **(C, F, I)** are shown magnified 2 times. Mock = bluish green; ORMV = orange.

So far, distances between individuals were addressed with the aid of PCA. Subsequently a DA was performed to test whether differences between treatments are detectable at each DPI (Table 1). At 5 DPI, the three tests detected shape differences between treatments (0.001 < *P*< 0.005). From 6 DPI and beyond, *P* values were extremely significant (*P* < 0.0001). These results coincide with those obtained by Procrustes ANOVA of shape (Supplementary Table S2). DA maximizes group separation for plotting their differences and predicting group affiliation (classification). The classification of a given specimen (through the discriminant axis) is done using functions that were calculated on samples that included that same specimen (resubstituting rate of assignment). Then, a degree of over-fitting is unavoidable and leads to an overestimate of the effectiveness of the DA. To overcome this problem, one can use a cross-validation or jackknife procedure [35, 43]. A jackknife procedure leaves one specimen at a time not used for constructing the discriminant function and then tests the rate of correct specimen assignment. Only jackknife cross-validated classification tables provide reliable information on groups. Figure 5 displays DA results in group assignment for 3, 7, and 12 DPI, and Supplementary Table S4 details these results for all DPIs. As expected, resubstitution rates of assignment (Fig. 5A, 5D, 5G) were higher than jackknifed counterparts (Fig. 5B, 5E, 5H) but those jackknifed reached high levels of accuracy (≥90%) from 6 DPI and later (Supplementary Table S4). To test whether this level of accuracy was adequate, these results were compared with classification/misclassification tables completed by human observers. The entire image dataset of 7 DPI was given to three expert researchers working with Arabidopsis (one of the authors [S.A.] and two other researchers from another institution). They did not know which plants were mock-inoculated or ORMV-infected, except for one mock-inoculated and one ORMV-infected plant that were given as phenotypic references. These two reference plants were excluded from the dataset for subsequent human classification. The researchers classified the 38 remnant plants (Supplementary Table S4). Human accuracy ranged from 55% to 72.5%, with an average of 64.2%. Therefore, DA outperformed the expert human eye by 30% at 7 DPI and yielded higher classification rates from 5 DPI.

**Table 1:** Statistical tests for differences between means of treatments at each DPI from DA

Discriminant function analysis	3 DPI	4 DPI	5 DPI	6 DPI	7 DPI	8 DPI	10 DPI	12 DPI
Difference between means								
Procrustes distance	0.037	0.047	0.063	0.087	0.097	0.105	0.149	0.189
Mahalanobis distance	1.799	1.924	3.815	5.117	6.651	7.035	9.078	10.863
T-square	31.637	36.170	142.264	255.916	432.438	483.790	805.573	1,153.389
*P value (parametric)*	0.521	0.405	0.001	<0.0001	<0.0001	<0.0001	<0.0001	<0.0001
*P* values for permutation tests (1,000 permutation runs)								
*Procrustes distance*	0.549	0.182	0.005	0.002	<0.0001	<0.0001	<0.0001	<0.0001
*T square (Mahalanobis distance)*	0.523	0.417	0.001	<0.0001	<0.0001	<0.0001	<0.0001	<0.0001

Permutation tests with 1000 random runs

Wireframe graphs for 3, 7, and 12 DPI (Fig. 5C, 5F, 5I) show the difference from mock to ORMV group. The difference was subtle at 3 DPI, if there was any (Fig. 5C), consistent with nonsignificant differences found by DA at this stage. At 7 DPI (Fig. 5F), the relative shortening of leaf #11 (landmarks 4, 9, and 11) and the relative increase in the angle between leaves #9 and #10 were evident. These tendencies persisted at 12 DPI (Fig. 5I). At this stage, petioles of leaves #11 and #12 were strongly relatively shortened. These results resembled those shown in Fig. 4C-4F and approximately summarize shape changes explained by the first four PCs. This indicated that these shape differences not only separated juveniles from adults but also are hallmarks of shape change induced by ORMV. These results are interesting because discriminant axes not necessarily resemble PCA axes [43].

### Allometric patterns and size correction

As ORMV induced not only changes in shape but also in size (Supplementary Table S2), it is worth investigating whether shape differences between treatments (within a given DPI) are associated with size differences. In principle, group differences could arise if individuals of one group are different in shape because they grew faster than those from the other group and reached earlier a more advanced developmental stage.

Allometry analyses were performed with individual datasets (each corresponding to one separate treatment for each DPI) from the first digitization (as proven earlier [Fig. 3, Supplementary Table S2], differences between digitization instances were negligible).

**Figure 6:**
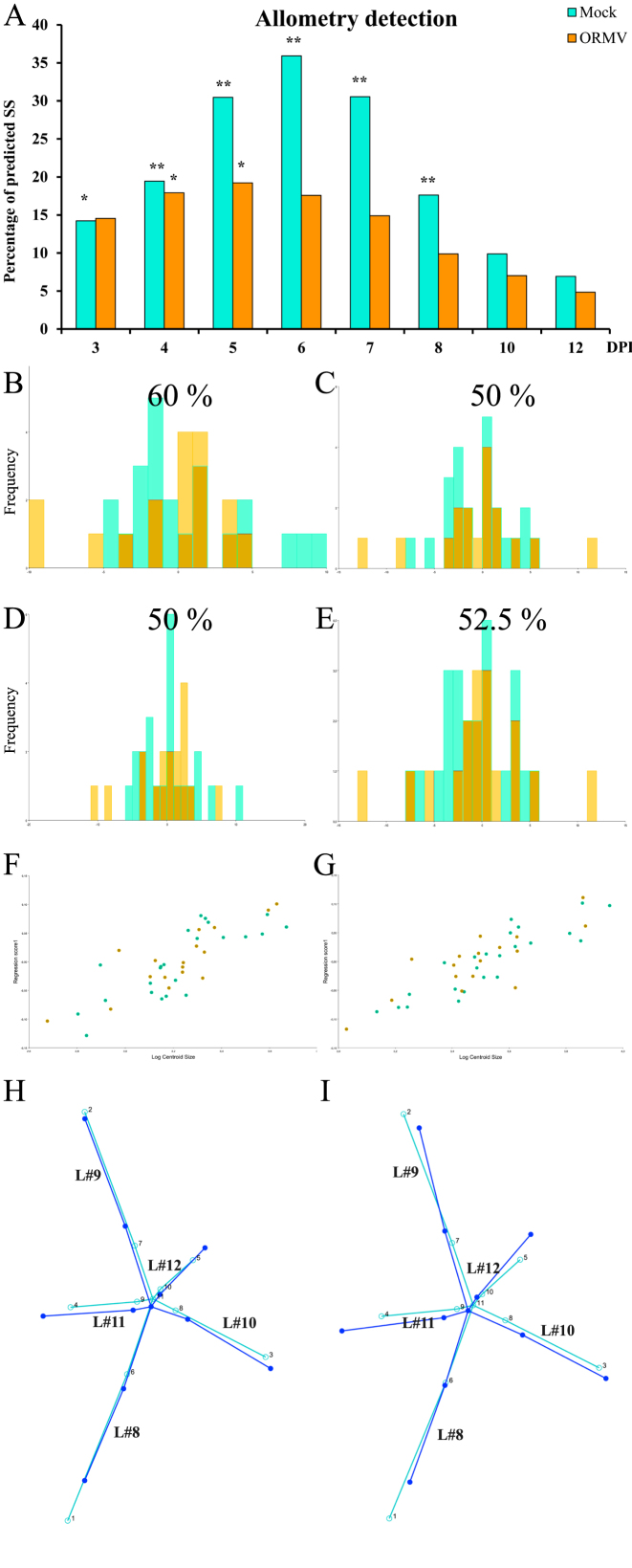
Allometric analyses. **(A)** Predicted SS from regressions of shape onto ln(CS) for each treatment and DPI. *P* values were corrected using Holm sequential test (α = 0.05). * = *P* < 0.05; ** = *P* < 0.01. Allometric analyses for **(B, D, F, H)** 3 DPI and **(C, E, G, I)** 4 DPI (mock = bluish green; ORMV = orange). Cross-validated DAs before **(B-C)** and after **(D-E)** size correction with percentages of correct assignments above each graph. **(F-G)** Scatter plot of regression scores vs. ln(CS). **(H-I)** Wireframes showing starting mean shape (turquoise) and target shape depicting an increase in one unit of ln(CS) (blue), without magnification.

Figure 6A and Supplementary Table S5 show groups with statistically significant allometry and predicted SS from regressions (which correspond to allometric variation of shape). Allometry accounted for a moderate to high proportion of the total shape variation, since SS reached values of 36% at 6 DPI (mock). ORMV induced a reduction in the allometric component of shape variation, as evidenced by lower predicted SSs throughout the experiment and nonsignificant values of allometry for all except 4 and 5 DPI. For both treatments and particularly for healthy controls, a bell-shaped curve was detected. A maximum allometry was detected at 6 DPI for mock plants, but a day before for ORMV. Differences between treatments started at 5 DPI, when allometry accounted for 32% and 20% of predicted SS for mock and ORMV, respectively. This analysis shows that shape variation is much less driven by size heterogeneity (at a given DPI) in ORMV plants. On the other hand, for mock plants this situation (isometry) occurs at later stages of development (10–12 DPI).

Allometry was detected from 3 to 8 DPI. For this reason, TPSRegr (v. 1.41) was used first to determine whether treatment-specific slopes were parallel at each DPI (3 to 8) (Supplementary Table S5). This phenomenon only occurred 3 and 4 DPI (*P*> 0.05, nonstatistically significant slope differences). As slopes were found to be parallel, it is possible to test whether they are separate parallel slopes or coincident (same Y intercept). TPSRegr tests demonstrated that slopes were coincident (*P*> 0.05). Then, size corrections could only be done for 3 and 4 DPI, since from 5 to 8 DPI, slopes were different (*P*< 0.05) and groups follow their own allometric pattern. Also, for 10 and 12 DPI, there is isometry and size does not correlate with shape variation. Size correction was done for 3 and 4 DPI separately in MorphoJ using all 40 plants. Shape variables were regressed onto ln(CS) for each dataset by pooling regressions within subgroups (treatments), and permutation tests with 10,000 runs were requested. Residuals from the analyses contain the size-free information about shape only and can be used to repeat DAs to test for improved accuracy of discrimination [70]. Group separation was not improved (Fig. 6B-6E). This is somehow expected since at this stage of the viral infection, differences in size or shape are undetectable (Supplementary Table S2, Table 1, Fig. 5). This test and the large overlap between populations in the scatter plot of regression scores on size (Fig. 6F, 6G) suggest that the effect of size on shape is very similar for both treatments and DPIs. Bigger rosettes have further distal displacements of leaves 10, 11, and 12 relative to older leaves (8 and 9) and elongated petioles (Fig. 6H, 6I), thus reflecting the differential internal growth of the rosette. Bigger, more mature rosettes have more developed newer leaves.

### PTA and morphospace occupation patterns

**Figure 7:**
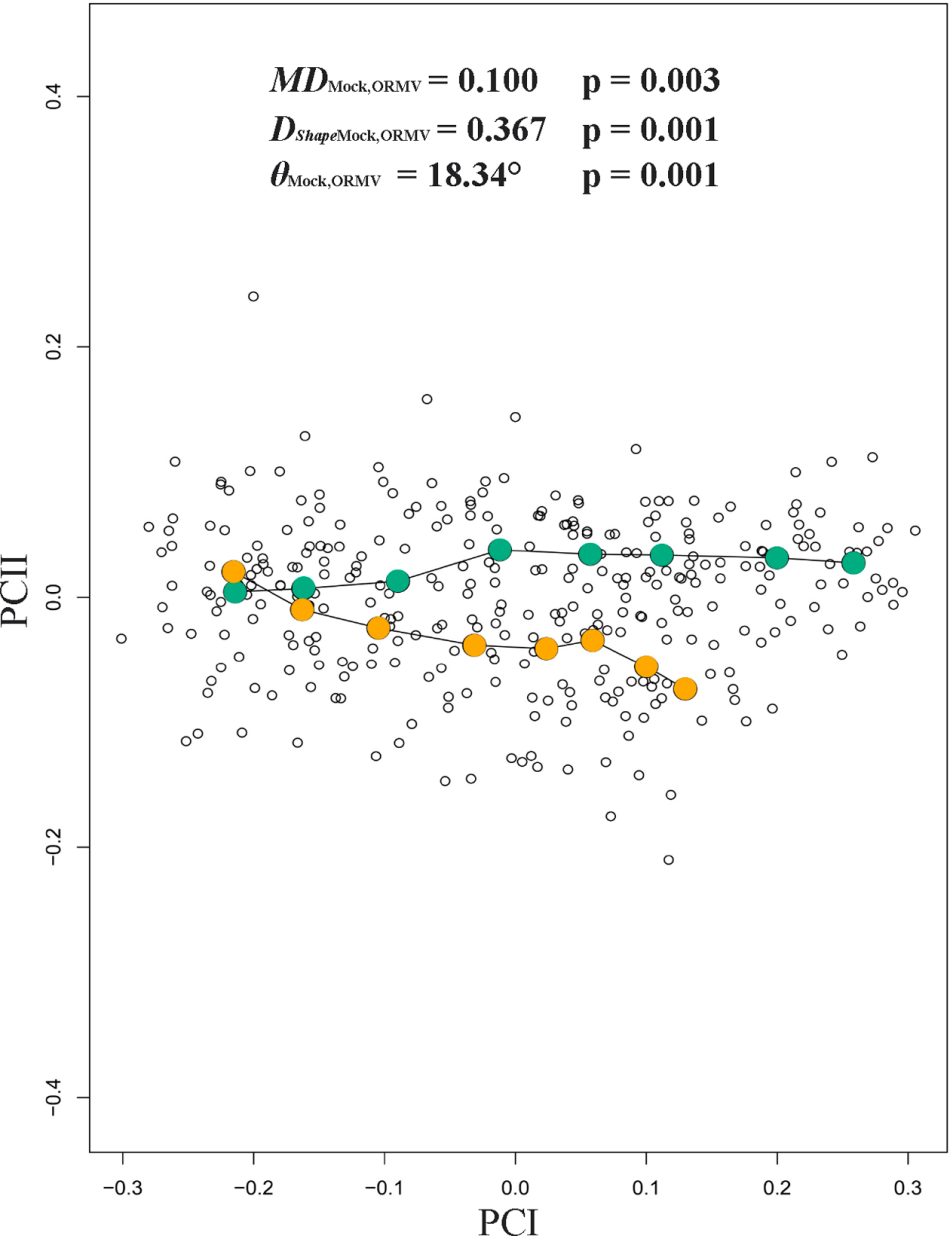
Phenotypic trajectories for mock and ORMV (3–12 DPI). Scatter plot shows the first two PCs of shape variation across the experiment. Mean values for each DPI are colored and connected with lines. PTA parameters are given (see Materials and Methods section). Mock = bluish green; ORMV = orange.

PTA approach (with 1,000 residual randomization permutations) revealed significant differences in the magnitude of phenotypic evolution between the two treatments (*MD*_Mock, ORMV_ = 0.100, P*_size_* = 0.003). This implies that ORMV-infected plants experienced a lower rate of ontogenetic phenotypic evolution compared to controls. Overall direction of ontogenetic changes was also statistically significantly different (*θ*_Mock, ORMV_ = 18.34°, P*_θ_* = 0.001). Finally, shape assessment analysis showed differences between treatments regarding trajectories over time (*D_Shape_*_Mock, ORMV_ = 0.367, P_Shape_ = 0.001) (Table 2). Accordingly, when phenotypic trajectories were plotted over time through a projection of the first two generated PCs on a plane (Fig. 7), it was found that mock and ORMV plants follow different trajectories across the morphospace. Beyond 6 DPI, ORMV induced a relative stasis along PC1 (major morphological axis).

**Table 2:** Comparative trajectory analyses for the full dataset of the ORMV experiment (3–12 DPI), the reduced dataset (4–10 DPI), and the comparisons with TuMV experiments (4–10 DPI)

ORMV3–12 DPI^(a)^		*P value*
*MD_Mock,ORMV_*	0.100	**0.003**
*θ_Mock,ORMV_*	18,34°	**0.001**
*D_ShapeMock, ORMV_*	0.367	**0.001**
MPL_Mock_	0.696	**2.50E-04**
MPL_ORMV_	0.596	
∑Var _Mock_	0.035	**2.52E-06**
∑Var_ORMV_	0.023	
D1_(Mock)_	20.21	**2.89E-06**
D1_(ORMV)_	26.93	
Hyperellipse_(IC95%)Mock_	0.022	**0.005** *
Hyperellipse_(IC95%)ORMV_	0.014	
D2_(Mock)_	32.97	**0.040** *
D2_(ORMV)_	41.87	
**ORMV4–10 DPI** **^(a)^**		
*MD_Mock,ORMV_*	0.085	**0.005**
*θ_Mock,ORMV_*	16,46°	**0.001**
*D_ShapeMock, ORMV_*	0.343	**0.037**
MPL_Mock_	0.472	**8.03E-04** *
MPL_ORMV_	0.401	
∑Var_Mock_	0.022	**2.21E-05** *
∑Var_ORMV_	0.015	
D1_(Mock)_	21.77	**3.61E-05** *
D1_(ORMV)_	28.37	
Hyperellipse_(IC95%)Mock_	0.012	0.075*
Hyperellipse_(IC95%)ORMV_	0.009	
D2_(Mock)_	48.94	0.203*
D2_(ORMV)_	56.41	
**TuMV4–10 DPI 1st** **^(b)^**		
*MD_Mock,TuMV_*	0.093	**0.015**
*θ_Mock,TuMV_*	34,41°	**0.001**
*D_ShapeMock, TuMV_*	0.613	**0.001**
MPL_Mock_	0.504	**0.049** *
MPL_TuMV_	0.461	
∑Var_Mock_	0.030	**0.007** *
∑Var_TuMV_	0.023	
D1_(Mock)_	16.94	**0.017** *
D1_(TuMV)_	21.83	
Hyperellipse_(IC95%)Mock_	0.019	0.156*
Hyperellipse(IC95%)_TuMV_	0.017	
D2_(Mock)_	32.51	0.277*
D2_(TuMV)_	46.05	
**TuMV4–10 DPI 2nd** **^(c)^**		
*MD_Mock,TuMV_*	0.082	0.202
*θ_Mock,TuMV_*	35,05°	**0.001**
*D_ShapeMock, TuMV_*	0.642	**0.002**

* = First three PCs considered (>95% total variance).

Units: MD = D_Shape_ = MPL = D1 = D2 = Euclidean distance. θ = degrees. ∑Var = Hyperellipse_(CI = 95%)_ = dimensionless.

Statistically significant results in **bold**.

(a) N = 23 (mock) and 17 (ORMV)

(b) N = 27 (mock) and 14 (TuMV)

(c) N = 14 (mock) and 8 (TuMV)

Regarding differences in morphospace occupation patterns, the morphometric change experienced by a plant throughout ontogeny equals the Euclidean distance (D) between successive points in a morphospace that represents its shape at each DPI. As PCs from a PCA carry all the morphological information extracted from the Procrustes coordinates, distances are simultaneously calculated over all the PCs by using the Pythagorean theorem. These distances are designated as morphometric path lengths (MPL) (ΣD = MPL) (sensu [75]). Mock-inoculated plants traveled on average more distance through morphospace than infected plants (MPL_Mock_ = 0.6956 vs. MPL_ORMV_ = 0.5963, *P* = 0.00025, Mann-Whitney test). Other measures are traditionally used to detect changes in morphospace occupation patterns and the amount of the difference between character states among specimens in morphospace [74], e.g., sum of variances (∑Var). Control plants had higher ∑Var values than infected plants (∑Var _Mock_ = 0.0350 vs. ∑Var_ORMV_ = 0.0230, *P* = 2.52 × 10^−6^, Mann-Whitney test). This result suggested a higher increase in shape change in controls [74]. Morphospace density occupation measures could be obtained taking into account not only MPLs but variances of the PCs across the experiment. If a group folded an equivalent amount of morphometric change into a much smaller region of morphospace than another, then it had a higher density [75]. Morphometric path density (D) could be calculated as D_1_ = MPL/∑Var. ORMV-infected plants are more densely restricted in morphospace (D_1(Mock)_ = 20.21 vs. D_1(ORMV)_ = 26.93, *P* = 2.89 × 10^−6^, Mann-Whitney test) (Table 2).

An alternative measure of density (D_2_ = MPL/V) considers the volume (V) that the group occupies in morphospace. Several volumetric measures are possible [74]. This study considered the volume of a 95% confidence hyperellipse. D_2_ was obtained by calculating the square root of the product of the Eigenvalues of the PCs and comparing them with expected values for a X^2^ distribution at α = 0.05. Mock-inoculated plants have hyperellipses of higher volume on average (Hyperellipse_(IC95%)Mock_ = 0.0129 vs. Hyperellipse_(IC95%)ORMV_ = 0.0073), although the differences were not statistically significant (*P* = 0.11888, Mann-Whitney test). Similarly, density measures based on hyperellipses calculations were not statistically significantly different (D_2(Mock)_ = 111.47 vs. D_2(ORMV)_ = 146.34, *P* = 0.25051, Mann-Whitney test), although ORMV-infected plants had a higher average density. These differences could be because hypervolume calculations can produce extremely small and variable values. The hypervolume is calculated by taking the product of univariate variances; thus, any axis or axes with negligible variance will produce a hypervolume value close to zero. Moreover, all multiplied variances are given the same weight; onsequently, PC axes representing a minimal percentage of the total variance could distort conclusions obtained with more informative axes. Thus, hypervolume can be very sensitive to variation in a single character. To avoid this, one must select only the axes with significant variances to represent the disparity among points in morphospace [74]. Therefore, the analysis was repeated including only the first three PCs, which accounted for more than 95% of variance. The results were similar to those previously found for all the parameters, but with significantly different hyperellipse volumes (Hyperellipse _(IC95%)Mock_ = 0.022 vs. Hyperellipse_(IC95%)ORMV_ = 0.014, *P* = 0.0052597, Mann-Whitney test) and D_2_ parameters (D_2(Mock)_ = 32.97 vs. D_2(ORMV)_ = 41.87, *P* = 0.040172, Mann-Whitney test) (Table 2).

Together, PTA and morphospace occupation patterns showed that mock-inoculated and ORMV-infected plants follow separate paths through morphospace. They differ in length, direction, and shape (Fig. 7). They also explore distinct regions of morphospace in a disparate quantity. Control plants experience more diversification of shape, as evidenced by the comparative length of trajectories (*MD* and MPL), have a higher amount of difference between shape states in morphospace (∑Var) throughout the experiment, and explore larger regions of morphospace (D_1_, D_2_) (Table 2). Thus, ORMV infection not only alters the direction of ontogenetic shape development but also diminishes shape change.

### Comparison with TuMV infections

**Figure 8:**
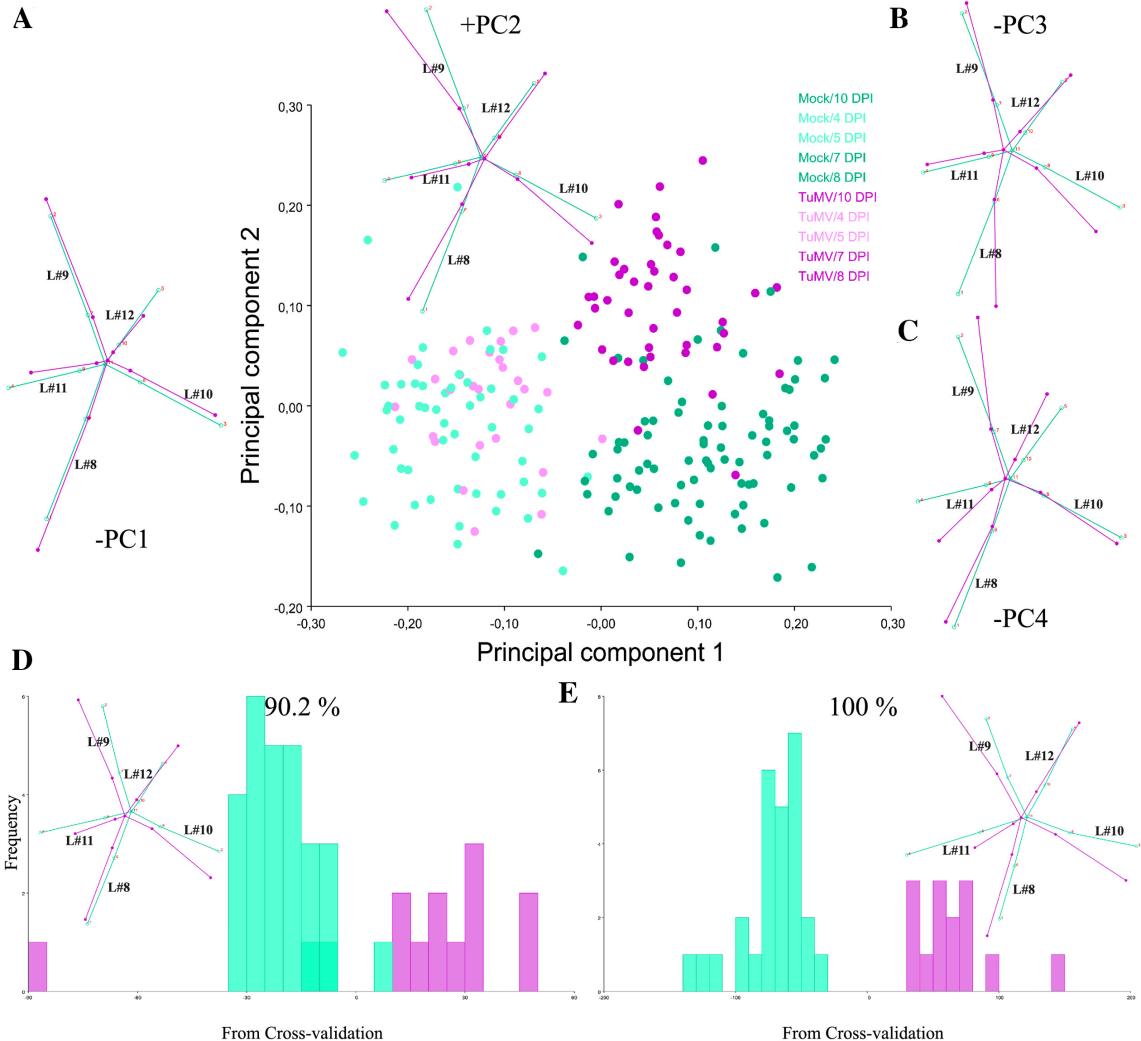
Summary of GM analyses for TuMV-infected plants. **(A-C)** Shape variation between specimens. **(A)** PCA scatter plot (PC1 vs. PC2). Pale dots = juvenile (4–5 DPI) plants. Dark dots = mature (7–10 DPI) plants. Wireframe graphs from starting (average) shape (bluish green) to target shape (reddish purple) corresponding to –PC1 (to the left) and +PC2 (top) are included. **(B-C)** Wireframes for –PC3 and –PC4, respectively. **(D-E)** Frequencies of jackknifed discriminant scores for 7 and 10 DPI, respectively, with wireframes depicting shape changes included. Wireframes show starting shape (mock = bluish green) to the target shape (TuMV = reddish purple). Shape change is magnified 2x.

**Figure 9:**
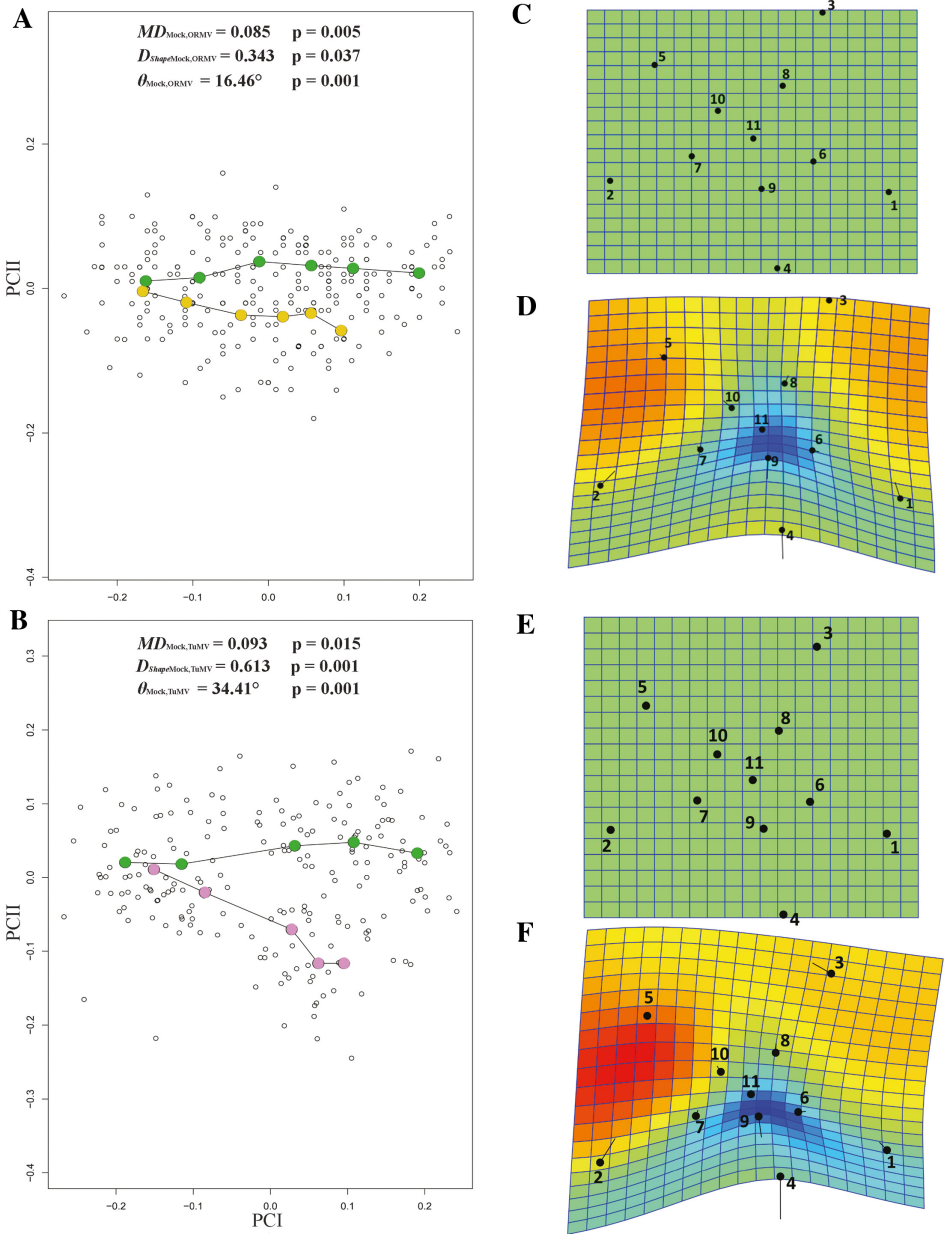
Comparison of virus severity. PC plots of PTA for **(A)** ORMV- and **(D)** TuMV-infected plants compared with mock-inoculated plants (4–10 DPI). PTA parameters are shown (see main text). Transformation grids with lollipops and Jacobian expansion factors were executed in PAST [45] for ORMV- and TuMV-infected plants depicting (mean) shape change from controls to virus-infected plants (B to C and E to F, respectively) at 10 DPI. Jacobian expansion factors indicate expansions of the grid (yellow to orange red for factors >1) or contractions (blue for factors between 0 and 1). The same color scale was set for both comparisons. Lollipops indicate target position of landmarks with dots. Leaf 11 (landmarks 4, 9, and 11) is positioned at the bottom.

One goal of applying the GM approach to Arabidopsis studies is to make more objective and repeatable phenotypic comparisons. To this end, the same experimental setup was applied to study viral infections of *A. thaliana* with TuMV, an ssRNA+ virus unrelated to ORMV [82]. The experiment spanned from 4 to 10 DPI. The time point at 12 DPI was discarded because excessive curling of some leaves owing to TuMV infection impaired the correct assignment of landmarks (Supplementary File TuMV 1st.morphoj). Individual datasets were created for each DPI, and Procrustes coordinates were extracted. A combined dataset was created, and PCA was performed. After the exclusion of outliers, 27 mock- and 14 TuMV-inoculated plants remained. PCA revealed that PC1 accounted for 49.2% of total variance (much less than in the ORMV experiment) and that PC1 plus PC2 accounted for 69.3% of total variance. Again, PC1 mostly separates juveniles from adult rosettes, and negative values related predominantly to infected plants, which retained a more immature phenotype (Fig. 8A). This result was supported by the associated wireframe graph, which depicts a relative shortening of leaves 11 and 12, similar to ORMV-infected plants (Fig. 4C). PC2 was strongly positively related to infected plants and, similar to the ORMV case (Fig. 4D), reflected the widening of the angle between leaves 9 and 10. PCs 3 and 4 (Fig. 8B, 8C) accounted for 17.7% of total variance and were mainly negatively related to TuMV infection. DSA (Fig. 8D, 8E) showed that, as with ORMV, group means were statistically significantly different from 5 DPI. Wireframe graphs also evidenced a strong relative shortening of the petioles, as in ORMV infections (Fig. 5F, 5I). This indicates that more compact rosettes are a common outcome of these viral infections. Discriminant power was slightly higher for almost all DPIs in the case of TuMV (Supplementary Tables S4 and S6). Moreover, Procrustes distances were longer for every DPI in the case of TuMV, which induced a Procrustes separation at 8 DPI that only matched at 12 DPI with ORMV-infected plants (Table 1, Supplementary Table S6). These results suggest that in Arabidopsis, TuMV is a more severe virus than ORMV since it induces a more pronounced departure from mock mean shape. PTA supported this evidence, as evidenced by a subset of 4–10 DPI datasets selected to compare ORMV with TuMV infections (Fig. 9A, 9B, Table 2). Whereas the trajectory size difference in TuMV-infected plants (*MD*_Mock, TuMV_) was similar to that obtained in ORMV-infected pants, the multivariate angle (*θ*_Mock, TuMV_) that separates infected (TuMV) from healthy trajectories more than doubled that of the experiment with ORMV. Shape differences (D*_Shape_*_Mock, TuMV_) between trajectories almost doubled. Similar to ORMV infection, most of the other measures indicated a slower rate of shape change compared to mock plants (Table 2). To visualize and compare shape changes in the ORMV and TuMV experiments, transformation grids with Jacobian expansion factors and lollipops were performed in PAST for 10 DPI plants (Fig. 9C-9F). Both viruses induced relative contraction of the rosette around leaf 11 (the most affected), but TuMV induced more severe deformations. To confirm these results and to test reproducibility, we carried out an independent experiment of TuMV infection (Supplementary File TuMV 2nd.morphoj). PTA analyses were run and trajectory attributes compared (Table 2). The results were similar to those of the first TuMV experiment.

Together, these results indicated that both TuMV and ORMV induced relative developmental arrest as well as shape change. However, ORMV triggers symptoms that are mainly driven by developmental arrest, whereas TuMV also promotes a higher shape change that impacts more strongly on the overall shape.

## Discussion

Here, several GM tools were applied to assess morphological changes induced by viral infections in Arabidopsis. The GM analysis is a powerful approach owing to its statistical toolbox and its appealing visual analysis of shape change. By conceptually separating size and shape, both factors that determine form could be separately analyzed. Thus, the effect of ORMV infection was detected earlier in shape than in size (Table 1, Supplementary Table S2, Fig. 2). GM analysis greatly outperformed diagnosis when compared with the expert human eye (Supplementary Table S4). The effect of time on shape was more pronounced than that of treatment, since the former was detected earlier (Supplementary Tables S2 and S3). This was particularly the case for control rosettes, indicating that normal rosette development is not a scaling up of previous shapes but a relative displacement of newly developed structures. This process is somewhat impaired by ORMV, which induced the retention of a more juvenile-like phenotype (Fig. 4).

Normal allometric growth comprised a lengthening of petioles and laminae of new leaves (11 and 12) relative to older ones (Fig. 6H, 6I). This process was reversed by ORMV, which also distorted the normal angle of approximately 137.5° between successive leaves. As a result, leaves 9 and 10 were relatively bent toward leaves 8 and 11, which in turn came close together and bent toward the inoculated leaf (3) that is mid way between them (Fig. 5F, 5I). TuMV provoked similar outcomes (Fig. 8) but with an apparent stronger effect, not only regarding the distorted inter-leaves angle but for the relative contraction of leaf 11 with respect to all remaining leaves, including leaf 12 (Fig. 8E, 9D, 9F). It is important to note here that we did not perform absolute individual angle or distance measurements. This kind of traditional morphometric measurement could also be manually taken by using TPSDig2 software and would be an addition to the parameters presented here, where the focus is only on geometric morphometric tools. However, the normal leaf phyllotactic pattern (the arrangement of organs in regular patterns around the stem of a plant) seems not to be changed by these viruses (personal observations, data not shown). Instead, the distortion of the angle determined by the tip of two successive leaves (with its vertex in the center of the plant) appears to arise from the relative outgrowth of the distal part of the lamina. Taking into account the source-to-sink nature of viral movement by phloem [39] and its radial structure [83], it could be hypothesized that virus or viral-induced signals are distributed through the rosette in such a way that they inhibit proximal systemic growth. Future work should test this hypothesis by comparing cell number or size in distal and proximal parts of systemic leaves or by assessing the effect that growth hormones and mutants have on these parameters. This kind of data-based hypothesis is an example of a desirable outcome of the application of GM tools [43] in particular and of phenotyping in general. In this study, the observance of the relative shortening of petioles or laminae and the distorted inter-leaves angles could lead to additional experiments to more precisely quantify these discrete phenotypes using traditional morphometric measurements. These traditional measurements, however, should be made taking into account their intrinsic statistic limitations (see [43], Introduction).

Both viruses diminished shape change by constraining virus-infected rosettes to smaller regions of multivariate morphospace (Supplementary Tables S1 and S2, Fig. 7 and 9). Ontogeny (the development or course of development of an individual organism) is a genetically based endogenous process that can be altered by the environment [84]. Here, both viruses induced the departure of normal ontogenetic development. The consequences of this departure should be further analyzed by measuring relevant traits.

An objective measurement unit of shape change (Procrustes distance) allowed us to compare ORMV- and TuMV- induced shape changes relative to the departure from healthy control shapes (Tables 1 and 2, Supplementary Tables S2 and S6, Fig. 9) and objectively rank symptom severity. In addition, visualization tools aided us in identifying where to allocate the shape change differences in the rosette (Fig. 5C, 5F, 5I, 8D, 8E, 9D, 9F). In sum, TuMV impacts more strongly on Arabidopsis rosette shape than ORMV.

In this work, two experiments with TuMV were performed to investigate reproducibility. The second experiment was carried out with roughly half the specimens (Table 2). Whereas PTA parameters were very similar between experiments, *P* values tend to show less statistical power associated with the smaller sample (Table 2). This points to both the robustness of the effect found (PTA parameters) and the need to have a minimum sample size to statistically assess shape differences, an issue that will reasonably be of more concern when studying more subtle effects. Although sampling problems arising from scarcity of specimens is certainly not a problem in Arabidopsis studies, having a large number of plants could indeed pose a problem because of the limited room in expensive growth chambers that is needed to perform physiological experiments in a highly controlled environment. Minimum sample size estimation is not trivial, because it may vary depending on the natural shape variation within the assessed population and the kind of scientific question being addressed [85]. Future work should investigate the effect of sample size onto statistical parameters, since this parameter affects shape estimates more than size [85].

Trajectory and density parameters could also be used to compare developmental phenotypic plasticity (a term generally used to summarize how a given group responds to different environmental conditions by producing an array of phenotypes [86]). Multivariate reaction norms could be obtained using shape variables but also controlling for other variables (size, external factors) and weighting their interaction. This would enrich the description of phenotypes, while offering a solid basis for comparisons.

As superior organisms, plants have complex shapes that experience complex changes throughout their life spans, particularly when exposed to severe stresses that modify the route of ongoing development. Thus, their complex phenotypes are difficult to encompass to their full extent by using only one technique, regardless of its descriptive or statistical power. This is important when evaluating the capabilities and limitations of the GM tools presented here. For example, we showed that ORMV significantly impacts rosette shape starting at and after 5 DPI (Table 1, Supplementary Table S2). Furthermore, the wireframes (Fig. 5C, 5F, 5I) helped us to detect that some laminae and (almost all) petioles become relatively shorter under ORMV infection. However, no particular statistical statement could be made about these discrete phenotypic outcomes. Rather, if these questions were to be specifically addressed, other measures (such as direct measures of petioles’ length) should have been taken. GM analyses performed here pointed to overall shape (and size) changes. Visualization tools could serve as guides to further study of the putative underlying mechanisms involved, if required. Landmarks analyses come with the limitation of not being capable of extrapolating results to the regions between them without uncertainty. For this reason, the selection of a specific set of landmarks (covering the region of interest) must be well stated at the beginning of the experiment and be sound to study the problem of interest. As with any other technique, caution is needed when interpreting the results because of its limitations. Here, we investigated the contribution of one type of measurement error, the digitization error, which arises from subjective, human error in landmark placement. Other sources of measurement error were not investigated here, such as imaging error (corresponding to the camera) and specimen positioning. We limited our measurement error analysis to the error-prone manual placement of landmarks, but the other types of measurement errors are worth considering. The analysis pipeline is similar and could be performed in MorphoJ or other dedicated software. Moreover, as all biological entities are 3D objects, their approximation to 2D structures inevitably involves some degree of measurement error. This issue has been raised since the first GM studies [87] but has been somehow neglected until recently [88]. Cardini [88] investigated the 2D to 3D approximation and found that shape estimates were quite different for highly 3D structures (crania). In our study, we used a particularly flat rosette, generally considered well suited for 2D approximations [11]. However, with the advancement and lowering costs of 3D imaging and analysis, future studies should benefit from assessing the 2D to 3D approximation over the structures under analysis [88].

After the genomic revolution, there is a need for objective, reproducible, and accurate assessments of plant morphology as a critical missing link to supporting phenomics [89]. In fact, the use of GM tools to analyze plant shape have already started, from a botanical, systematic, archaeological [32, 33, 35], and even experimental [58] point of view.

The use of GM allows the relativization of deviations from controls in a consistent, objective manner. GPA, which is at the core of this conceptual framework and allows us to compare shapes in Procrustes units of distance.

The examples given in this work are necessarily limited, but other applications could be easily envisioned. As the choice of landmarks placement is arbitrary on a given structure, other experimental setups could place them differently in order to study different stages of growth or other anatomical regions of interest. Importantly, this technique is not a competitor but rather a possible complement to newly developed automated platforms for rosette segmentation. Now it is possible for some platforms to identify the tip of leaves, the center of the rosette, and the intersection between lamina and petiole [9, 90]. Ttherefore, the landmarks used in this study and their coordinates could be automatically determined. Moreover, the same software used in this work permits GM 3D image analysis, allowing the study of plant species with a more complex architecture.

One hundred years after the revolutionary vision of D'Arcy Thompson's transformation grids and more than 40 years since the beginning of the revolution in morphometrics, GM application for plant phenotyping is starting to develop [34, 35, 91]. Thus, the research on the plant model species *A. thaliana* should benefit from it.

## Availability of supporting data

Datasets supporting the results of this article are available via the *GigaScience* GigaDB repository [92].

## Supplementary Material

GIGA-D-17-00284_Original_Submission.pdf

GIGA-D-17-00284_Original_submission_Reviewer1_attachment.pdf

GIGA-D-17-00284_Revision_1.pdf

GIGA-D-17-00284_Revision_2.pdf

Response_to_Reviewer_Comments_Original_Submission.pdf

Response_to_Reviewer_Comments_Revision_1.pdf

Reviewer_1_Report_(Original_Submission) -- Andrea Cardini 12/06/2017 Reviewed

Reviewer_2_Report_(Original_Submission) -- Yoland Savriama 12/13/2017 Reviewed

Reviewer_3_Report_(Revision_1) -- Tim Dickinson 03/26/2018 Reviewed

Supplement Files

## Additional files

**Supplementary Figure S1**. Graphical assessment of the Tangent shape space approximation. Scatterplots of distances in the tangent space against Procrustes distances (geodesic distances in radians) for (A) Mock-inoculated plants, (B) ORMV-infected plants and (C) all plants, over all DPIs. A blue line is plotted to show a slope of 1 through the origin. Then a least-squares regression line through the origin is shown in red (for data in which the variation in shape is small this will hide the blue line).

**Supplementary Figure S2**. Wireframes depicting shape change associated with –PC1 values from 3 to 12 DPI (A-H). Green = starting (average) shape; red = target shape. No magnification was applied.

Supplementary File ORMV

Supplementary File TuMV 1st

Supplementary File TuMV 2nd

**Supplementary Table S1**. Summary statistics for the comparisons between Tangent (Euclidean) and Procrustes shape distances from average shapes and for regression slopes and correlations between the two distances.

**Supplementary Table S2**. Summary of centroid size and shape variation. Hierarchical sum of squares ANOVA. Main effect: Treatments; random factors: Individuals (ID), Digitization. SS, MS and df refer respectively to sum of squares, mean sum of squares (i.e., SS divided by df) and degrees of freedom. Error1 = digitization error.

**Supplementary Table S3**. Statistical comparisons of intra-treatment shape changes across the ORMV experiment. Holm’s-Bonferroni sequential correction at α = 0.05.

**Supplementary Table S4**. Classification/misclassification tables from DA for each DPI and human observers for 7 DPI.

**Supplementary Table S5**. Results of allometry tests for each treatment and DPI (top) and of the regression analyses (MANCOVA), for testing differences between slopes when allometry was detected (bottom).

**Supplementary Table S6**. Discriminant Analysis for TuMV. Statistical tests for differences between means of treatments at each DPI from DA (with permutation tests with 1000 random runs) and classification/misclassification tables for each DPI.

## Abbreviations

2D: two dimensional; 3D: three dimensional; ANOVA: analysis of variance; CS: centroid size; DA: discriminant anaylsis; DPI: days post-inoculation; GM: geometric morphometrics; GPA: generalized Procrustes analysis; MANCOVA: multivariate analysis of covariance; MANOVA: multivariate analysis of variance; MPL: morphometric path length; ORMV: oilseed rape mosaic virus; PC: principal component; PCA: Principal Component Analysis; PTA: phenotypic trajectory analyses; SS: sum of squares; TuMV-UK1: turnip mosaic virus-UK1 strain.
