# Supplementary material for: Arabidopsis phenotyping through geometric morphometrics
Source: Gigascience. 2018 Jun 18;7(7):giy073. doi: 10.1093/gigascience/giy073 (PMC6041757; doi:10.1093/gigascience/giy073)
Supplement: GIGA-D-17-00284_Revision_2.pdf [file giy073_giga-d-17-00284_revision_2.pdf]

# GigaScience
## Arabidopsis phenotyping through Geometric Morphometrics
--Manuscript Draft--

| | |
|---|---|
| **Manuscript Number:** | GIGA-D-17-00284R2 |
| **Full Title:** | Arabidopsis phenotyping through Geometric Morphometrics |
| **Article Type:** | Research |
| **Funding Information:** | Fondo para la Investigación Científica y Tecnológica (PICT 2014-1163) — Dr Sebastian Asurmendi<br>Instituto Nacional de Tecnología Agropecuaria (INTA) (PE 11310022) — Dr Sebastian Asurmendi |
| **Abstract:** | Recently, much technical progress was done regarding plant phenotyping. High-throughput platforms and the development of improved algorithms for the rosette image segmentation make now possible to massively extract shape and size parameters for genetic, physiological and environmental studies. The development of low-cost phenotyping platforms and freeware resources make it possible to widely expand phenotypic analysis tools for Arabidopsis. However, objective descriptors of shape parameters that could be used independently of platform and segmentation software used are still lacking and shape descriptions still rely on ad hoc or even sometimes contradictory descriptors, which could make comparisons difficult and perhaps inaccurate. Modern geometric morphometrics is a family of methods in quantitative biology proposed to be the main source of data and analytical tools in the emerging field of phenomics studies. Based on the location of landmarks (corresponding points) over imaged specimens and by combining geometry, multivariate analysis and powerful statistical techniques, these tools offer the possibility to reproducibly and accurately account for shape variations amongst groups and measure them in shape distance units. Here, it is proposed a particular scheme of landmarks placement on Arabidopsis rosette images to study shape variation in the case of viral infection processes. Shape differences between controls and infected plants are quantified throughout the infectious process and visualized. Quantitative comparisons between two unrelated ssRNA+ viruses are shown and reproducibility issues are assessed. Combined with the newest automated platforms and plant segmentation procedures, geometric morphometric tools could boost phenotypic features extraction and processing in an objective, reproducible manner. |
| **Corresponding Author:** | Sebastian Asurmendi<br>Instituto Nacional de Tecnologia Agropecuaria<br>Hurlingham, Buenos Aires ARGENTINA |
| **Corresponding Author Secondary Information:** | |
| **Corresponding Author's Institution:** | Instituto Nacional de Tecnologia Agropecuaria |
| **Corresponding Author's Secondary Institution:** | |
| **First Author:** | Carlos Augusto Manacorda |
| **First Author Secondary Information:** | |
| **Order of Authors:** | Carlos Augusto Manacorda<br>Sebastian Asurmendi |
| **Order of Authors Secondary Information:** | |
| **Response to Reviewers:** | Dear Dr. Hans Zauner,<br><br>We are pleased to resubmit the paper “Arabidopsis phenotyping through Geometric Morphometrics” revision 2 .<br>We believe that the manuscript has been considerably improved and we hope it will |

Powered by Editorial Manager® and ProduXion Manager® from Aries Systems Corporation

were able to meet the requirements to be considered for publication in GIGAScience.

Yours sincerely,

Carlos A. Manacorda and Sebastián Asurmendi

Global response

Our aim in this work was to apply GM tools to address the general question of whether differences in rosette shape exist between two experimental conditions applied to Arabidopsis in an experimental context. This purpose aimed be able to statistically quantify the shapes differences between treatments in order to establish objective global comparison on a matter that is usually in some way subjective, as virus phenotype severity for example. Taking individual measurements (angles, petioles and/or laminae lengths, etc.), could indeed give valuable information, but the comparative analysis of shape as a whole needs the application of GM tools, whose mathematical approach allows to simultaneously consider the relative displacements of all selected landmarks taken together. By doing so, a single measure (Procrustes distance) gives the morphometric distance from one shape to the other one (the reference, for example, that could be the mean shape of control plants). Classic morphometric measures have their own problems. For example, it is indeed possible, using the same set of landmarks used here and the same software (TPSDig2), to take measurement distances from the center of the rosette to the end of one petiole, from that point to the tip of the leaf under study and from the center to the tip. However, when several measurements radiate from a single point their values cannot be completely independent; any error in locating that point affects all of these measurements. Such a data set contains less information than could have been collected with no greater effort because some directions are measured redundantly and many measurements overlap (Zelditch, 2012). Similar problems could arise if we had measured successive angles between leaves, as Tim Dickinson properly note (circularity of the data), and this is precisely what we had not done.

Particular responses

Although we do not agree with Dr. Dickinson´s statement that "The authors neglect to demonstrate a problem that their method solves with reference to actual plants." we have done an effort to clarify the understanding of this issue based on the reviewer comments.
We stated in the original manuscript (Page 3, last paragraph) that "This work aims to introduce the use of GM tools for the analysis of Arabidopsis rosette phenotypes in an objective and repeatable way. Viral infections are used as case studies to exemplify the potentially usefulness of these techniques to quantitatively reveal shape changes using this plant model".
We changed to
“This work aims to introduce the use of GM tools for the analysis of the Arabidopsis rosette. Its purpose is to statistically quantify the shape differences between treatments in order to establish objective global comparisons on a matter that is usually subjective, virus phenotype severity. Viral infections are used as case studies to exemplify the potentially usefulness of these techniques to quantitatively reveal shape changes using this plant model. As such, it is not intended to offer a complete introductory explanation of each GM tool, an objective that is beyond the scope of this paper. Such a task was already performed by [33] and for a complete introductory explanation of GM tools applied in biological systems it is recommended the lecture of [41]. Software used in this work frequently has its own user´s manual and informative examples [42–44]. Nevertheless, with the purpose to facilitate the comprehension of this work to newcomers in the field of GM, each tool is briefly described in the Methods section.”

We used actual plants, throughout the study, to demonstrate that viral infections not only affect rosettes´ size (Fig. 2) but also their shape (Figs. 4 to 9 and associated Tables and Suppl. Tables). This effect should not be taken for granted prior to size and shape separation by GM analyses (one cannot exclude a priori a “freezing effect” of the virus that affected size but not shape). Secondly, by performing three independent experiments, we found that the rossette shape is more severely impacted by TuMV than ORMV (Fig. 9A vs. B, D vs. F, and Table 1 vs. Suppl. Table 6). Again, this effect

is by no means obvious prior to analyses. The comparison of phenotypic trajectories (PTA, Fig. 9A and B and the associated Table 2) quantified the difference and allowed us to find that, based on trajectory divergence angles and trajectory shape differences TuMV is a more severe virus regarding Arabidopsis rosette shape along the infection time frame. Of course more subtle problems could be addressed using these tools, but this initial work aims to illustrate the capabilities and potential of GM in the field.

Dr. Dickinson stated that “As I understand it, the authors seek to automate the comparison of rosettes so as to analyze the effect of the virus, and perhaps also detect plants in which virus symptoms have appeared”. Whereas he is right in saying that we are interested in the analyses of virus effect and the potential to detect infections by image, regarding automation we only suggested in the Discussion that GM tools could be added to existing software packages (or used to develop newly, dedicated ones), but we performed our landmarks assignation manually here, i. e., without any implementation of automation at this step (we stated it at the M&M section). As mentioned previously the main objective was to establish a quantitative measurement of global shape differences in Arabidopsis that could be automated later on.

Dr. Dickinson follows stating that: “If this really is their objective then I think they need more than the existing Fig. 1 in this ms to demonstrate what they are attempting to do. If the authors' Fig. 1 is revised to meet this concern, then readers can evaluate for themselves whether the authors have succeeded with the great lengths to which they have gone to employ geometric morphometric methods to detect changes in divergence angle between leaves in a rosette, and changes in petiole and blade lengths.”, we have deposited at journal database all images datasets for all three independent experiments at each DPI, along with the .TPS and .morphoJ files that are the input and output of the main analyses and upon which the main conclusion of this work is based on. The interested reader would be fully capable not only to visually inspect every rosette but also to re-run the entire analysis. Fig. 1 only depicts a representative rosette at 8 DPI, with illustrative aims. We modified a sentence in Fig. 1 caption to further clarify this point: Removed: Landmark configuration in an Arabidopsis rosette. Introduced: Landmark configuration in a representative Arabidopsis rosette. We also slightly modified the sentence introducing Fig. 1 in the main text to make it clearer: (Removed: An 11-landmark configuration for the Arabidopsis rosette is shown in Figure 1A. Introduced : An 11-landmark configuration for a representative Arabidopsis rosette is shown in Figure 1A.).
For the demonstration that our approach here is capable of detecting infected plants, we have done DA analyses (Fig. 5, Suppl. Table 4 including comparison with Human Observers). Again, the interested reader can download the Dataset at every DPI to assign treatments visually and compare her results with those obtained here applying GM tools. With respect to the last observation made by Dr. Dickinson here about divergence angles and petioles and blade lengths, we want again to emphasize that all differences found are relative to the whole shape change of the structure under consideration here (i.e., the rosette as spatially defined by the landmarks we positioned). We did not state that any structure is more or less lengthy, for example, in absolute terms, but in relative ones (relative to the shape change of the whole configuration). Nonetheless, we think that Dr. Dickinson raises an interesting question here that points out to the possible origin of the (relatively) distorted inter-leaves angles and how to measure them.

Because of that, we introduced a short paragraph in the Discussion to further clarify this issue (“It is important to note here that we did not perform absolute individual angle or distances measurements. This kind of traditional morphometric measurements could also be manually taken by using TPSDig2 software and would be an addition to the parameters presented here, where the focus is only on geometric morphometric tools. However, the normal leaf phyllotactic pattern (the arrangement of organs in regular patterns around the stem of a plant) seems not to be changed by these viruses (personal observations, data not shown). Instead, the distortion of the angle determined by the tip of two successive leaves (with its vertex in the center of the plant) appears to arise from the relative outgrowth of the distal part of the lamina”). Below we added a brief paragraph also stating that “In this particular study, the observance of the relative shortening of petioles or laminae, and the distorted inter-leaves angles could lead to further experiments to more precisely quantify these discrete phenotypes using also traditional morphometric measurements. These

| | |
|---|---|
| | traditional measurements however should be carried on taking into account its intrinsic statistic limitations (see [41], Introduction).” We rearranged this section of the Discussion hoping the reader could better understand our point here.<br><br>As for the Editor´s comment on the exact meaning of “image segmentation”, we clarified further what we meant, trying to aid the reader coming from other fields to fully understand our point here. In the Background section We Removed: “Freely available software that overcome the difficult task of rosette segmentation (an issue still under investigation) have been developed by different means (…)” and Introduced: “Freely available software that overcome the difficult task of image rosette segmentation (i. e. the computational separation of the living plant tissue from the substrate background) is an issue still under investigation and have been developed by different means (…)”.<br><br>We also had our ms. revised and modified by a specialized person to improve its language and grammar.<br><br>We therefore hope we had clarified the points raised both by the Editor and Reviewer #3. We nonetheless remain open to further suggestions to improve our Manuscript. |
| **Additional Information:** | |
| **Question** | **Response** |
| Are you submitting this manuscript to a special series or article collection? | No |
| **Experimental design and statistics**<br><br>Full details of the experimental design and statistical methods used should be given in the Methods section, as detailed in our Minimum Standards Reporting Checklist. Information essential to interpreting the data presented should be made available in the figure legends.<br><br>Have you included all the information requested in your manuscript? | Yes |
| **Resources**<br><br>A description of all resources used, including antibodies, cell lines, animals and software tools, with enough information to allow them to be uniquely identified, should be included in the Methods section. Authors are strongly encouraged to cite Research Resource Identifiers (RRIDs) for antibodies, model organisms and tools, where possible.<br><br>Have you included the information requested as detailed in our Minimum Standards Reporting Checklist? | Yes |
| **Availability of data and materials**<br><br>All datasets and code on which the | Yes |

| | |
|---|---|
| conclusions of the paper rely must be either included in your submission or deposited in publicly available repositories (where available and ethically appropriate), referencing such data using a unique identifier in the references and in the "Availability of Data and Materials" section of your manuscript.<br><br>Have you have met the above requirement as detailed in our Minimum Standards Reporting Checklist? | |

Powered by Editorial Manager® and ProduXion Manager® from Aries Systems Corporation

Manuscript

Click here to access/download;Manuscript;Manacorda et al.R2_8_8_18.docx

Click here to view linked References

# Arabidopsis phenotyping through Geometric Morphometrics

Carlos A. Manacorda[1] and Sebastian Asurmendi [1,2].

[1] Instituto de Biotecnología, CICVyA, INTA, Argentina [2] CONICET, Argentina.

## Abstract

**Background:** Recently, much technical progress was achieved in the field of plant phenotyping. High-throughput platforms and the development of improved algorithms for rosette image segmentation make it now possible to extract shape and size parameters for genetic, physiological and environmental studies on a large scale. The development of low-cost phenotyping platforms and freeware resources make it possible to widely expand phenotypic analysis tools for Arabidopsis. However, objective descriptors of shape parameters that could be used independently of platform and segmentation software used are still lacking and shape descriptions still rely on *ad hoc* or even sometimes contradictory descriptors, which could make comparisons difficult and perhaps inaccurate. Modern geometric morphometrics is a family of methods in quantitative biology proposed to be the main source of data and analytical tools in the emerging field of phenomics studies. Based on the location of landmarks (corresponding points) over imaged specimens and by combining geometry, multivariate analysis and powerful statistical techniques, these tools offer the possibility to reproducibly and accurately account for shape variations amongst groups and measure them in shape distance units.

**Results:** Here, a particular scheme of landmarks placement on Arabidopsis rosette images is proposed to study shape variation in the case of viral infection processes. Shape differences between controls and infected plants are quantified throughout the infectious process and visualized. Quantitative comparisons between two unrelated ssRNA+ viruses are shown and reproducibility issues are assessed.

**Conclusion:** Combined with the newest automated platforms and plant segmentation procedures, geometric morphometric tools could boost phenotypic features extraction and processing in an objective, reproducible manner.

## Background

Plant phenotyping is the process of recording quantitative and qualitative plant traits and is essential to study plant responses to the environment [1]. A survey of the International Plant Phenotyping Network (IPPN)[2] among plant scientists found that most participants think that plant phenotyping will play an important role in the future. The selected topics of main interest were the assessment of abiotic, biotic and multiple stress and the model plant *Arabidopsis thaliana*. Recently, many new techniques have been developed to facilitate and improve quantitative plant phenomics (i.e. the full set of phenotypic features of an individual), going from destructive to non-destructive and even high-throughput phenotyping (the use of cameras and automated platforms to automatically extract phenotypic features on hundreds of plants per day) [3–5]. Whereas the throughput is an important aspect of phenotyping, spatial and temporal resolutions, as well as accuracy, should be considered [6].

Freely available software that overcome the difficult task of image rosette segmentation (i. e. the computational separation of the living plant tissue from the substrate background) is an issue still under investigation and have been developed by different means [7–11]. These software packages allow the assessment of several rosette parameters such as area, perimeter and other more complex descriptors. However, the persistence of *ad hoc* descriptors [12,13] and lack of a gold standard could give rise to reproducibility issues, because of different growing substrate-segmentation algorithm combinations. Moreover, different approaches sometimes give the same name to different parameters (e.g. “roundness” in ImageJ [14] vs. [10]) or different names to the same parameter (e.g. “solidity” in [11] equals “compactness” in [7,10] and “surface coverage” in [5]). The need of developing objective, mathematically and statistically sound and more accurate shape descriptors in plants has been stressed out in recent reviews on the topic [15–17]. Nonetheless, image datasets analyses require a conceptual and statistical corpus of knowledge that is not always present in a plant biologist’s research field. Plant phenotyping relies on skills and technologies that are used to characterize qualitative or quantitative traits regardless of the throughput of the analyses [1]. One such knowledge corpus is morphometrics [18].

Traditional morphometric analyses such as measures and ratios of length, depth and width were widely used in evolutionary biology, taxonomy etc. studies throughout the 20th century. To the end of that century the seminal work of Thompson [19] was re-evaluated under the light of multivariate analysis and novel mathematical developments [20,21], giving rise to modern geometric morphometrics (GM), in which was called a “revolution” in morphometrics [22–24].

GM combines geometry, multivariate morphometrics, computer science and imaging techniques for a powerful and accurate study of organismal forms. This family of methods in quantitative biology is proposed to be the main source of data and analytical tools in the emerging field of phenomics [25]. Formally, GM is “a collection

of approaches for the multivariate statistical analysis of Cartesian coordinate data, usually (but not always) limited to landmark point locations" [26] . Landmark methods have been successfully applied to various species, and have the advantage of being easy to understand [27]. Besides enhanced statistical power and better descriptive and graphical tools, GM allow researchers to decompose form in size and shape, and the whole configuration of the organism under study is analyzed, rather relying on the description of relative displacements of pairs of points. GM is now a mature discipline that has been widely applied in biology [28–30] (see [31] for a review). For example in plants, seeds of barley [32] and leaves of grapevine [33] and oak [34,35] were studied using GM methods.

Plant viruses cause important worldwide economic losses in crops [36]. Symptoms include plant stunting, changes in leaf morphology, and sometimes plant death [37] and vary depending on various aspects including genetic compatibility and environmental conditions. Even given a particular host-virus interaction, different viral strains trigger different symptomatology, which are more or less subtle for the observer to distinguish [38–40]. Comparing the severity of qualitative viral symptoms (i.e. the degree to which infected plants depart from healthy controls in some observable phenotype, often referred to aerial parts of the plant like leaves, stems or rosettes) is a difficult task performed mainly by visually rating symptoms (e.g. [41]). Consequently, morphological differences could be difficult to describe and reproducibility issues could arise.

*Arabidopsis thaliana*. has been extensively used in studies of influences of environmental factors on plants, paving the path to the development and testing of experimental techniques or data analysis methods [42]. The Arabidopsis rosette is a nearly two-dimensional (2D) structure in the vegetative phase [11], thereby facilitating image acquisition and interpretation. Here, it is proposed a case study where GM tools are applied to study and quantitatively describe morphometric changes triggered in *A. thaliana* plants by RNA viruses belonging to two unrelated families. It is proposed a particular selection of landmarks to locate in the Arabidopsis rosette during its vegetative phase. The study spans from the earlier stages of viral infection to later periods, when symptoms are detectable with the naked eye. Comparisons are made between discriminant power of computer-assisted classification and expert human eye. Symptoms severity triggered by both viruses is also compared, based on the relative morphometric changes induced relative to healthy controls. Changes in allometric growth, phenotypic trajectories and morphospace occupation patterns are also investigated. Size analyses are also performed. Throughout this work, several bioinformatics resources are applied, in order both to extract the higher degree of information available, but also to exemplify different and complementary possibilities that nowadays GM offers for the accurate description of shape in Arabidopsis.

This work aims to introduce the use of GM tools for the analysis of the Arabidopsis rosette. Its purpose is to statistically quantify the shape differences between treatments in order to establish objective global comparisons on a matter that is usually subjective, virus phenotype severity. Viral infections are used as case

studies to exemplify the potentially usefulness of these techniques to quantitatively reveal shape changes using this plant model. As such, it is not intended to offer a complete introductory explanation of each GM tool, an objective that is beyond the scope of this paper. Such a task was already performed by [35] and for a complete introductory explanation of GM tools applied in biological systems it is recommended the lecture of [43]. Software used in this work frequently has its own user´s manual and informative examples [44–46]. Nevertheless, with the purpose to facilitate the comprehension of this work to newcomers in the field of GM, each tool is briefly described in the Methods section.

## Materials and Methods

### Plant growth conditions

*A. thaliana* Col-0 seeds were stratified at 4°C for 3 days. Plants were grown under short days conditions (10 h light/14 h dark cycle, T(°C)= 23/21, Hr(%)= 60/65, and a light intensity of 150 μE m-2 s-1) in a controlled environmental chamber (Conviron PGR14; Conviron, Winnipeg, Manitoba, Canada). Plants were grown in individual pots in trays and treatments were assigned to plants in all trays. One experiment was performed with ORMV and two independent experiments were carried on with TuMV-UK1.

### Virus infection assays

ORMV (Oilseed Rape Mosaic Virus) [47] was maintained in *Nicotiana tabacum* (cv. Xhanti nn) and infective sap was obtained after grinding infected leaves with mortar and pestle in 50 mM phosphate buffer pH=7.5. TuMV (Turnip Mosaic Virus)-UK1 strain (accession number X65978) [48] was maintained in infected *A. thaliana* Col-0. Fresh sap was obtained immediately prior to use to inoculate plants with sodium sulfite buffer (1% $K_2HPO_4$ + 0,1% $Na_2SO_3$ [wt/vol]). Mock-inoculated plants were rubbed with carborundum dust with either 50 mM phosphate buffer pH=7.5 or sodium sulfite buffer, respectively. Plants were mechanically inoculated in their third true leaf at stage 1.08 at 21 days post-sowing, [49] because those leaves were almost fully developed by the time of the procedure and therefore constituted a source tissue for the export of virions to the rest of the plant. The number of plants assigned to each treatment in each experiment is detailed in Table 2.

### Image acquisition

Zenithal photographs of individual plants growing in pots were taken with a Canon PowerShot SX50HS camera mounted in a monopod at maximal resolution. Specimens were imaged at different Days Post-Inoculation (DPI) spanning from 3 to 12 DPI. Photographs were taken at the same time of the day in successive days to minimize error. A ruler was placed next to each plant at each image acquisition and only its central part (60-80 mm) was taken into account to avoid image distortion at the edges of the photograph [50].

### Landmarks configuration and digitization

At the heart of GM analyses is the concept of landmarks. Landmarks are points that can be located precisely over a structure and correspond in a one-to-one manner among all the specimens included in a study [51]. There is no absolute landmark configuration on any given structure. The choice of the number of landmarks and their configuration depend on the hypotheses being tested [52]. Here, the focus of the analyses is on the phenotypic impact of viral infections on the Arabidopsis rosette through time. Hence, short-day conditions were chosen to maintain the vegetative phase and delay flowering (when stems and reproductive organs could mask morphological features of leaves in zenithal photographs), allowing the plant´s aerial part to remain near two-dimensional during the experiment. Landmarks were chosen based in part due to its relatively easiness of recognition in an Arabidopsis rosette in vegetative phase and, to encompass as broadly as possible the phenotypic changes experienced by the plant during the infection, chosen landmarks were present from earlier stages of the infection to later ones and placed in regions that experience dramatic changes upon infection [39]. Also, selected landmarks could take into account relevant morphological changes induced by stresses or distinctive phenotypes of different ecotypes like relative shortening or lengthening of petioles and laminae, or relative lateral displacements of leaves [7,10,11]. Moreover, landmarks chosen here are probably less prone to manual digitization error than, for example, a landmark situated in the middle of the laminae or placed somewhere along the leaf´s contour, a task that seems rather complicated given the serrated nature of Arabidopsis leaves and the fact that the degree and placement of serration changes along the development of successive leaves [53–55]. A relatively reduced number of landmarks can be used to describe complex forms [35,56].

The selection of landmarks is based also in the observance of five basic principles, including the basic requirements for 2D approximation [43]:

1) Homology (in the sense of correspondence of points). The points on one specimen correspond (as the “same” point) to that point on all individuals.
2) Adequate Coverage of the Form (or comprehensive coverage). Landmarks should be chosen in a way they encompass the structure over which the biological hypothesis of interest is being tested and are functional to the specific aim of a study.
3) Repeatability. The same landmarks should be easily identified in the same structure in order to reduce digitization error (a component of measurement error).
4) Consistency of Relative Position. This attribute guarantees that landmarks do not interchange relative positions.
5) Coplanarity. When digitizing real, 3D structures under the 2D approximation, landmarks should be placed as close as possible onto an imaginary plane to reduce the distortion associated with that approximation.

The TPSUtil software (a member of the TPS Series of GM tools [46] that prepares the data for further analyses) was used to create .TXT files with a .TPS extension from the directories containing the .JPG images. These were used to load the .JPG images in TPSDig2. Opening these .TPS files with TPSDig2 allows the user to proceed with the digitization of landmarks. An 11-landmark configuration for the Arabidopsis rosette is shown in Figure 1A. The 11 landmarks were digitized in the same order on each picture, after setting a scale factor with a ruler, at each DPI. This scale factor is set in TPSDig2 selecting two points placed at a known distance between them in the photograph and allows for the correction of possible differences in distances from the camera objective to the specimen under study (from one day to the next, for instance). The scale factor is important to measure centroid size, amongst other possible measures, but has no effect on landmark coordinates, which remain in pixels units [46].

Following Bookstein's criteria [57], landmark 11 (which is situated at the centre of the rosette) is a Type 1 landmark because it is the intersection of the base of all petioles, i.e., its juxtaposition and hence very locally defined. Type 1 landmarks are frequently considered as optimal [43,57]. Landmarks 1, 2, 3, 4 and 5 (which are located at the tip of leaves #8 to #12 and are the maxima of curvature of that structure) and landmarks 6, 7, 8, 9 and 10 (which are located at the intersection of the petiole and the lamina of each leaf from #8 to #12) cannot be unambiguously assigned due to the continuous nature of the leaf curvature and are Type 2 landmarks. Leaves below number 8 were not chosen for landmark placement for three main reasons: a) they are hidden for younger leaves at later stages of infection b) these old leaves had almost finished their growth by the time the first photographs were taken (and the form covered by them would be a less informative one for the process of shape and size change upon viral infection) and c) the senescence process of older leaves lead to morphological changes derived from dehydration and death. Younger leaves (beyond leaf number 12) were not chosen because they were not present at the earlier stages of infections, therefore violating the requisite of repeatability of landmarks.

Average specimen digitization time was about 1 minute. The Output of TPSDig2 is a .TPS file containing information about specimen name, scale factor, and raw coordinates of each landmark for all digitized specimens. Landmark digitization was repeated a week after to estimate the digitization component of the measurement error for each specimen.

**Workflow**

A flowchart of data analyses throughout this paper is shown in Figure 1B. Image datasets for all DPIs and both treatments were handled and digitized for further analyses using the TPSUtil and TPSDig2 software packages that generate .TPS output files. Several freeware can be used to extract shape information from .TPS files [43]. Here, the MorphoJ software [44] was chosen mainly because of its ease to use and comprehensive tools available. MorphoJ creates new datasets from several file extensions, including .TPS. The "Supplementary file

ORMV.morphoj" was created and 16 datasets were generated, one for each DPI and digitization instance. Specimens were classified according with ID, Treatment, DPI and Digitization for each dataset. Combinations of classifiers were also done to perform further grouped analyses. Other complimentary analyses and shape change visualizations were performed by exporting output files from MorphoJ to other software packages. TuMV analyses were done in the same way.

**Procrustes fit and outlier detection**

The first step of shape analysis in GM consists in extracting shape coordinates from raw data obtained at the digitization step. The standard procedure in GM studies is the Generalized Procrustes Analysis (GPA). The purpose of Procrustes procedures is to remove from the specimens all information that is not relevant for shape comparisons, including size. Specimens are firstly translated at the origin ("superimposed") by subtracting the coordinates of its centroid from the corresponding (X or Y) coordinates of each landmark. Then, differences in size are removed by rescaling each specimen to the mean centroid size (CS) (CS is calculated as the square root of the summed squared distances of each landmark from the centroid). Differences in rotation are eliminated by rotating specimens, minimizing the summed squared distances between homologous landmarks (over all landmarks) between the shapes. MorphoJ performs a full Procrustes fit, which is a variant of the analysis that is more conservative and resistant to outliers of shape.

A few recent studies, focusing on flowers [58–60] or leaves [61], study asymmetry in plants by using geometric morphometrics, compared with those in animals [51]. Although this work will not study the asymmetry issue, it must be briefly considered. In Arabidopsis, the arrangement of organs along the stem (phyllotaxy) follows a predictable pattern, the Fibonacci series. Phyllotaxy orientation can be clockwise or counter-clockwise [62]. There is no preferred orientation of Arabidopsis rosettes and in this study 20 were clockwise, whereas 21 were counter-clockwise. It is thus an example of antisymmetry, where (following Klingenberg´s [51] explanation) "most individuals are asymmetric, but differ in the directions of the asymmetries so that there is a mix of "left-sided" and "right-sided" individuals". This creates a bimodal distribution that should be considered because clockwise and counter-clockwise rosettes are biological enantiomorphs and must not be directly superimposed by GPA. Opportunely, MorphoJ automatically performs reflections on every specimen when executing a GPA and therefore it is not a problem at this stage. However, care must be taken with different software. Alternatively, rosettes can be reflected using TPSDig2 to leave all clockwise or counter-clockwise rosettes prior to landmark digitization.

Although the full Procrustes fit performed by MorphoJ is considered to be more resistant to outliers of shape [44], there could still be specimens that divert from the rest to a great extent. The "Find Outliers" option in the "Preliminaries" menu provides an indication of how unusual an individual is relative to the others in the sample (using Mahalanobis distance in larger samples). The user can therefore consider subtracting this specimen from

the rest as an outlier. A GPA was run for each dataset (each comprising one of the DPIs and one digitization replicate) and outliers were evaluated in each of the 16 datasets, separately.

**Assessment of the tangent space approximation**

For a given M-dimensional structure with K landmarks (here, M = 2 and K = 11) an individual´s shape can be visualized as a point in an M X K multidimensional space (a hypersphere). After centering and rescaling, three dimensions are lost and shapes are said to be in a pre-shape space; they are not rotated yet. The distance in the hypersphere surface at which rotation differences between shapes are minimal, is called Procrustes distance (the conventional measure of a morphometric distance in geometric morphometrics [63]). Afterwards, a reference (average) shape is selected and all other shapes are rotated to minimize distances relative to it, generating a shape space and losing one more dimension (remaining 2K-4). Because distances over curved multidimensional spaces are non-Euclidean, conventional tools of statistical inference cannot be used. Fortunately, for most biological shapes a good approximation to Euclidean distances is possible, by projecting shape points to a tangent Euclidean space (for a visual explanation see [43]). This assumption should, however, be tested when analyzing new data. TPSSmall is used to determine whether the amount of variation in shape in a data set is small enough to perform statistical analyses in the linear tangent space approximate to Kendall's shape space which is non-linear. This task is performed by basically comparing the Procrustes distances obtained using both shape spaces. Since TPSSmall does not perform reflections, datasets analyzed with TPSDig2 were opened again and specimens reflected when necessary to leave all clockwise rosettes. Two data subsets were created for each DPI, one with Mock-inoculated plants and other with ORMV-infected plants. Next, the datasets were combined across DPIs using the “Append files” option of TPSUtil to create three main datasets (Mock, ORMV and ALL plants).

**Testing digitization error and variation between treatments using Procrustes ANOVA**

As mentioned before, two digitization instances were performed on each plant at each DPI, in order to evaluate digitization error. This procedure is important because digitization error should always account for far less variance in the subsequent analyses than specimens and treatments do [35]. The differences between the samples and particularly between the treatments are the ones worth investigating, not the human error in landmark placement. Purposely, datasets for each DPI were combined and subjected to a hierarchical analysis of variance (ANOVA). In MorphoJ this is a Procrustes ANOVA, with “Treatment” as an additional main effect, “ID” for the individuals and “Digitization” as the Error1 source (the last term is equivalent to the Residuals here, as only one source of error is being quantified). One Procrustes ANOVA was performed for each DPI, separately.

In Procrustes ANOVA, variance is partitioned by means of hierarchical sum of squares (SS) which implies that each effect is adjusted for effects that appear earlier in the hierarchy. This takes into account the nested

structure of the data and therefore allows the quantification of differences in Treatments and individuals (plants) regardless of Treatment. (This is an issue that is crucial if the design is unbalanced,that is, with unequal sample sizes, in part because hypothesis tests are more robust to the assumptions of normality and equal variance when the design is balanced. Although the design here is unbalanced (24 Mock and 17 ORMV plants), it is considered a minor problem for designs that are not extremely unbalanced and/or do not involve more than one factor [43,64]. It should, however, be taken into account when multiple factors are studied, requiring special calculations for obtaining the correct sum of squares. MorphoJ also recommends to use data that are as balanced as possible (see [43] for a discussion on the topic). The variance unexplained by any of these effects (Treatment and Individual) is digitization error and it is estimated using the differences between digitizations. Hence, total variance was decomposed into main (treatment) and random (ID and digitization) components and was expressed as a percentage of total variance for each DPI. Statistical significance is provided by Goodall´s F-tests [65], for size and shape. The parametric Goodall´s F-test assumes isotropic variation (the assumption that there is an equal amount of variation around each landmark), which is often violated in biological studies [66]. For many practical applications it is possible to use the approach based on Procrustes distances to assess the relative magnitudes of effects, but to make statistical inferences, the MANOVA approach is used [44]. For this reason, MorphoJ includes a multivariate test (Pillai´s Trace) for shape.

**Ordination Methods and shape change visualization**

***PCA***

Once shape variables (the 22 Procrustes Coordinates) are extracted for all specimens at each DPI, it is a useful option to plot differences between individuals and treatments. However, patterns of variation and covariation between lots of variables are difficult to interpret and shape variables are not statistically independent [43]. PCA is a technique that simplifies those patterns and therefore makes them easier to interpret. When a PCA is performed, the original, possible correlated set of shape variables are mathematically transformed to create a new set of orthogonal and independent variables (principal components, PCs) that are a linear combination of the original variables. PCs do not covary, but carry all the original shape information. As each PC explains sequentially less shape variance, this approach is often used to restrict the analysis to the first few PCs that account for most of total variance [43]. However, some recent developments in the GM field [67] propose that PCA should be at least carefully interpreted, since the biological meaning of the PC axes are difficult to assess. Here, PCA will be used conservatively to discuss relative shape distances between individuals belonging to different groups, as advised by Howells [68]. It is also important to remember that PCA is useful for studying distances between individuals, not groups, and although a powerful descriptive tool, it does not involve any statistical test. Therefore, the relative separation of groups in a PCA plot does not allow one to draw conclusions about significant differences (or its absence).

***Visualization of shape changes***

A brief description of common GM visualization tools is needed in order to accurately interpret the results. After the GPA, every configuration in the sample is optimally aligned to the average configuration and nearly optimally aligned to every other configuration in the sample [69]. GPA has already removed differences attributable to size, position and orientation from configurations. All differences that remain are shape variation. Accordingly, shape differences are found using the relative displacements of the landmarks from one shape to another nearby in shape space [69].

A key concept to bear in mind is that it is fundamentally wrong to consider landmarks displacements in an isolated manner [43,69] (see example in [35]). This is because all the landmarks in the GPA jointly determine the alignment of each configuration in relation to the mean shape. Then, the variation in the position of each landmark after superimposition is relative to the positions of all other landmarks. Although a shift is shown at every landmark, these shifts are relative to all other landmarks. Lollipop and wireframe graphs are based on these assumptions (see the Results section).

Shape variation could be depicted by means of transformation grids, which are mathematically constructed following the thin-plate spline technique [43,57,69]. Briefly, landmarks of a starting shape are placed on a grid of an imaginary infinitely thin metal plate. Landmarks of a target configuration are placed on another grid of equal characteristics, and both metal sheets are superimposed. Each landmark in the starting shape (e.g., mean shape) is linked to its homologous to reach the target configuration, and the deformation caused in the spline is calculated finding the smoothest interpolating function that estimates energy changes in the spline between landmarks. Importantly, unlike lollipop or wireframe graphs, transformation grids distribute the change in landmark positions to the space between landmarks, were no objective information is available. Then, whereas a powerful descriptive tool, transformation grids must be carefully interpreted, especially regarding regions of the object that do not have landmarks nearly positioned [43,69]. More details and examples are given in the Results section.

***Discriminant Analysis***

DA is mathematically related to PCA. It finds the axes that optimize between-group differences relative to within group variation. It can be used as a classification tool [43]. It is here used for testing treatments by using tests for sample mean differences including an estimate of the accuracy of shape in predicting groups. The capability of DA to correctly assign specimens to treatments was assessed along the experiment using the averaged datasets for each DPI. In MorphoJ, Discriminant Function Analysis was requested selecting “Treatment” as the classification criterion. By default, DA in MorphoJ performs a parametric Hotelling’s T-square test (multivariate equivalent of the Student´s t-test). Here requested permutations tests were also

performed for the Procrustes and Mahalanobis distances with 1000 random runs. Procrustes and Mahalanobis distances show how far shapes from one group are from the mean of the other group.

**Allometric patterns and size correction**

The covariation between a size variable and shape variables is called allometry. Isometry, by contrast, is the condition where size and shape are independent of each other and usually serves as the null hypothesis. These concepts are rooted in the Gould-Mosimann school of allometry that conceptually separates size and shape [70]. Although size had been removed from forms after GPA, thus leaving shape differences free of it, a consistent trend in change of shape with size may be possible. Allometry can be statistically tested by tests of multivariate regression.

When groups are present, a single regression line through all groups cannot be fit to test allometry because lines could have group-specific slopes or intercepts [35]. To test whether an allometric component is present in each group, separate regressions were performed for each treatment and DPI with Procrustes Coordinates and ln(CS) as dependent and independent variables, respectively. Permutation tests were requested with 10.000 runs.

When at least one group has regression slopes different from zero several tests could be done in order to control for size and repeat analyses to assess whether differences in shape are actually the result of size variation only [34,35,43,70]. A MANCOVA (with treatments as groups, shape coordinates as dependent variables and ln(CS) as the independent variable) was run from 3 to 8 DPI. The MANCOVA is first run allowing each group to have its own slope. Next, the regression analysis is run again but this time it fits a multivariate analysis of covariance with the slopes constrained to be the same in each group (i. e., parallel). Although the percentage of variance explained (%SS) for the regression of the first model is always higher than the second one (constrained by the premise to keep parallel the slopes), if differences are small the allometric trajectories could be considered to be parallel. TPSRegr (v.1.41) provides multivariate and permutation tests for the assessment of that difference [46]. Afterwards, the MANCOVA model tests if slopes are parallel but separate or if they are coincident (same Y-intercept), and a common regression slope including individuals from both treatments could be fit. This allows to correct for size and to test, for example, if DAs are improved after removing the "size-effect" [35].

**Phenotypic Trajectory Analyses (PTA) and morphospace occupation patterns**

Whereas the comparison of allometric vectors allows to test whether shape change is altered at definite DPIs during ORMV infection, a more complete view of ontogenetic alterations needs to measure phenotypic evolution across multiple levels. It allows ontogenetic patterns to be characterized as phenotypic trajectories through the morphospace, rather than phenotypic vectors. The method proposed by Adams and Collyer [71] "*may also be used for determining how allometric or ontogenetic growth trajectories differ, or for quantifying*

*patterns in other data that form a time-sequence*" [71]. Briefly, phenotypic trajectories have three attributes: size, direction and shape.

Trajectory size (*MD*) quantifies the path length of the phenotypic trajectory expressed by a particular group across levels. This represents the magnitude of phenotypic change displayed by that group. If trajectories of two or more groups compared over comparable time periods differ in trajectory size then it indicates differences in rates of morphological change.

Trajectory direction ($\theta$) is a multivariate angle that describes the general orientation of phenotypic evolution in the multivariate trait space. Statistical comparisons of trajectory direction can be used to provide an assessment of patterns of convergence, divergence, and parallelism.

Trajectory shape ($D_{Shape}$) describes the shape of the path of phenotypic evolution through the multivariate trait space. This information is useful because it indicates whether there are differences in how each group occupies the morphospace through the time period.

PTA analysis starts from the PCs for all specimens at all DPIs. They were obtained from the "Combined dataset 3-12 DPI, averaged by ID DPI" of the Supplementary file ORMV.morphoj. The R script developed by Adams and Collyer [71] was run in RStudio [72,73].

However, as has been pointed out by Ciampaglio *et al*. [74], no one method of disparity measurement is sufficient for all purposes. The use ofa combination of techniques should allow a clearer picture of disparity to emerge. With this aim, another available approach to compare shape trajectories through multivariate morphospace was used. Originally developed to study unequal morphological diversification in a clade of South American fishes [75], this approach is useful because allows us to investigate whether a group "explores" different amount of morphospace than others, additionally to possible differences in magnitude of phenotypic evolution. Moreover, density parameters (D) could be calculated to determine whether the amount of morphological change is more or less constrained in the morphospace.

The method was adapted to the present study. As there is not a phylomorphospace and both treatments lack a "common ancestor" but each plant follows its own independent ontogenetic path, nodes and branches do not exist. Rather, each plant possesses its own trajectory without points in common. Therefore, morphological trajectories were calculated for all plants taking these considerations into account. For this purpose, the "Combined dataset 3-12 DPI, averaged by ID DPI" of the Supplementary file ORMV.morphoj was subdivided by ID. Forty new datasets (Mock- and ORMV-inoculated plants from the same previously performed Procrustes fit) were obtained and Procrustes Coordinates and eigenvalues from the seven PCs obtained were exported to an Excel spreadsheet.

**Statistical analyses**

Except otherwise stated, shape analyses were performed using MorphoJ [44] and the TPS series [46], as described in the main text. Paired Hotelling´s tests for intra-treatment inter-DPI shape change analyses and Mann-Whitney tests for rosette growth analysis were executed in PAST [45]. PTAs based on Adams and Collyer [71] were run in R [72]. Excel 2010 was used for Holm´s-Bonferroni sequential test for multiple comparisons [76,77] and hyperellipses calculations using Real Statistics for Excel 2010 (ver. 4.14) [78].

## Results

Morphometrics aims at analyzing the variation and covariation of the size and shape of objects, defining altogether their form. Shape and form might be confusing words, used as synonyms in many languages [13]. Hereafter, it will be followed the GM definition of shape in the sense of [20] that it is "all the geometric information that remains when location, scale and rotational effects are filtered out from an object".

**Landmark configuration, Procrustes fit and outliers detection**

Figure 1A shows an 11-landmark configuration for the Arabidopsis rosette. Plants were inoculated in their third true leaf (24 plants were mock-inoculated and 17 were ORMV-infected) and images were acquired starting from three days post-inoculation (DPI) to 12 DPI (see Materials and Methods).

After executing a full Procrustes fit of each dataset, they were inspected for the presence of outliers. The shape of one Mock-inoculated plant (M2) diverted the most from the rest in 11 out of 16 datasets and was excluded from all datasets for successive analyses. Thus, 23 Mock-inoculated and 17 ORMV-infected plants were used for the subsequent morphometric analyses.

Afterwards, datasets were combined and the "Combined dataset 3-12 DPI" was created with 640 observations included following a common GPA. Then, a wireframe was created that connects consecutive landmarks. This tool aids visualization, as will be explained later. Next, the "Combined dataset 3-12 DPI" was subdivided by DPI. This creates one dataset for each DPI; each one with the two digitization outputs for each plant.

**Assessment of the tangent (Euclidean) space approximation**

By using the combined datasets for Mock-inoculated, ORMV-infected and ALL plants (see Materials and Methods), TPSSmall (v.1.33) was run to compare statistics for distance to reference shape both in Tangent (Euclidean) and Procrustes (Kendall´s) shape space for both treatments separately and for all plants together (Supplementary Table 1). The results showed that maximum Procrustes distances from mean (reference) shape were 0.371 (ORMV), 0.405 (Mock) and 0.400 (ALL). Mean Procrustes distances from mean (reference) shape were 0.168 (ORMV), 0.186 (Mock) and 0.184 (ALL). This indicates a closer arrangement of ORMV shapes in shape space relative to Mock-inoculated plants. Tangent and Procrustes distances were highly similar (Supplementary Table 1) and regressions through the origin for distance in tangent space, Y, regressed onto Procrustes distance, X, showed slopes > 0.98 and correlations > 0.9999 for all groups (Supplementary Table 1

and Supplementary Figure 1). These results are in line with several similar analysis performed onto a variety of biological forms [35,79–81]. Thus, the projections of shapes in Kendall´s shape space onto a tangent Euclidean shape space are good approximations for the studied shapes.

**Testing digitization error and variation between treatments using Procrustes ANOVA**

Eight separate hierarchical (Procrustes) ANOVAs were performed to assess digitization error at each DPI. The analysis was executed simultaneously for both size and shape. Results are shown in Supplementary Table 2. Explained variance (as a % SS) for which individuals accounted was 17.93 to 99.95 for size and 61.00 to 96.74 for shape over all DPIs. By contrast, explained SS (%) for digitization error ranged from 0.01 to 0.12 for size and 0.40 to 1.15 for shape and were almost always two orders of magnitude smaller than individual SS. Thus, digitization error was negligible throughout the digitization process. Furthermore, the results shown in Supplementary Table 2 revealed that for size, the Individual (ID) effect was highly significant at each DPI as evidenced by Goodall´s F-test ($p < 0.0001$). Treatment effect was insignificant from 3 to 5 DPI but starting from 6 DPI the virus affected plant size ($0.0001 < p < 0.03$).

For shape, similar results were obtained. Indeed, the Individual effect was also highly significant at each DPI as evidenced by Goodall´s F-test ($p < 0.0001$) and MANOVA (standing for Multivariate Analysis of Variance) results ($p < 0.0001$). Treatment impacted earlier in shape than size, as differences in shape were evident as soon as 5 DPI ($p = 0.0008$, multivariate test). The infection also had an increasingly proportionally higher impact along the experiment, to reach 82.35 and 38.29 of the explained % SS at the end of the experiment (12 DPI) for size and shape, respectively. Accordingly, ORMV induced a relative growth stagnation that was progressively more accentuated along the experiment (Figure 2).

**Ordination Methods and shape change visualization**

***PCA***

Firstly, PCA was used to assess error measurement (previously quantified by Procrustes ANOVA, Supplementary Table 2). A covariance matrix was created for the "Combined dataset 3-12 DPI" and then a PCA was performed. Scatterplots were generated for the first four PCs, which together account for 87.2 % of total variance (Figure 3). The proximity of equally colored points indicates a small digitization error.

As digitization error explained a negligible percentage of variance, digitizations were averaged within specimens for each DPI. From the "Combined dataset 3-12 DPI" it was created the "Combined dataset 3-12 DPI, averaged by ID DPI" dataset, which contains all 320 averaged observations. The averaged data were used to find the directions of maximal variance between individuals by requesting a PCA. Three types of graphs were obtained: PC shape changes (a diagram showing the shape changes associated with the PCs); Eigenvalues

(histogram showing the percentages of total variance for which the PCs account) and PC scores (scatterplot of PC scores) (Supplementary file ORMV.morphoJ).

PC1 and the first four PCs accounted for 64.2 % and 87.4 % of total variance, respectively. PC Scatterplots show specimen distribution of along the axes of maximum variance (Figure 4A, B). Dots corresponding to early (3-6 DPI) and later (7-12 DPI) stages were colored in a lighter or darker tone, respectively, to aid visualization. The results showed that PC1 is likely an axis related to development (change in shape related with age), because clearly separated early (mostly negative values) from late (positive values) stages of the experiment (Figure 4A). Moreover, at later stages ORMV-infected plants had fewer positive scores in this axis; which suggests that infected plants retained a more juvenile (paedomorphic) shape. Positive extremes of PC2-4 are related to ORMV shapes.

Also, by using the shape coordinates of the 320 averaged observations, it was next investigated whether plants changed their shapes between successive DPIs, within treatments. Intra-treatment paired comparisons of shape are possible using a paired Hotelling´s test (a multivariate analog of the paired t-test). A strong effect of time on shape was evident from the start of the experiment and differences were extremely statistically significant for Mock plants (Supplementary Table 3). From this point, GM visualization tools are used to better understand what these relative positions on scatterplots mean respect to shape differences.

***Visualization of shape changes***

Wireframe graphs (Figure 4C-F) can be requested for each PC of interest from the "PC shape changes" tab by right-clicking onto the displayed image and changing the type of graph. Wireframe graphs connect the landmarks with straight lines for the starting and target shapes by using a previously created Arabidopsis wireframe, thus showing the relative displacements of landmarks from a mean shape. Negative values of PC1 mostly correspond to juvenile (and infected) shapes; positive values of PC1 belong to healthy controls and adults. Hence, by depicting the –PC1 component, target shapes are given negative values (Figure 4C). The –PC1 explains the relative shortening of leaves #11 (the space limited by landmarks 4, 9 and 11) and #12 (landmarks 5, 10 and 11). This makes sense, since younger plants have yet to develop these relatively new leaves. Petioles of leaves #10, #11 and #12 are particularly relatively shortened. Relative to these shortenings, older leaves (#8 and #9) are longer but, interestingly, only its laminae, since its petioles are not relatively elongated. Taken together, PC1 reveals that ORMV impaired the elongation of newer leaves to their normal extent. PC2 (Figure 4D) associates with relative radial displacements of leaves; tips of leaves #8 and #9 (landmarks 1 and 2) come close together, lowering the typical angle between successive leaves from near 137.5° to close to 90°. These relative displacements determine that leaves #9 and #10 form an exaggerated angle of near 180°. PC3 (Figure 4E) is also mostly associated with radial changes in the infected rosette: leaf #10 is relatively displaced towards leaf #8 and the main effect is, again, the increase of the angle between

leaves #9 and #10 to near 180°. PC4 (Figure 4F) explains less proportion of total variance (4.5%) and is mostly related to the relative displacement of the lamina of leaf #11 towards leaf #9 almost without altering its petiole, which functions as a hinge. Leaves #9 and #10 are, as a combination of the effects depicted by PC2 and PC3, both relatively displaced towards leaf #8. Altogether, the wireframe visualization of the first four PCs (which account for more than 87% of total variance) shows that ORMV induces the relative shortening of laminae and (especially) petioles of newest leaves. This shortening is related to a paedomorphic shape. Furthermore, this analysis also demonstrates that ORMV provokes the relative displacement of leaves #9 and #10 towards leaf #8.

Displacement vectors (called ‘‘lollipop graphs’’ in MorphoJ) are arrows drawn between a landmark in a starting shape and the same landmark in a target shape. The dot in the lollipop represents the starting position and the vector is represented by a line departing from it (but in some software the inverse convention is followed, i. e., PAST). Although these visualization are being displaced in the GM literature in favor of more advanced tools [69], here it is presented the case for –PC1, showing the relative displacements of landmarks (Figure 4G). It can be directly compared with Figure 4C.

Finally, Figure 4H-I show exemplified transformation grids for –PC1. Figure 4H depicts the starting (mean) shape, whereas Figure 4I shows the transformed grid for –PC1. The compression of the grid in the central zone is the result of the relative displacement of the space between landmarks 3, 8 and 11 (leaf #10), 4, 9 and 11 (leaf #11) and 5, 10 and 11 (leaf #12) towards the center of the rosette. In addition, grid stretching is detected around landmarks 1 and 2 and reveals the relative expansion of laminae of leaves #8 and #9, since its petioles remain relatively immobile, landmarks 6 and 7). As stated above, visualization with these grids should be cautiously interpreted since the interpolation function deforms the grid between places where no landmark is placed (and no information about even the existence of tissue is guaranteed). Therefore, such visualizations need to be interpreted cautiously in regions that are relatively far from landmarks [69]. To assess these changes in more detail, PCA analyses were performed for each DPI. The “Combined dataset 3-12 DPI, averaged by ID DPI” was subdivided by DPI performing a common Procrustes fit, thus creating eight new datasets (DPIs) (raw data in Supplementary file ORMV.morphoJ). Covariance matrices were generated and a PCA performed for each DPI dataset. PC1 and the first four PCs accounted for 27 to 43% and 78 to 84% of total variance, respectively. PCs beyond PC4 accounted for 5 % or less of variation each. Shape change visualization showed that PC1 gradually separated specimens belonging to different treatments. Mock-inoculated plants were progressively more aligned with positive PC1 values. PC2 was more generally related to ORMV-infected plants in its positive values. Relative shortening of younger leaves and petioles, and relative displacement of leaves towards leaf #8 were progressively more accentuated (Supplementary Figure 2).

***Discriminant Analysis***

So far, distances between individuals were addressed with the aid of PCA. Subsequently a Discriminant Analysis (DA) was performed to test whether differences between treatments are detectable at each DPI (Table 1). At 5 DPI the three tests detected shape differences between treatments ($0.001<p<0.005$). From 6 DPI and beyond, p-values were extremely significant ($p<0.0001$). These results coincide with those obtained by Procrustes ANOVA of shape (Supplementary Table 2). DA maximizes group separation for plotting their differences and predicting group affiliation (classification). The classification of a given specimen (through the discriminant axis) is done using functions that were calculated on samples that included that same specimen (resubstituting rate of assignment). Then, a degree of over-fitting is unavoidable and leads to an overestimate of the effectiveness of the DA. To overcome this problem, one can use a cross-validation or jackknife procedure [35,43]. Jackknife procedure leaves one specimen at a time not used for constructing the Discriminant function and then tests the rate of correct specimen assignment. Only jackknife cross-validated classification tables provide reliable information on groups. Figure 5 displays DA results in group assignment for 3, 7 and 12 DPI and Supplementary Table 4 details these results for all DPIs. As expected, resubstitution rates of assignment (Figure 5A, D, G) were higher than jackknifed counterparts (Figure 5B, E, H) but jackknifed reached high levels of accuracy (≥90%) from 6 DPI and later (Supplementary Table 4). To test whether this level of accuracy was adequate, these results were compared with classification/misclassification tables completed by human observers. The entire image dataset of 7 DPI was given to three expert researchers working with Arabidopsis (one of the authors (S. Asurmendi) and two other researchers from another Institution). They did not know which plants were mock-inoculated or ORMV-infected, except for one Mock-inoculated and one ORMV-infected plant that were given as phenotypic references. These two reference plants were excluded from the dataset for subsequent human classification. The researchers classified the 38 remnant plants (Supplementary Table 4). Human accuracy ranged from 55 to 72.5%, with an average of 64.2%. Therefore, DA outperformed expert human eye by 30% at 7 DPI and yielded higher classification rates from 5 DPI.

Wireframe graphs for 3, 7 and 12 DPI (Figure 5C, F, I) show the difference from Mock to ORMV group. The difference was subtle at 3 DPI, if there were any (Figure 5C), consistently with nonsignificant differences found by DA at this stage. At 7 DPI (Figure 5F), the relative shortening of leaf #11 (landmarks 4, 9 and 11) and the relative increase in the angle between leaves #9 and #10 were evident. These tendencies persisted at 12 DPI (Figure 5I). At this stage, petioles of leaves #11 and #12 were strongly relatively shortened. These results resembled those obtained in Figure 4C-F and approximately summarize shape changes explained by the first four PCs. This indicated that these shape differences not only separated juveniles from adults but also are hallmarks of shape change induced by ORMV. These results are interesting because discriminant axes not necessarily resemble PCA axes [43].

**Allometric patterns and size correction**

As ORMV induced not only changes in shape, but also in size (Supplementary Table 2), it is worth investigating whether shape differences between treatments (within a given DPI) are associated with size differences. In principle, group differences could arise if individuals of one group are different in shape because they grew faster than those from the other group and reached earlier a more advanced developmental stage. Allometry analyses were performed with individual datasets (each corresponding to one separate treatment for each DPI) from the first Digitization (as proven earlier (Figure 3 and Supplementary Table 2), differences between digitization instances were negligible).

Figure 6A and Supplementary Table 5 show groups with statistically significant allometry and predicted SS from regressions (which correspond to allometric variation of shape). Allometry accounted for moderate to high proportion of the total shape variation, since SS reached values of 36% at 6 DPI (Mock). ORMV induced a reduction in the allometric component of shape variation as evidenced by lower predicted SSs throughout the experiment and nonsignificant values of allometry for all except 4 and 5 DPI. For both treatments and particularly for healthy controls, a bell-shaped curve was detected. A maximum of allometry was detected at 6 DPI for Mock plants but a day before for ORMV. Differences between treatments started at 5 DPI, when allometry accounted for 32% and 20% of predicted SS for Mock and ORMV, respectively. This analysis shows that shape variation is much less driven by size heterogeneity (at a given DPI) in ORMV plants. On the other hand, for Mock plants this situation (isometry) occurs at later stages of development (10-12 DPI).

Allometry was detected from 3 to 8 DPI. For this reason, TPSRegr (v. 1.41) was used firstly to determine whether treatment-specific slopes were parallel at each DPI (3 to 8) (Supplementary Table 5). This phenomenon only occurred 3 and 4 DPI ($p>0.05$, non statistically significant slope differences). As slopes were found to be parallel, it is possible to test whether they are separate parallel slopes or coincident (same Y-intercept). TPSRegr tests demonstrated that slopes were coincident ($p>0.05$). Then, size-corrections could only be done for 3 and 4 DPI, since from 5 to 8 DPI slopes were different ($p<0.05$) and groups follow its own allometric pattern and for 10 and 12 DPI there is isometry and size do not correlate with shape variation. Size-correction was done for 3 and 4 DPI separately in MorphoJ, using all 40 plants. Shape variables were regressed onto ln(CS) for each dataset, by pooling regressions within subgroups (treatments) and permutation tests with 10.000 runs were requested. Residuals from the analyses contain the size-free information about shape only and can be used to repeat DAs to test for improved accuracy of discrimination [70]. Group separation was not improved (Figure 6B-E). This is somehow expected since at this stage of the viral infection differences in size or shape are undetectable (Supplementary Table 2, Table 1 and Figure 5). This test and the large overlap between populations in the scatterplot of regression scores on size (Figure 6F, G) suggest that the effect of size ont shape is very similar for both treatments and DPIs. Bigger rosettes have further distal displacements of

leaves #10, 11 and 12 relative to older leaves (#8 and 9) and elongated petioles (Figure 6H, I), thus reflecting the differential internal growth of the rosette. Bigger, more mature rosettes have more developed newest leaves.

**Phenotypic Trajectory Analyses (PTA) and morphospace occupation patterns**

PTA approach (with 1,000 residual randomization permutations) revealed significant differences in the magnitude of phenotypic evolution between the two treatments ($MD_{\text{Mock,ORMV}}$ = 0.100, $P_{size}$ = 0.003). This implies that ORMV-infected plants experienced a lower rate of ontogenetic phenotypic evolution compared to controls. Overall direction of ontogenetic changes were also statistically significantly different ($\theta_{\text{Mock,ORMV}}$ = 18.34°, $P_{\theta}$ = 0.001). Finally, shape assessment analysis showed differences between treatments regarding trajectories over time ($D_{Shape\text{Mock, ORMV}}$ =0.367, $P_{\text{Shape}}$ = 0.001) (Table 2). Accordingly, when phenotypic trajectories were plotted over time through a projection of the first two generated PCs on a plane (Figure 7), it was found that Mock and ORMV plants follow different trajectories across the morphospace. Beyond 6 DPI, ORMV induced a relative stasis along PC1 (major morphological axis).

Regarding differences in morphospace occupation patterns, the morphometric change experienced by a plant throughout ontogeny equals the Euclidean distance (D) between successive points in a morphospace that represents its shape at each DPI. As PCs from a PCA carry all the morphological information extracted from the Procrustes Coordinates, distances are simultaneously calculated over all the PCs by using the Pythagorean Theorem. These distances are designated as morphometric path lengths (ΣD = MPL) (sensu [75]). Mock-inoculated plants traveled on average more distance through morphospace than infected plants ($MPL_{\text{Mock}}$ = 0.6956 vs. $MPL_{\text{ORMV}}$ = 0.5963, p = 0.00025, Mann-Whitney test). Other measures are traditionally used to detect changes in morphospace occupation patterns and the amount of the difference between character states among specimens in morphospace [74], e.g. sum of variances (∑Var). Control plants had higher ∑Var values than infected plants ($\sum Var_{\text{Mock}}$ = 0.0350 vs. $\sum Var_{\text{ORMV}}$ = 0.0230, p = 2.52 x $10^{-6}$, Mann-Whitney test). This result suggested a higher increase in shape change in controls [74]. Morphospace density occupation measures could be obtained taking into account not only MPLs but variances of the PCs across the experiment. If a group folded an equivalent amount of morphometric change into a much smaller region of morphospace than another, then it will have a higher density [75]. Morphometric path density (D) could be calculated as $D_1$ = MPL/∑Var. ORMV-infected plants are more densely restricted in morphospace ($D_{1(\text{Mock})}$ = 20.21 vs. $D_{1(\text{ORMV})}$ = 26.93, p = 2.89 x $10^{-6}$, Mann-Whitney test) (Table 2).

An alternative measure of density ($D_2$ = MPL/V) considers the volume (V) that the group occupies in morphospace. Several volumetric measures are possible [74]. This study considered the volume of a 95% confidence hyperellipse. $D_2$ was obtained by calculating the square root of the product of the eigenvalues of the PCs and comparing them with expected values for a $X^2$ distribution at α = 0.05. Mock-inoculated plants have hyperellipses of higher volume on average ($\text{Hyperellipse}_{(\text{IC95\%})\text{Mock}}$ = 0.0129 vs. $\text{Hyperellipse}_{(\text{IC95\%})\text{ORMV}}$

= 0.0073), although the differences were not statistically significant (p = 0.11888, Mann-Whitney test). Similarly, density measures based on hyperellipses calculations were not statistically significantly different ($D_{2(Mock)}$ = 111.47 vs. $D_{2(ORMV)}$ = 146.34, p = 0.25051, Mann-Whitney test), although ORMV-infected plants had a higher average density. These differences could be because hypervolume calculations can produce extremely small and variable values. The hypervolume is calculated by taking the product of univariate variances and thus any axis or axes with negligible variance will produce a hypervolume value close to zero. Moreover, all multiplied variances are given the same weight and, consequently, PC axes representing a minimal percentage of the total variance could distort conclusions obtained with more informative axes. Thus, hypervolume can be very sensitive to variation in a single character. To avoid this, one must select only the axes with significant variances to represent the disparity among points in morphospace [74]. Therefore, the analysis was repeated including only the first three PCs, which accounted for more than 95% of variance. The results were similar to those previously found for all the parameters, but with significantly different hyperellipse volumes ($Hyperellipse_{(IC95\%)Mock}$ = 0.022 vs. $Hyperellipse_{(IC95\%)ORMV}$ = 0.014, p = 0.0052597, Mann-Whitney test) and $D_2$ parameters ($D_{2(Mock)}$ = 32.97 vs. $D_{2(ORMV)}$ = 41.87, p = 0.040172, Mann-Whitney test) (Table 2).

Altogether, PTA and morphospace occupation patterns showed that Mock-inoculated and ORMV-infected plants follow separate paths through morphospace. They differ in length, direction and shape (Figure 7). They also explore distinct regions of morphospace in a disparate quantity. Control plants experience more diversification of shape, as evidenced by the comparative length of trajectories (*MD* and MPL), have a higher amount of difference between shape states in morphospace (∑Var) throughout the experiment and explore ampler regions of morphospace ($D_1$, $D_2$) (Table 2). Thus, ORMV infection not only alters the direction of ontogenetic shape development, but also diminishes shape change.

**Comparison with TuMV infections**

One goal of applying the GM approach to Arabidopsis studies is to make more objective and repeatable phenotypic comparisons. To this end, the same experimental setup was applied to study viral infections of *A. thaliana* with TuMV, an ssRNA+ virus unrelated to ORMV [82]. The experiment spanned from 4 to 10 DPI. The time point at 12 DPI was discarded because excessive curling of some leaves owing to TuMV infection impaired the correct assignment of landmarks (Supplementary file TuMV 1st.morphoj). Individual datasets were created for each DPI and Procrustes Coordinates were extracted. A combined dataset was created and PCA carried on. After the exclusion of outliers, 27 Mock and 14 TuMV-inoculated plants remained. PCA revealed PC1 accounted for 49.2% of total variance (much less than in the ORMV experiment) and PC1 plus PC2 accounted for 69.3% of total variance. Again, PC1 mostly separates juveniles from adult rosettes and negative values related predominantly to infected plants, which retained a more immature phenotype (Figure

8A). This resultwas supported by the associated wireframe graph, which depicts a relative shortening of leaves #11 and #12, similarly to ORMV-infected plants (Figure 4C). PC2 was strongly positively related to infected plants and, similarly to the ORMV case (Figure 4D), reflected the widening of the angle between leaves #9 and #10. PCs 3 and 4 (Figure 8B-C) accounted for 17.7% of total variance and were mainly negatively related to TuMV infection. Discriminant Analysis (Figure 8D-E) showed that, like with ORMV, group means were statistically significantly different from 5 DPI. Wireframe graphs also evidenced a strong relative shortening of the petioles, like in ORMV infections (Figure 5F, I). This indicates that more compact rosettes are a common outcome of these viral infections. Discriminant power was slightly higher for almost all DPIs in the case of TuMV (Supplementary Table 4, Supplementary Table 6). Moreover, Procrustes Distances were higher for every DPI in the case of TuMV, which induced a Procrustes separation at 8 DPI that only matched at 12 DPI with ORMV-infected plants (Table 1, Supplementary Table 6). These results suggest that TuMV is a more severe virus in Arabidopsis than ORMV, since it induces a more pronounced departure from Mock mean shape. PTA supported this evidence, as evidenced by a subset of 4-10 DPI datasets selected to compare ORMV with TuMV infections (Figure 9A, B, Table 2). Whereas the trajectory size difference in TuMV-infected plants ($MD_{\text{Mock,TuMV}}$) was similar to that obtained in ORMV-infected pants, the multivariate angle ($\theta_{\text{Mock,TuMV}}$) that separates infected (TuMV) from healthy trajectories more than doubled that of the experiment with ORMV. Shape differences ($D_{\textit{Shape}\text{Mock, TuMV}}$) between trajectories almost doubled. Similarly to ORMV infection, most of the other measures indicated a slower rate of shape change compared to Mock plants (Table 2). To visualize and compare shape changes in the ORMV and the TuMV experiments, transformation grids with Jacobian expansion factors and lollipops were performed in PAST for 10 DPI plants (Figure 9C-F). Both viruses induced relative contraction of the rosette around leaf #11 (the most affected) but TuMV induced more severe deformations. To confirm these results and to test reproducibility, we carried out an independent experiment of TuMV infection (Supplementary file TuMV 2nd.morphoj). PTA analyses were ran and trajectory attributes compared (Table 2). The results were similar to those of first TuMV experiment.

Altogether, these results indicated that both TuMV and ORMV induced relative developmental arrest as well as shape change. However, ORMV triggers symptoms that are mainly driven by developmental arrest, whereas TuMV also promotes a higher shape change that impacts more strongly on the overall shape.

## Discussion

Here, several GM tools were applied to assess morphological changes induced by viral infections in Arabidopsis. The GM analysis is a powerful approach owing to its statistical toolbox and its appealing visual analysis of shape change. By conceptually separating size and shape, both factors that determine form could be separately analyzed. Thus, the effect of ORMV infection was detected earlier on shape than in size (Table

1, Supplementary Table 2 and Figure 2). GM analysis greatly outperformed diagnosis when compared against expert human eye (Supplementary Table 4). The effect of time on shape was more pronounced than that of treatment, since the former was detected earlier (Supplementary Table 2 and Supplementary Table 3). This was particularly the case for control rosettes, indicating that normal rosette development is not a scaling up of previous shapes, but a relative displacement of newly developed structures. This process is somewhat impaired by ORMV, which induced the retention of a more juvenile-like phenotype (Figure 4).

Normal allometric growth comprised a lengthening of petioles and laminae of new leaves (#11 and 12) relative to older ones (Figure 6H-I). This process was reversed by ORMV, which also distorted the normal angle of approximately 137.5° between successive leaves. As a result, leaves #9 and 10 were relatively bent towards leaves #8 and 11, which in turn came close together and bent towards the inoculated leaf (#3) that is middle way between them (Figure 5F, I). TuMV provoked similar outcomes (Figure 8) but with an apparent stronger effect, not only regarding the distorted inter-leaves angle, but for the relative contraction of leaf #11 respect to all remaining leaves, including #12 (Figure 8E, Figure 9D, F). It is important to note here that we did not perform absolute individual angle or distances measurements. This kind of traditional morphometric measurements could also be manually taken by using TPSDig2 software and would be an addition to the parameters presented here, where the focus is only on geometric morphometric tools. However, the normal leaf phyllotactic pattern (the arrangement of organs in regular patterns around the stem of a plant) seems not to be changed by these viruses (personal observations, data not shown). Instead, the distortion of the angle determined by the tip of two successive leaves (with its vertex in the center of the plant) appears to arise from the relative outgrowth of the distal part of the lamina. Taking into account the source-to-sink nature of viral movement by phloem [39] and its radial structure [83] it could be hypothesized that virus or viral-induced signals are distributed through the rosette in such a way that they inhibit proximal systemic growth. Future work should test this hypothesis by comparing cell number or size in distal and proximal parts of systemic leaves or by assessing the effect that growth hormones and mutants have in these parameters. This kind of data-based hypothesis is an example of a desirable outcome of the application of GM tools [43] in particular and of phenotyping in general. In this particular study, the observance of the relative shortening of petioles or laminae, and the distorted inter-leaves angles could lead to further experiments to more precisely quantify these discrete phenotypes using also traditional morphometric measurements. These traditional measurements however should be carried on taking into account its intrinsic statistic limitations (see [43], Introduction).

Both viruses diminished shape change, by constraining virus-infected rosettes to smaller regions of multivariate morphospace (Supplementary Table 1, Table 2 and Figures 7 and 9). Ontogeny (the development or course of development of an individual organism) is a genetically-based endogenous process that can be

altered by the environment [84]. Here, both viruses induced the departure of normal ontogenetic development. The consequences of this departure should be further analyzed by measuring relevant traits.

An objective measurement unit of shape change (Procrustes distance) allowed us to compare ORMV- and TuMV- induced shape changes relative to the departure from healthy control shapes (Tables 1 and 2, Supplementary Tables 2 and 6 and Figure 9) and objectively rank symptoms severity. In addition, visualization tools aided us in identifying where to allocate the shape change differences in the rosette (Figure 5C, F, I, Figure 8D-E and Figure 9D, F). In sum, TuMV impacts more strongly on Arabidopsis rosette shape than ORMV.

In this work, two experiments with TuMV were performed to investigate reproducibility. The second experiment was carried out with roughly half the specimens (Table 2). Whereas PTA parameters were very similar between experiments, p-values tend to show a lesser statistical power associated with the smaller sample (Table 2). This point out both to the robustness of the effect found (PTA parameters) and the need to have of a minimum sample size to statistically assess shape differences, an issue that will reasonably be of more concern when studying more subtle effects. Although sampling problems arising from scarcity of specimens is certainly not a problem in Arabidopsis studies, having a large number of plants could indeed pose a problem because of the limited room in expensive growth chambers that are needed to perform physiological experiments in a highly-controlled environment. Minimum sample size estimation is not trivial, because it may vary depending on the natural shape variation within the assessed population and the kind of scientific question being addressed [85]. Further work should investigate the effect of sample size onto statistical parameters, since this parameter affects shape estimates more than size [85].

Trajectory and density parameters could be also used to compare developmental phenotypic plasticity (a term generally used to summarize how a given group responds to different environmental conditions by producing an array of phenotypes [86]). Multivariate reaction norms could be then obtained using shape variables, but also controlling for other variables (size, external factors) and weighting their interaction. This would enrich the description of phenotypes, whereas offering a solid basis for comparisons.

As superior organisms, plants have complex shapes that experience complex changes throughout their life spans, particularly when exposed to severe stresses that modify the route of ongoing development. Thus, their complex phenotypes are difficult to encompass in all their extent by using only one technique, regardless of its descriptive or statistical power. This is important when evaluating the capabilities and limitations of the GM tools presented here. For example, we showed that ORMV significantly impacts rosette shape from and after 5 DPI (Table 1 and Supplementary Table 2). Furthermore, the wireframes (Figure 5C, F, I) helped us to detect that some laminae and (almost all) petioles become relatively shorter under ORMV infection. However, no particular statistical statement could be done about these discrete phenotypic outcomes. Rather, if these

questions were to be specifically addressed, other measures (such as direct measures of petioles´ length) should have been taken. GM analyses performed here pointed to overall shape (and size) changes. Visualization tools could serve as guides to further study the putative underlying mechanisms involved if required. Landmarks analyses come with the limitation of not being capable of extrapolate results to the regions between them without uncertainty. For this reason, the selection of a specific set of landmarks (covering the region of interest) must be well stated at the beginning of the experiment and be sound to study the problem of interest. As with any other technique, caution is needed when interpreting the results because of its limitations. Here, it was investigated the contribution of one type of measurement error, the digitization error, which arises from subjective, human error in landmark placement. Other sources of measurement error have not been investigated here, such as imaging error (corresponding to the camera) and specimen positioning. We limited our measurement error analysis to the error-prone manual placement of landmarks, but the other types of measurement errors are still worth considering. The analysis pipeline is similar and could be performed in MorphoJ or other dedicated software. Moreover, as all biological entities are 3D objects, its approximation to 2D structures inevitably involves some degree of measurement error. This issue has been raised since the first time of Geometric Morphometrics studies [87], but has been somehow neglected until recently [88]. Cardini [88] investigated the two to three dimensional approximation, and found that shape estimates were quite different for highly three-dimensional structures (crania). In our study, we used a particularly flat rosette, generally considered well-suited for 2D approximations [11]. However, with the advancement and lowering costs of 3D imaging and analysis, further studies should benefit from assessing the two to three dimensional approximation over the structures under analysis [88].

After the genomic revolution, there is a need of objective, reproducible, and accurate assessments of plant morphology as a critical missing link to supporting phenomics [89]. In fact, the use of GM tools to analyze plant shape have already started, from a botanical, systematic, archaeological [32,33,35] and even experimental [58] point of view.

The use of GM allows the relativization of deviations from controls in a consistent, objective manner. GPA, which is at the core of this conceptual framework, allows us to compare shapes in Procrustes units of distance. The examples given in this work are necessarily limited, but other applications could be easily envisioned. As the choice of landmarks placement is arbitrary on a given structure, other experimental setups could place them differently to study different stages of growth or other anatomical regions of interest. Importantly, this technique is not a competitor, but a possible complementation of newly developed automated platforms for rosette segmentation. Now possible some platforms can identify the tip of leaves, the center of the rosette and the intersection between lamina and petiole [9,90] and therefore the landmarks used in this study and its

coordinates could be automatically determined. Moreover, the same software used in this work permits GM 3D image analysis, therefore allowing the study of plant species with a more complex architecture.

A hundred years after the revolutionary vision of D'Arcy Thompson's transformation grids and more than 40 years since the beginning of the revolution in morphometrics, GM application for plant phenotyping is starting to develop [34,35,91]. Thus, the research on the plant model species *Arabidopsis thaliana* should benefit from it.

**Availability of supporting data and materials**

Datasets supporting the results of this article are available via the *GigaScience* GigaDB repository [92].


**ACKNOWLEDGEMENTS**

We thank Dr. Flora Sánchez and Dr. Fernando Ponz for the kind gift of ORMV and TuMV virus. We thank Dr. Ken Kobayashi and Nicolás Carlotto for human treatment assignments of plants in the Discriminant Analysis comparison, Dr. Valeria Carreira for critical reading of the manuscript, Dra. Julia Sabio y García for her assistance in English grammar and vocabulary and Mr. Mariano Manacorda for assistance in adapting figure colours to the Color Universal Design for accessibility to colour-blind people. This research was supported by PICT 2014-1163 from Agencia Nacional de Promoción Científica y Tecnológica (ANPCyT) and by project PE 1131022 (INTA). The authors declare that they have no conflict of interests.


**Authors' contributions**

C.A.M. performed the experiments. C.A.M and S.A. designed the experiments, interpreted the data and wrote the manuscript. All authors read and discussed the manuscript.

**Figures and Tables Legends**

**Figure 1. (A)** Landmark configuration in a representative Arabidopsis rosette. An 8 DPI Mock-inoculated rosette is shown. (B) Analysis flowchart showing the different software used in this study, with main features extracted from each one listed below corresponding icon. See main text and Materials and Methods for details.

**Figure 2.** Mean Centroid Size for Mock- and ORMV-inoculated plants across the experiment. Error bars indicate +/- SE. * = $p < 0.05$; ** = $p < 0.01$; *** = $p < 0.0001$, Mann-Whitney tests.

**Figure 3.** Shape variation including all observations and replicas. PCA scatterplots of (A) PC1 vs. PC2 and (B) PC3 vs. PC4. Equally colored dots represent both digitizations of the same specimen, for all DPIs. The scale factor for this graph is directly the magnitude of the shape change as a Procrustes distance in any given direction; the same scaling was used for all axes.

**Figure 4.** Shape variation between specimens (averaged by measurement replicates). PCA scatterplots of (A) PC1 vs. PC2 and (B) PC3 vs. PC4, which together explain 87.4 % of variance. Pale dots = juvenile (3-6 DPI) plants. Dark dots = mature (7-12 DPI) plants. (C-F) Wireframe graphs showing shape changes from the starting (average) shape (bluish green) to the target shape (orange) for the first four PCs. Negative (PC1) and positive (PCs2-4) components are shown, respectively. Here and throughout this work, leaf number is indicated in the wireframe in black. (G) Lollipop graph for the –PC1 component. Lollipops indicate starting position of landmarks with dots. (H-I) Transformation grids for (H) the starting shape and for (I) the target shape (-PC1). Shape changes (C-G and I) are magnified 2X for better visualization.

**Figure 5.** Discriminant analyses of shape variation between treatments at 3 (A- C), 7 (D-F) and 12 (G-I) DPI. Frequencies of discriminant scores obtained by resubstitution rates of assignments (A, D, G) and a jackknife cross-validation (B, E, H) are shown using histogram bars with percentages of correct assignments above each graph. Wireframes comparing mean shapes (C, F, I) are shown magnified 2 times. Mock = bluish green; ORMV = orange.

**Figure 6.** Allometric analyses. (A) Predicted sum of squares from regressions of shape onto ln(CS) for each treatment and DPI. P-values were corrected using Holm´s sequential test ($\alpha$=0.05). * = $p < 0.05$; ** = $p < 0.01$. Allometric analyses for (B, D, F, H) 3 DPI and (C, E, G, I) 4 DPI (Mock = bluish green; ORMV = orange). Cross-validated DAs before (B-C) and after (D-E) size correction with percentages of correct assignments above each graph. (F-G) Scatterplot of regression scores vs. ln (CS). (H-I) Wireframes showing starting mean shape (turquoise) and target shape depicting an increase in one unit of ln(CS) (blue), without magnification.

**Figure 7.** Phenotypic trajectories for Mock and ORMV (3-12 DPI). Scatterplot shows the first two PCs of shape variation across the experiment. Mean values for each DPI are colored and connected with lines. PTA parameters are given (see Materials and Methods). Mock = bluish green; ORMV = orange.

**Figure 8.** Summary of GM analyses for TuMV-infected plants. (A-C) Shape variation between specimens. (A) PCA scatterplot (PC1 vs. PC2). Pale dots = juvenile (4-5 DPI) plants. Dark dots = mature (7-10 DPI) plants. Wireframe graphs from starting (average) shape (bluish green) to target shape (reddish purple) corresponding to –PC1 (to the left) and +PC2 (top) are included. (B-C) Wireframes for–PC3 and –PC4, respectively. (D-E) Frequencies of jackknifed discriminant scores for 7 and 10 DPI respectively, with wireframes depicting shape changes included. Wireframes show starting shape (mock = bluish green) to the target shape (TuMV = reddish purple). Shape change is magnified 2X.

**Figure 9.** Comparison of virus severity. PC plots of PTA for (A) ORMV- and (D) TuMV-infected plants, compared with Mock-inoculated plants (4-10 DPI). PTA parameters are shown (see main text). Transformation grids with lollipops and Jacobian expansion factors were executed in PAST [45] for ORMV- and TuMV-infected plants depicting (mean) shape change from controls to virus-infected plants (B to C and E to F,

respectively) at 10 DPI. Jacobian expansion factors indicate expansions of the grid (yellow to orange red for factors > 1) or contractions (blue for factors between 0 and 1). The same color scale was set for both comparisons. Lollipops indicate target position of landmarks with dots. Leaf #11 (landmarks 4, 9 and 11) is positioned at the bottom.

**Table 1.** Statistical tests for differences between means of treatments at each DPI from DA. Permutation tests with 1000 random runs.

**Table 2.** Comparative trajectory analyses for the full dataset of the ORMV experiment (3-12 DPI), the reduced dataset (4-10 DPI) and the comparisons with TuMV experiments (4-10 DPI).

**Supplementary Figure 1.** Graphical assessment of the Tangent shape space approximation. Scatterplots of distances in the tangent space against Procrustes distances (geodesic distances in radians) for (A) Mock-inoculated plants, (B) ORMV-infected plants and (C) all plants, over all DPIs. A blue line is plotted to show a slope of 1 through the origin. Then a least-squares regression line through the origin is shown in red (for data in which the variation in shape is small this will hide the blue line).

**Supplementary Figure 2.** Wireframes depicting shape change associated with –PC1 values from 3 to 12 DPI (A-H). Green = starting (average) shape; red = target shape. No magnification was applied.

**Supplementary Table 1.** Summary statistics for the comparisons between Tangent (Euclidean) and Procrustes shape distances from average shapes and for regression slopes and correlations between the two distances.

**Supplementary Table 2.** Summary of centroid size and shape variation. Hierarchical sum of squares ANOVA. Main effect: Treatments; random factors: Individuals (ID), Digitization. SS, MS and df refer respectively to sum of squares, mean sum of squares (i.e., SS divided by df) and degrees of freedom. Error1 = digitization error.

**Supplementary Table 3.** Statistical comparisons of intra-treatment shape changes across the ORMV experiment. Holm´s-Bonferroni sequential correction at $\alpha$=0.05.

**Supplementary Table 4.** Classification/misclassification tables from DA for each DPI and human observers for 7 DPI.

**Supplementary Table 5.** Results of allometry tests for each treatment and DPI (top) and of the regression analyses (MANCOVA), for testing differences between slopes when allometry was detected (bottom).

**Supplementary Table 6.** Discriminant Analysis for TuMV. Statistical tests for differences between means of treatments at each DPI from DA (with permutation tests with 1000 random runs) and classification/misclassification tables for each DPI.

**Bibliography:**


1. Granier C, Vile D. Phenotyping and beyond: modelling the relationships between traits. Current Opinion in Plant Biology [Internet]. Elsevier Ltd; 2014;18:96–102. Available from: http://linkinghub.elsevier.com/retrieve/pii/S1369526614000259

2. International Plant Phenotyping Network [Internet]. 2016. Available from: https://www.plant-phenotyping.org/ippn-survey_2016

3. Fahlgren N, Gehan MA, Baxter I. Lights, camera, action: high-throughput plant phenotyping is ready for a close-up. Current Opinion in Plant Biology [Internet]. Elsevier Current Trends; 2015 [cited 2017 Dec 19];24:93–9. Available from: http://www.sciencedirect.com/science/article/pii/S1369526615000266?via%3Dihub

4. Gehan MA, Kellogg EA. High-throughput phenotyping. American journal of botany [Internet]. Botanical Society of America; 2017 [cited 2017 Dec 19];104:505–8. Available from: http://www.ncbi.nlm.nih.gov/pubmed/28400413

5. Vanhaeren H, Gonzalez N, Inzé D. A Journey Through a Leaf: Phenomics Analysis of Leaf Growth in *Arabidopsis thaliana*. The Arabidopsis Book [Internet]. 2015;13:e0181. Available from: http://www.bioone.org/doi/10.1199/tab.0181

6. Dhondt S, Wuyts N, Inzé D. Cell to whole-plant phenotyping: the best is yet to come. Trends in Plant Science [Internet]. 2013 [cited 2017 Apr 27];18:428–39. Available from: http://www.sciencedirect.com/science/article/pii/S1360138513000812

7. De Vylder J, Vandenbussche F, Hu Y, Philips W, Van Der Straeten D. Rosette tracker: an open source image analysis tool for automatic quantification of genotype effects. Plant physiology [Internet]. 2012 [cited 2014 Mar 25];160:1149–59. Available from: http://www.pubmedcentral.nih.gov/articlerender.fcgi?artid=3490612&tool=pmcentrez&rendertype=abstract

8. Green JM, Appel H, Rehrig EM, Harnsomburana J, Chang J-F, Balint-Kurti P, et al. PhenoPhyte: a flexible affordable method to quantify 2D phenotypes from imagery. Plant methods [Internet]. 2012;8:45. Available from: http://www.pubmedcentral.nih.gov/articlerender.fcgi?artid=3546069&tool=pmcentrez&rendertype=abstract

9. Tessmer OL, Jiao Y, Cruz J a, Kramer DM, Chen J. Functional approach to high-throughput plant growth analysis. BMC systems biology [Internet]. BioMed Central Ltd; 2013 [cited 2014 Mar 25];7 Suppl 6:S17. Available from: http://www.ncbi.nlm.nih.gov/pubmed/24565437

10. Camargo A, Papadopoulou D, Spyropoulou Z, Vlachonasios K, Doonan JH, Gay AP. Objective definition of rosette shape variation using a combined computer vision and data mining approach. PLoS ONE. 2014;9.

11. Ispiryan R, Grigoriev I, Castell W, Schäffner AR. A segmentation procedure using colour features applied to images of Arabidopsis thaliana. Functional Plant Biology. 2013;40:1065–75.

12. Krieger JD. Controlling for Curvature in the Quantification of Leaf Form. In: Elewa AMT, editor. Morphometrics for Nonmorphometricians. Springer Berlin Heidelberg; 2010. p. 27–71.

13. Bonhomme V, Picq S, Gaucherel C, Claude J. Momocs: outline analysis using R. Journal of Statistical Software [Internet]. 2013;56:1–24. Available from: http://www.jstatsoft.org/v56/i13/paper%5Cnpapers3://publication/uuid/9FA27917-592B-4216-85D5-BA4A85B8E698

14. Schneider CA, Rasband WS, Eliceiri KW. NIH Image to ImageJ: 25 years of image analysis. Nat Meth [Internet]. Nature Publishing Group, a division of Macmillan Publishers Limited. All Rights Reserved.; 2012;9:671–5. Available from: http://dx.doi.org/10.1038/nmeth.2089

15. Lobet G. Image Analysis in Plant Sciences: Publish Then Perish. Trends in Plant Science [Internet]. 2017 [cited 2017 Jul 12];22:559–66. Available from: http://linkinghub.elsevier.com/retrieve/pii/S1360138517300912

16. Bucksch A, Atta-Boateng A, Azihou AF, Battogtokh D, Baumgartner A, Binder BM, et al. Morphological Plant Modeling: Unleashing Geometric and Topological Potential within the Plant Sciences. Frontiers in Plant Science [Internet]. 2017 [cited 2017 Jul 12];8:900. Available from: http://www.ncbi.nlm.nih.gov/pubmed/28659934

17. Balduzzi M, Binder BM, Bucksch A, Chang C, Hong L, Iyer-Pascuzzi AS, et al. Reshaping Plant Biology: Qualitative and Quantitative Descriptors for Plant Morphology. Frontiers in Plant Science [Internet]. Frontiers; 2017 [cited 2017 Jul 12];08:117. Available from: http://journal.frontiersin.org/article/10.3389/fpls.2017.00117/full

18. Strauss RE. Foreword. In: Elewa AMT, editor. Morphometrics for Nonmorphometricians. Springer Berlin Heidelberg; 2010. p. v–vi.

19. Thompson DW. On Growth and Form. Dover; 1917.

20. Kendall DG. The diffusion of shape. Advances in Applied Probability [Internet]. 1977 [cited 2017 Apr 27];9:428–30. Available from: https://www.cambridge.org/core/product/identifier/S0001867800028743/type/journal_article

21. Kendall DG, Kendall WS. Alignments in two-dimensional random sets of points. Advances in Applied Probability [Internet]. 1980 [cited 2017 Apr 27];12:380–424. Available from: https://www.cambridge.org/core/product/identifier/S0001867800050230/type/journal_article

22. Bookstein FL. Biometrics, biomathematics and the morphometric synthesis. Bulletin of mathematical biology [Internet]. 1996 [cited 2017 Apr 27];58:313–65. Available from: http://www.ncbi.nlm.nih.gov/pubmed/8713662

23. James Rohlf F, Marcus LF. A revolution morphometrics. Trends in Ecology & Evolution [Internet]. 1993 [cited 2017 Apr 27];8:129–32. Available from: http://linkinghub.elsevier.com/retrieve/pii/016953479390024J

24. Adams DC, Rohlf FJ, Slice DE. Geometric morphometrics: Ten years of progress following the 'revolution.' Italian Journal of Zoology. 2004;71:5–16.

25. Cardini A, Loy A. On growth and form in the "computer era": from geometric to biological morphometrics. In: Cardini A, Loy A, editors. Virtual Morphology and Evolutionary Morphometrics in the new millennium. Associazione Teriologica Italiana; 2013. p. 1–5.

26. Slice DE, Bookstein FL, Marcus LF, Rohlf FJ. A Glossary for Geometric Morphometrics [Internet]. 2016. Available from: http://life.bio.sunysb.edu/morph/glossary/gloss1.html

27. Cope J, Corney D, Clark J, Remagnino P, Wilkin P. Plant species identification using digital morphometrics: a review. 2012; Available from: http://dx.doi.org/10.1016/j.eswa.2012.01.073

28. Claude J. Log-shape ratios, Procrustes superimposition, elliptic Fourier analysis: three worked examples in R. In: Cardini A, Loy A, editors. Virtual Morphology and Evolutionary Morphometrics in the new millennium. Associazione Teriologica Italiana; 2013. p. 94–102.

29. Zelditch ML, Sheets HD, Fink WL. Spatiotemporal Reorganization of Growth Rates in the Evolution of Ontogeny. Evolution [Internet]. 2000;54:1363–71. Available from: http://doi.wiley.com/10.1111/j.0014-3820.2000.tb00568.x

30. MacLeod N, Krieger J, Jones KE. Geometric Morphometric Approaches to Acoustic Signal Analysis in Mammalian Biology. In: Cardini A, Loy A, editors. Virtual Morphology and Evolutionary Morphometrics in the new millennium. Associazione Teriologica Italiana; 2013. p. 110–25.

31. Cardini A, Loy A, editors. Virtual Morphology and Evolutionary Morphometrics in the new millenium. [Internet]. the Italian Journal of Mammalogy. Associazione Teriologica Italiana; 2013 [cited 2017 Dec 19]. Available from: http://www.italian-journal-of-mammalogy.it/public/journals/3/issue_241_complete_100.pdf

32. Ros J, Evin A, Bouby L, Ruas M-P. Geometric morphometric analysis of grain shape and the

identification of two-rowed barley (Hordeum vulgare subsp. distichum L.) in southern France. Journal of Archaeological Science [Internet]. Academic Press; 2014 [cited 2017 Dec 19];41:568–75. Available from: http://www.sciencedirect.com/science/article/pii/S0305440313003282

33. Chitwood DH, Rundell SM, Li DY, Woodford QL, Yu TT, Lopez JR, et al. Climate and developmental plasticity: interannual variability in grapevine leaf morphology. Plant Physiology [Internet]. 2016;170:pp.01825.2015. Available from: http://www.plantphysiol.org/content/170/3/1480.abstract?etoc

34. Viscosi V. Geometric morphometrics and leaf phenotypic plasticity: Assessing fluctuating asymmetry and allometry in European white oaks (Quercus). Botanical Journal of the Linnean Society. 2015;179:335–48.

35. Viscosi V, Cardini A. Leaf morphology, taxonomy and geometric morphometrics: A simplified protocol for beginners. PLoS ONE. 2011;6.

36. Scholthof KG, Adkins S, Czosnek H, Palukaitis P, Jacquot E, Hohn T, et al. Top 10 plant viruses in molecular plant pathology. 2011;12:938–54.

37. Matthews REF (Richard EF, Hull R, Matthews REF (Richard EF. Matthews' plant virology. [Internet]. Academic Press; 2002 [cited 2017 Aug 14]. Available from: http://www.sciencedirect.com/science/book/9780123611604

38. Zavallo D, Debat HJ, Conti G, Manacorda CA, Rodriguez MC, Asurmendi S. Differential mRNA accumulation upon early Arabidopsis thaliana infection with ORMV and TMV-Cg is associated with distinct endogenous small RNAs level. PLoS ONE. 2015;10.

39. Manacorda CA, Mansilla C, Debat HJ, Zavallo D, Sánchez F, Ponz F, et al. Salicylic Acid Determines Differential Senescence Produced by Two Turnip mosaic virus Strains Involving Reactive Oxygen Species and Early Transcriptomic Changes. Molecular plant-microbe interactions : MPMI [Internet]. 2013;26:1486–98. Available from: http://www.ncbi.nlm.nih.gov/pubmed/23945002

40. Sánchez F, Manrique P, Mansilla C, Lunello P, Wang X, Rodrigo G, et al. Viral Strain-Specific Differential Alterations in Arabidopsis Developmental Patterns. Molecular Plant-Microbe Interactions. 2015;28:1304–15.

41. Doumayrou J, Leblaye S, Froissart R, Michalakis Y. Reduction of leaf area and symptom severity as proxies of disease-induced plant mortality: the example of the Cauliflower mosaic virus infecting two Brassicaceae hosts. Virus research [Internet]. Elsevier B.V.; 2013 [cited 2014 Mar 21];176:91–100. Available from: http://www.ncbi.nlm.nih.gov/pubmed/23742852

42. Ferrier T, Matus JT, Jin J, Riechmann JL. Arabidopsis paves the way: genomic and network analyses in

crops. Current Opinion in Biotechnology [Internet]. 2011 [cited 2017 Apr 27];22:260–70. Available from: http://www.sciencedirect.com/science/article/pii/S0958166910002284

43. Zelditch ML, Swiderski DL, Sheets HD. Geometric morphometrics for biologists: a primer. London: Academic Press; 2012.

44. Klingenberg CP. MorphoJ: An integrated software package for geometric morphometrics. Molecular Ecology Resources. 2011;11:353–7.

45. Hammer Ø, Harper DAT, Ryan PD. PAST: Paleontological Statistics Software Package for Education and Data Analysis. Palaeontologia Electronica [Internet]. 2001;4. Available from: http://folk.uio.no/ohammer/past

46. Rohlf FJ. The tps series of software. Hystrix, the Italian Journal of Mammalogy. 2015;26:1–4.

47. Aguilar I, Sánchez F, Martin AM, Martinez-herrera D, Ponz F. Nucleotide sequence of Chinese rape mosaic virus (oilseed rape mosaic virus), a crucifer tobamovirus infectious on Arabidopsis thaliana. Plant molecular biology. 1996;191–7.

48. Sánchez F, Martínez-Herrera D, Aguilar I, Ponz F. Infectivity of turnip mosaic potyvirus cDNA clones and transcripts on the systemic host Arabidopsis thaliana and local lesion hosts. Virus research [Internet]. 1998;55:207–19. Available from: http://www.ncbi.nlm.nih.gov/pubmed/9725673

49. Boyes DC, Zayed a M, Ascenzi R, McCaskill a J, Hoffman NE, Davis KR, et al. Growth stage-based phenotypic analysis of Arabidopsis: a model for high throughput functional genomics in plants. The Plant cell [Internet]. 2001;13:1499–510. Available from: http://www.pubmedcentral.nih.gov/articlerender.fcgi?artid=139543&tool=pmcentrez&rendertype=abstract

50. Schutz H, Krieger J. Guide to geometric morphometrics. 2007.

51. Klingenberg C, Peter C. Analyzing Fluctuating Asymmetry with Geometric Morphometrics: Concepts, Methods, and Applications. Symmetry [Internet]. Multidisciplinary Digital Publishing Institute; 2015 [cited 2017 Dec 27];7:843–934. Available from: http://www.mdpi.com/2073-8994/7/2/843/

52. Oxnard C, O'Higgins P. Biology Clearly Needs Morphometrics. Does Morphometrics Need Biology? Biological Theory [Internet]. Springer Netherlands; 2009 [cited 2017 Dec 28];4:84–97. Available from: http://www.mitpressjournals.org/doi/abs/10.1162/biot.2009.4.1.84

53. Nikovics K, Blein T, Peaucelle A, Ishida T, Morin H, Aida M, et al. The balance between the MIR164A and CUC2 genes controls leaf margin serration in Arabidopsis. The Plant cell [Internet]. 2006 [cited 2010 Jul 1];18:2929–45. Available from: http://www.pubmedcentral.nih.gov/articlerender.fcgi?artid=1693934&tool=pmcentrez&rendertype=abstract

54. Bilsborough GD, Runions A, Barkoulas M, Jenkins HW, Hasson A, Galinha C, et al. Model for the regulation of Arabidopsis thaliana leaf margin development. Proceedings of the National Academy of Sciences of the United States of America. 2011;108:3424–9.

55. Biot E, Cortizo M, Burguet J, Kiss A, Oughou M, Maugarny-Calè S A, et al. Multiscale quantification of morphodynamics: MorphoLeaf software for 2D shape analysis. [cited 2017 Oct 12]; Available from: http://dev.biologists.org/content/develop/143/18/3417.full.pdf

56. Carreira VP, Soto IM, Mensch J, Fanara JJ. Genetic basis of wing morphogenesis in Drosophila: sexual dimorphism and non-allometric effects of shape variation. BMC developmental biology [Internet]. BioMed Central; 2011 [cited 2017 May 6];11:32. Available from: http://www.ncbi.nlm.nih.gov/pubmed/21635778

57. Bookstein FL. Morphometric tools for landmark data: Geometry and biology. Cambridge: Cambridge University Press; 1991.

58. Berger BA, Ricigliano VA, Savriama Y, Lim A, Thompson V, Howarth DG. Geometric morphometrics reveals shifts in flower shape symmetry and size following gene knockdown of CYCLOIDEA and ANTHOCYANIDIN SYNTHASE. BMC Plant Biology [Internet]. 2017 [cited 2017 Dec 27];17:205. Available from: https://bmcplantbiol.biomedcentral.com/articles/10.1186/s12870-017-1152-x

59. Savriama Y, Gómez JM, Perfectti F, Klingenberg CP. Geometric morphometrics of corolla shape: dissecting components of symmetric and asymmetric variation in *Erysimum mediohispanicum* (Brassicaceae). New Phytologist [Internet]. 2012 [cited 2017 Dec 27];196:945–54. Available from: http://www.ncbi.nlm.nih.gov/pubmed/22988918

60. Tucić B, Budečević S, Manitašević Jovanović S, Vuleta A, Klingenberg CP. Phenotypic plasticity in response to environmental heterogeneity contributes to fluctuating asymmetry in plants: first empirical evidence. Journal of Evolutionary Biology [Internet]. 2017 [cited 2017 Dec 27]; Available from: http://www.ncbi.nlm.nih.gov/pubmed/29134739

61. Chitwood DH, Headland LR, Ranjan A, Martinez CC, Braybrook SA, Koenig DP, et al. Leaf asymmetry as a developmental constraint imposed by auxin-dependent phyllotactic patterning. The Plant cell [Internet]. American Society of Plant Biologists; 2012 [cited 2017 Dec 27];24:2318–27. Available from: http://www.ncbi.nlm.nih.gov/pubmed/22722959

62. Peaucelle A, Morin H, Traas J, Laufs P. Plants expressing a miR164-resistant CUC2 gene reveal the importance of post-meristematic maintenance of phyllotaxy in Arabidopsis. Development (Cambridge, England) [Internet]. 2007;134:1045–50. Available from: http://www.ncbi.nlm.nih.gov/pubmed/17251269

63. Bookstein FL. Advances in Morphometrics. In: Marcus LF, Corti M, Loy A, Naylor GJ, Slice DE,

editors. Advances in Morphometrics. New York: Plenum Press; 1996. p. 131–151.

64. Shaw RG, Mitchell-Olds T. Anova for Unbalanced Data: An Overview. Ecology [Internet]. WileyEcological Society of America; 1993 [cited 2018 Jan 19];74:1638–45. Available from: http://doi.wiley.com/10.2307/1939922

65. Goodall CR. Procrustes methods in the statistical analysis of shape. J. R. Stat. Soc. B [Internet]. 1991 [cited 2018 Jan 22];53:285–339. Available from: http://www.jstor.org/stable/2345744

66. Klingenberg CP, Barluenga M, Meyer A. SHAPE ANALYSIS OF SYMMETRIC STRUCTURES: QUANTIFYING VARIATION AMONG INDIVIDUALS AND ASYMMETRY. Evolution [Internet]. 2002 [cited 2018 Jan 22];56:1909–20. Available from: http://www.flywings.org.uk/PDF files/Evol2002.pdf

67. Bookstein FL. A method of factor analysis for shape coordinates. American Journal of Physical Anthropology [Internet]. 2017 [cited 2018 Jan 22];164:221–45. Available from: http://doi.wiley.com/10.1002/ajpa.23277

68. Howells WW. Introduction. Multivariate Statistical Methods in Physical Anthropology [Internet]. Dordrecht: Springer Netherlands; 1984 [cited 2018 Jan 22]. p. 1–11. Available from: http://link.springer.com/10.1007/978-94-009-6357-3_1

69. Klingenberg CP. Visualizations in geometric morphometrics: How to read and how to make graphs showing shape changes. Hystrix. 2013;24:15–24.

70. Klingenberg CP. Size, shape and form: concepts of allometry in geometric morphometrics. Development Genes and Evolution [Internet]. Development Genes and Evolution; 2016;1–25. Available from: http://dx.doi.org/10.1007/s00427-016-0539-2

71. Adams DC, Collyer ML. A general framework for the analysis of phenotypic trajectories in evolutionary studies. Evolution. 2009;63:1143–54.

72. RStudio Team. RStudio: Integrated Development for R. [Internet]. Boston, MA: RStudio, Inc.; 2016. Available from: http://www.rstudio.com/

73. RCoreTeam. R: A language and environment for statistical computing [Internet]. Vienna: R Foundation for Statistical Computing; 2016. Available from: https://www.r-project.org/

74. Ciampaglio CN, Kemp M, McShea DW. Detecting changes in morphospace occupation patterns in the fossil record: characterization and analysis of measures of disparity. Paleobiology [Internet]. 2001;27:695–715. Available from: http://www.bioone.org/doi/abs/10.1666/0094-8373%282001%29027%3C0695%3ADCIMOP%3E2.0.CO%3B2

75. Sidlauskas B. Continuous and arrested morphological diversification in sister clades of characiform fishes: A phylomorphospace approach. Evolution. 2008;62:3135–56.

76. Gaetano J. Holm-Bonferroni Sequential Correction: An EXCEL Calculator. 2013.

77. Holm S. A Simple Sequentially Rejective Multiple Test Procedure. Scand J Statist [Internet]. 1979 [cited 2017 May 25];6:65–70. Available from: http://www.jstor.org/stable/4615733

78. Zaiontz C. Real Statistics Resource Pack software [Internet]. 2017. Available from: www.real-statistics.com

79. Dewulf A, De Meulemeester T, Dehon M, Engel M, Michez D. A new interpretation of the bee fossil Melitta willardi Cockerell (Hymenoptera, Melittidae) based on geometric morphometrics of the wing. ZooKeys [Internet]. Pensoft Publishers; 2014 [cited 2017 May 10];389:35–48. Available from: http://zookeys.pensoft.net/articles.php?id=3536

80. Bai M, McCullough E, Song K-Q, Liu W-G, Yang X-K. Evolutionary Constraints in Hind Wing Shape in Chinese Dung Beetles (Coleoptera: Scarabaeinae). Sereno PC, editor. PLoS ONE [Internet]. Elsevier Academic Press; 2011 [cited 2017 May 10];6:e21600. Available from: http://dx.plos.org/10.1371/journal.pone.0021600

81. Parés-Casanova PM, Allés C. No functional sexual dimorphism in Minorcan horse assessed by geometric morphometric methods. Animal Genetic Resources/Ressources génétiques animales/Recursos genéticos animales [Internet]. 2015 [cited 2017 May 10];56:91–5. Available from: http://www.journals.cambridge.org/abstract_S2078633614000514

82. ViralZone [Internet]. Available from: https://viralzone.expasy.org/

83. Taiz L, Zeiger E. Plant physiology. 4th ed. Sinauer Associates Inc., editor. Sunderland; 2006.

84. GOULD SJ. ALLOMETRY AND SIZE IN ONTOGENY AND PHYLOGENY. Biological Reviews [Internet]. Blackwell Publishing Ltd; 1966 [cited 2018 Jan 24];41:587–638. Available from: http://doi.wiley.com/10.1111/j.1469-185X.1966.tb01624.x

85. Cardini A, Seetah K, Barker G. How many specimens do I need? Sampling error in geometric morphometrics: testing the sensitivity of means and variances in simple randomized selection experiments. Zoomorphology [Internet]. Springer Berlin Heidelberg; 2015;134:149–63. Available from: http://dx.doi.org/10.1007/s00435-015-0253-z

86. Pigliucci M. Studying the plasticity of phenotypic integration in a model organism. In: Pigliucci M, Preston K, editors. Phenotypic integration: studying the ecology and evolution of complex phenotypes. New York: Oxford University Press on Demand; 2004. p. 155–75.

87. Roth V. On three-dimensional morphometrics, and on the identification of landmark points. Contributions to Morphometrics. Madrid: Consejo Superior De Investigaciones Científicas; 1993. p. 41–61.

88. Cardini A. Missing the third dimension in geometric morphometrics: how to assess if 2D images really are a good proxy for 3D structures? Hystrix, the Italian Journal of Mammalogy [Internet]. 2014 [cited 2017 Dec 21];25:73–81. Available from: http://www.italian-journal-of-mammalogy.it/article/view/10993/pdf

89. Punyasena SW, Smith SY. Bioinformatic and Biometric Methods in Plant Morphology. Applications in Plant Sciences [Internet]. 2014;2:1400071. Available from: http://www.bioone.org/doi/abs/10.3732/apps.1400071

90. Apelt F, Breuer D, Nikoloski Z, Stitt M, Kragler F. Phytotyping $^{4D}$ : a light-field imaging system for non-invasive and accurate monitoring of spatio-temporal plant growth. The Plant Journal [Internet]. 2015 [cited 2017 Jun 7];82:693–706. Available from: http://www.ncbi.nlm.nih.gov/pubmed/25801304

91. Gómez JM, Torices R, Lorite J, Klingenberg CP, Perfectti F. The role of pollinators in the evolution of corolla shape variation, disparity and integration in a highly diversified plant family with a conserved floral bauplan. Annals of Botany [Internet]. 2016 [cited 2017 May 23];117:889–904. Available from: http://www.ncbi.nlm.nih.gov/pubmed/26884512

92. Manacorda CA, Asurmendi S. Supporting data for "Arabidopsis phenotyping through Geometric Morphometrics". *GigaScience* Database 2018. http://dx.doi.org/10.5524/100457

Click here to access/download;Table;Table 1.xlsx

| **Discriminant Function Analysis** | **3 DPI** | **4 DPI** | **5 DPI** |
|---|---|---|---|
| Difference between means: | | | |
| Procrustes distance: | 0.037 | 0.047 | 0.063 |
| Mahalanobis distance: | 1.799 | 1.924 | 3.815 |
| T-square: | 31.637 | 36.170 | 142.264 |
| *P-value (parametric):* | 0.521 | 0.405 | 0.001 |
| **P-values for permutation tests (1000 permutation runs):** | | | |
| *Procrustes distance:* | 0.549 | 0.182 | 0.005 |
| *T-square (Mahalanobis distance):* | 0.523 | 0.417 | 0.001 |

| 6 DPI | 7 DPI | 8 DPI | 10 DPI | 12 DPI |
|---|---|---|---|---|
| | | | | |
| 0.087 | 0.097 | 0.105 | 0.149 | 0.189 |
| 5.117 | 6.651 | 7.035 | 9.078 | 10.863 |
| 255.916 | 432.438 | 483.790 | 805.573 | 1153.389 |
| <0,0001 | <0,0001 | <0,0001 | <0,0001 | <0,0001 |
| | | | | |
| 0.002 | <0,0001 | <0,0001 | <0,0001 | <0,0001 |
| <0,0001 | <0,0001 | <0,0001 | <0,0001 | <0,0001 |

| | | |
|---|---|---|
| **ORMV3-12 DPI[(a)]** | | |
| | | ***p-value*** |
| $MD_{Mock,ORMV}$ | 0.100 | **0.003** |
| $\vartheta_{Mock,ORMV}$ | 18,34° | **0.001** |
| $D_{ShapeMock,\ ORMV}$ | 0.367 | **0.001** |
| $MPL_{Mock}$ | 0.696 | **2.50E-04** |
| $MPL_{ORMV}$ | 0.596 | |
| $\sum Var_{Mock}$ | 0.035 | **2.52E-06** |
| $\sum Var_{ORMV}$ | 0.023 | |
| $D_{1(Mock)}$ | 20.21 | **2.89E-06** |
| $D_{1(ORMV)}$ | 26.93 | |
| $Hyperellipse_{(IC95\%)Mock}$ | 0.022 | **0,005 *** |
| $Hyperellipse_{(IC95\%)ORMV}$ | 0.014 | |
| $D_{2(Mock)}$ | 32.97 | **0,040 *** |
| $D_{2(ORMV)}$ | 41.87 | |
| | | |
| **ORMV4-10 DPI[(a)]** | | |
| $MD_{Mock,ORMV}$ | 0.085 | **0.005** |
| $\vartheta_{Mock,ORMV}$ | 16,46° | **0.001** |
| $D_{ShapeMock,\ ORMV}$ | 0.343 | **0.037** |
| $MPL_{Mock}$ | 0.472 | **8,03E-04 *** |
| $MPL_{ORMV}$ | 0.401 | |
| $\sum Var_{Mock}$ | 0.022 | **2,21E-05 *** |
| $\sum Var_{ORMV}$ | 0.015 | |
| $D_{1(Mock)}$ | 21.77 | **3,61E-05 *** |
| $D_{1(ORMV)}$ | 28.37 | |
| $Hyperellipse_{(IC95\%)Mock}$ | 0.012 | 0,075 * |
| $Hyperellipse_{(IC95\%)ORMV}$ | 0.009 | |
| $D_{2(Mock)}$ | 48.94 | 0,203 * |
| $D_{2(ORMV)}$ | 56.41 | |
| | | |
| **TuMV4-10 DPI 1st[(b)]** | | |
| $MD_{Mock,TuMV}$ | 0.093 | **0.015** |
| $\vartheta_{Mock,TuMV}$ | 34,41° | **0.001** |
| $D_{ShapeMock,\ TuMV}$ | 0.613 | **0.001** |
| $MPL_{Mock}$ | 0.504 | **0,049 *** |
| $MPL_{TuMV}$ | 0.461 | |
| $\sum Var_{Mock}$ | 0.030 | **0,007 *** |
| $\sum Var_{TuMV}$ | 0.023 | |
| $D_{1(Mock)}$ | 16.94 | **0,017 *** |
| $D_{1(TuMV)}$ | 21.83 | |
| $Hyperellipse_{(IC95\%)Mock}$ | 0.019 | 0,156 * |
| $Hyperellipse(IC95\%)_{TuMV}$ | 0.017 | |

| | | |
|---|---|---|
| $D_{2(Mock)}$ | 32.51 | 0,277 * |
| $D_{2(TuMV)}$ | 46.05 | |
| | | |
| **TuMV4-10 DPI 2nd[c]** | | |
| $MD_{Mock,TuMV}$ | 0.082 | 0.202 |
| $\vartheta_{Mock,TuMV}$ | 35,05° | **0.001** |
| $D_{ShapeMock, TuMV}$ | 0.642 | **0.002** |

*= First 3 PCs considered (>95% total variance)

Units: MD = $D_{Shape}$ = MPL = D1 = D2 = Euclidean distance. θ = degrees. ∑Var = $Hyperellipse_{(CI=95\%)}$ = dime

Statistically significant results in **bold**

[a] N = 23 (Mock) and 17 (ORMV)

[b] N = 27 (Mock) and 14 (TuMV)

[c] N = 14 (Mock) and 8 (TuMV)

nsionless.

Figure 1

Click here to access/download;Fig

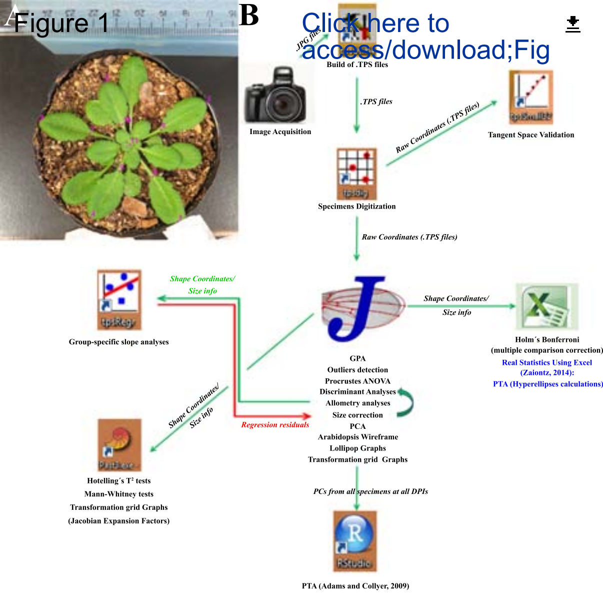

Figure 2

Click here to access/download;Figu

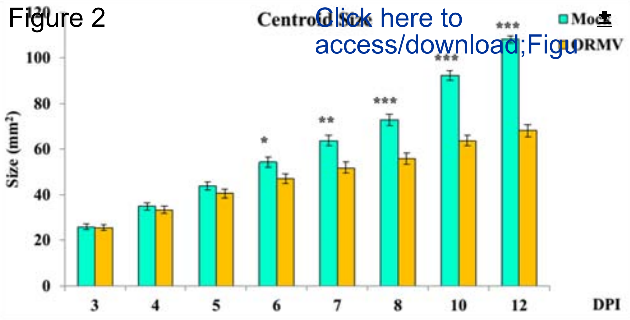

Figure 3

Click here to access/download;Figu

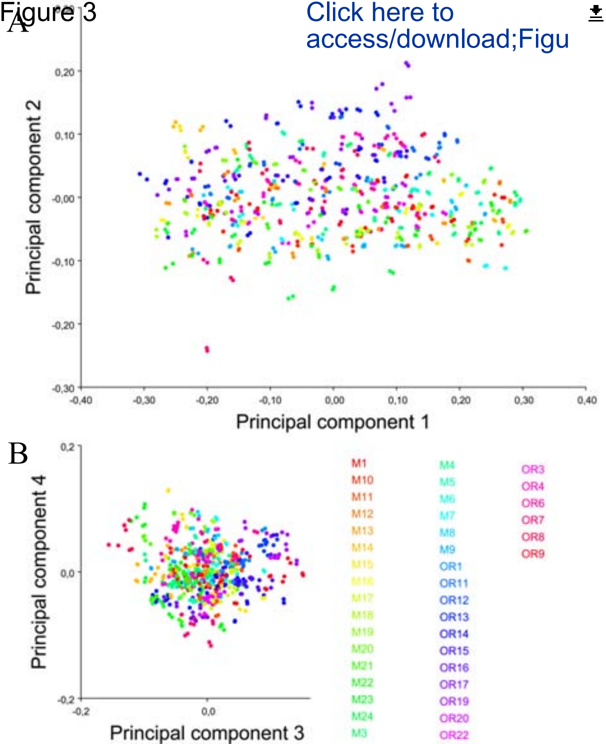

Figure 4

Click here to access/download;Figure;Figure 4.pdf

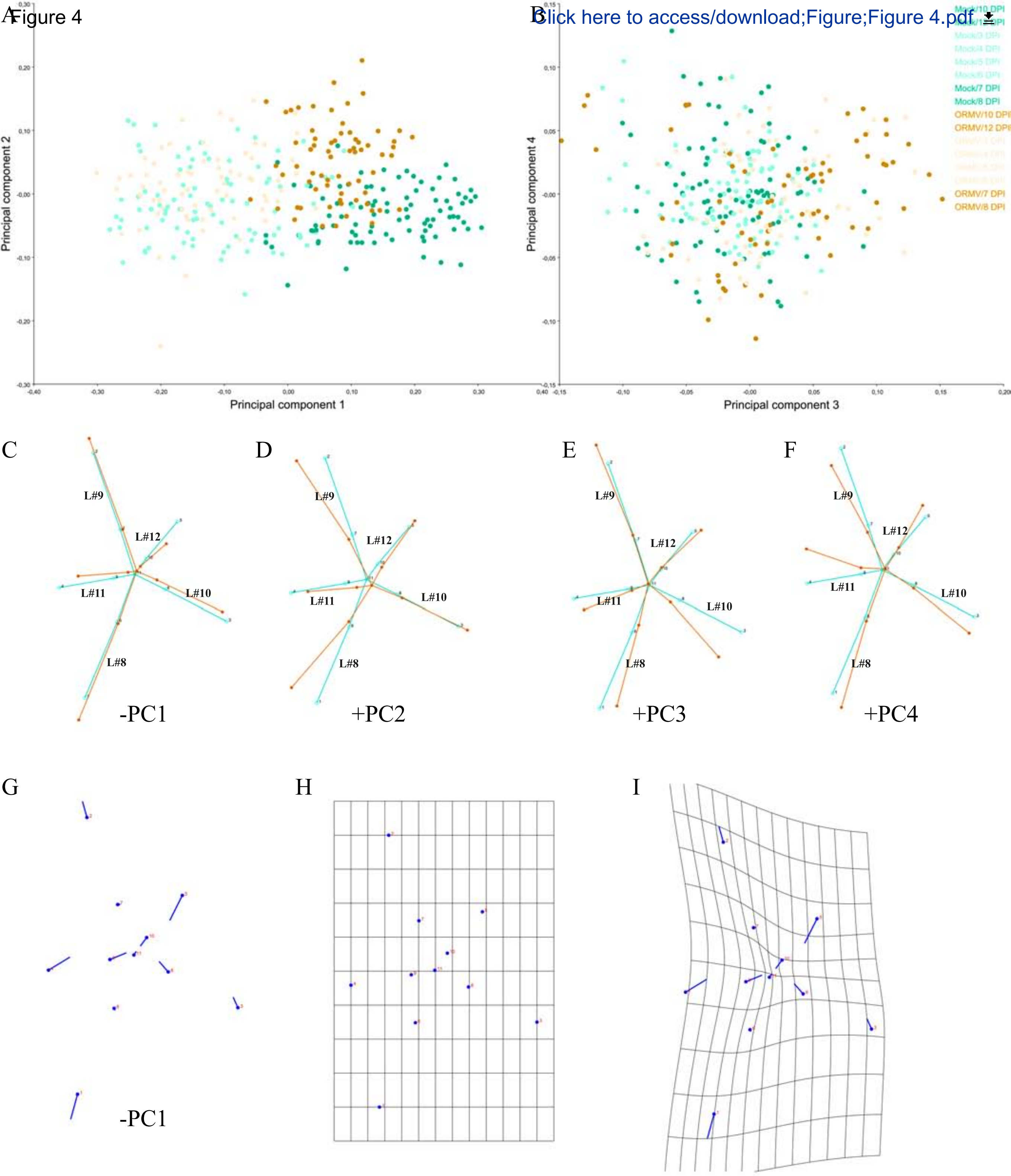

Figure 5

Click here to access/download;Figure;Figure 5.pdf

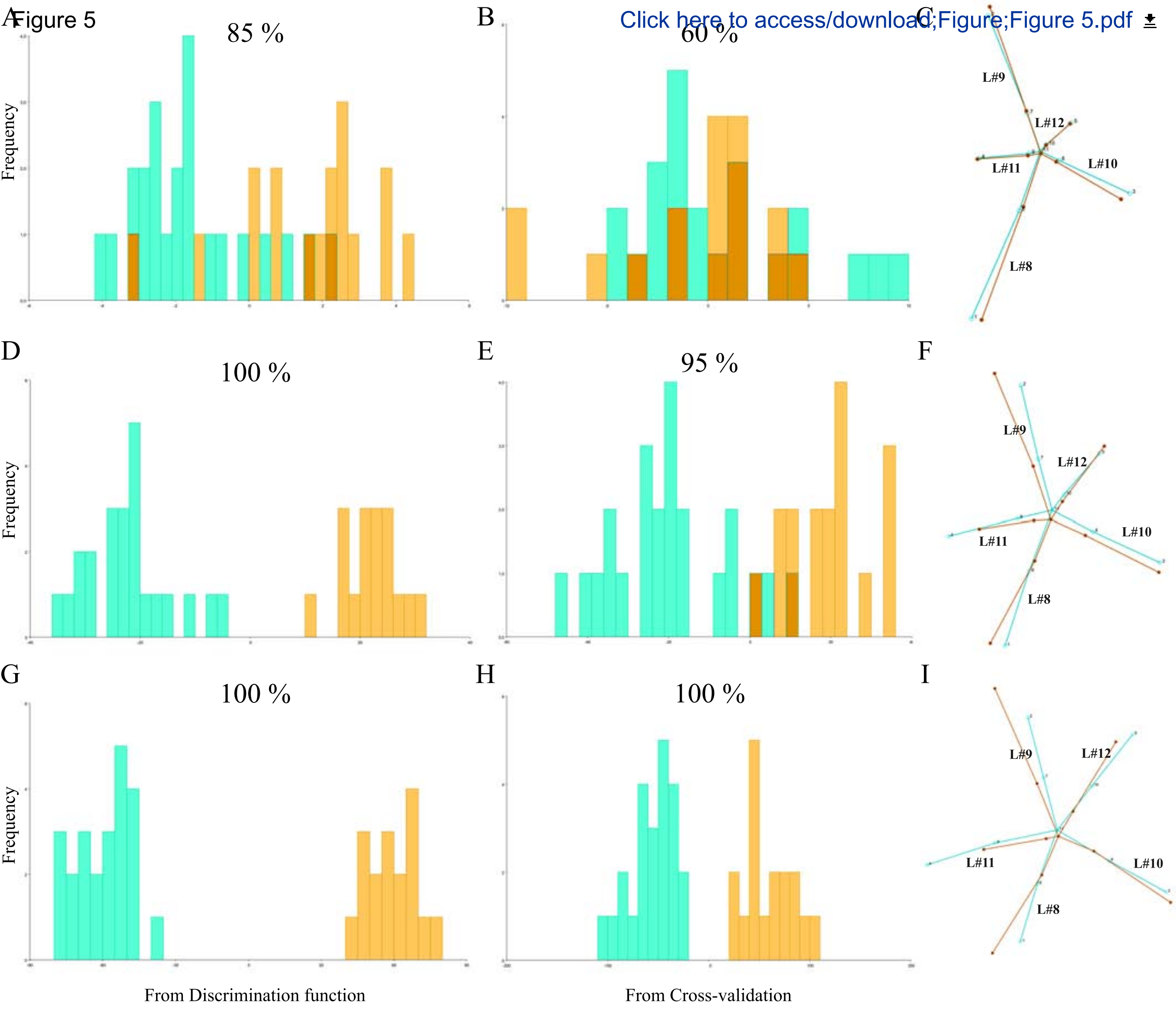

Figure 6

Click here to access/download;Figu

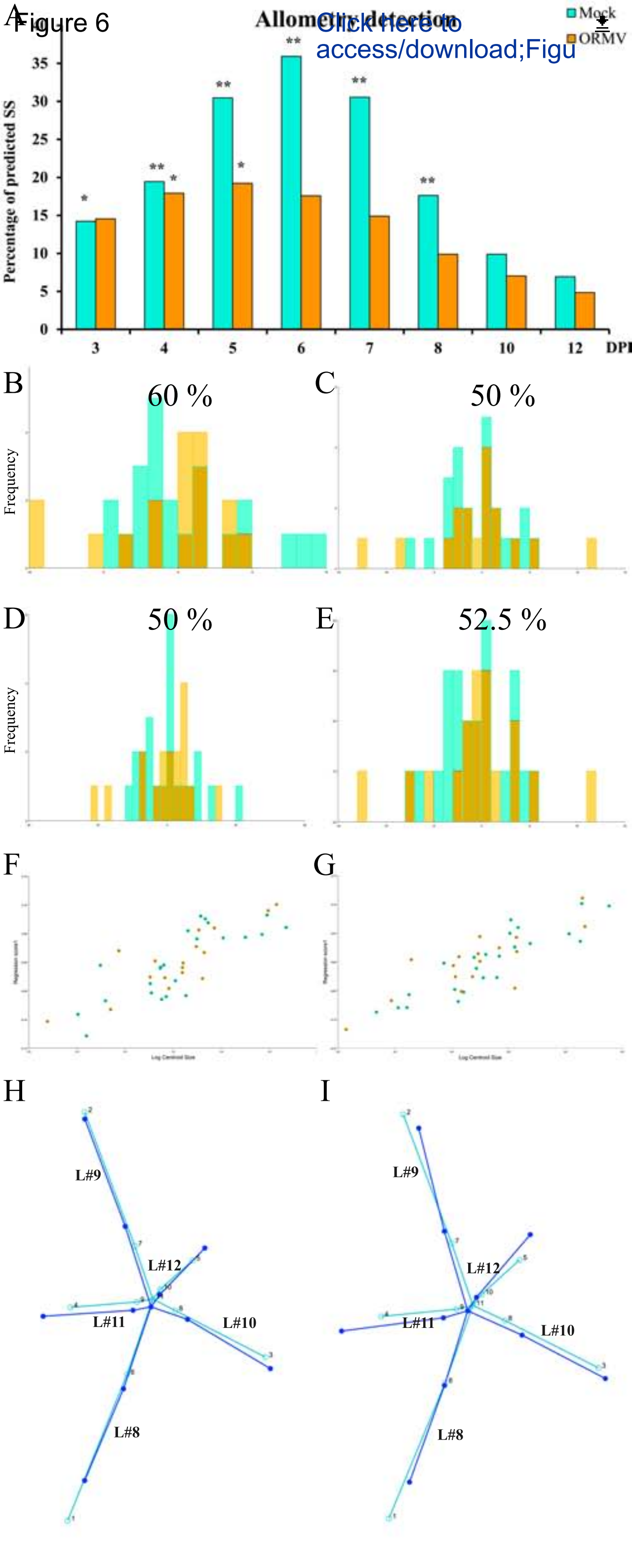

Figure 7

Click here to access/download;Figu

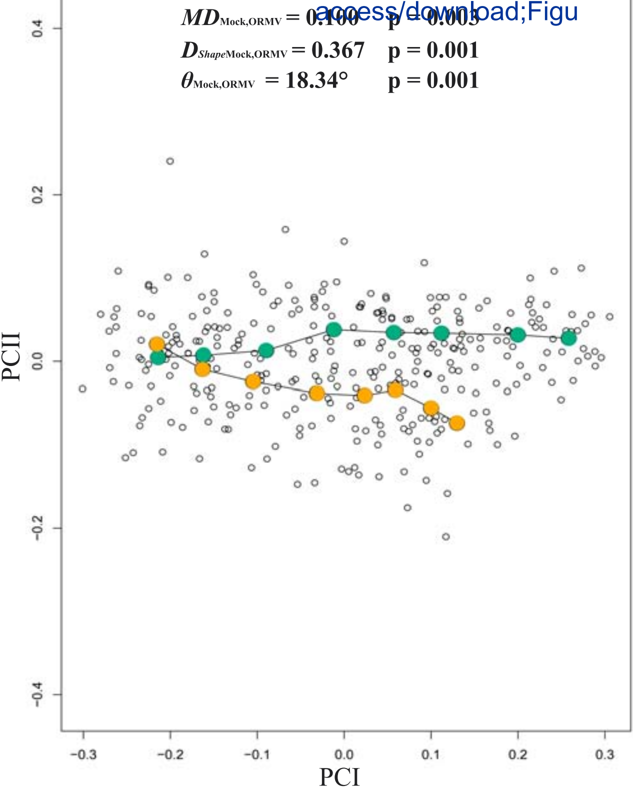

Figure 8

Click here to access/download;Figure;Figure 8.pdf

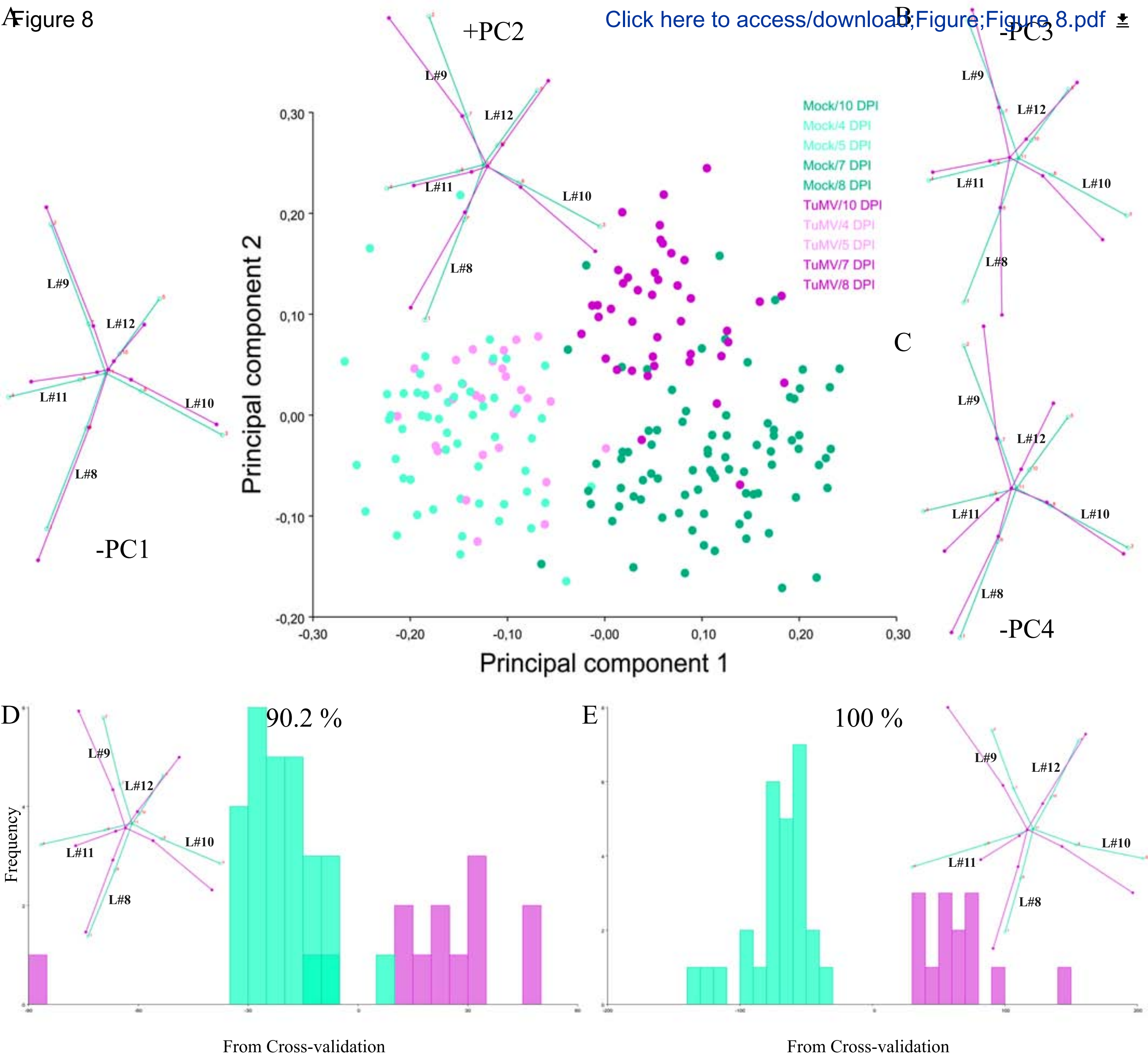

Figure 9

Click here to access/download;Figure;Figure 9.pdf

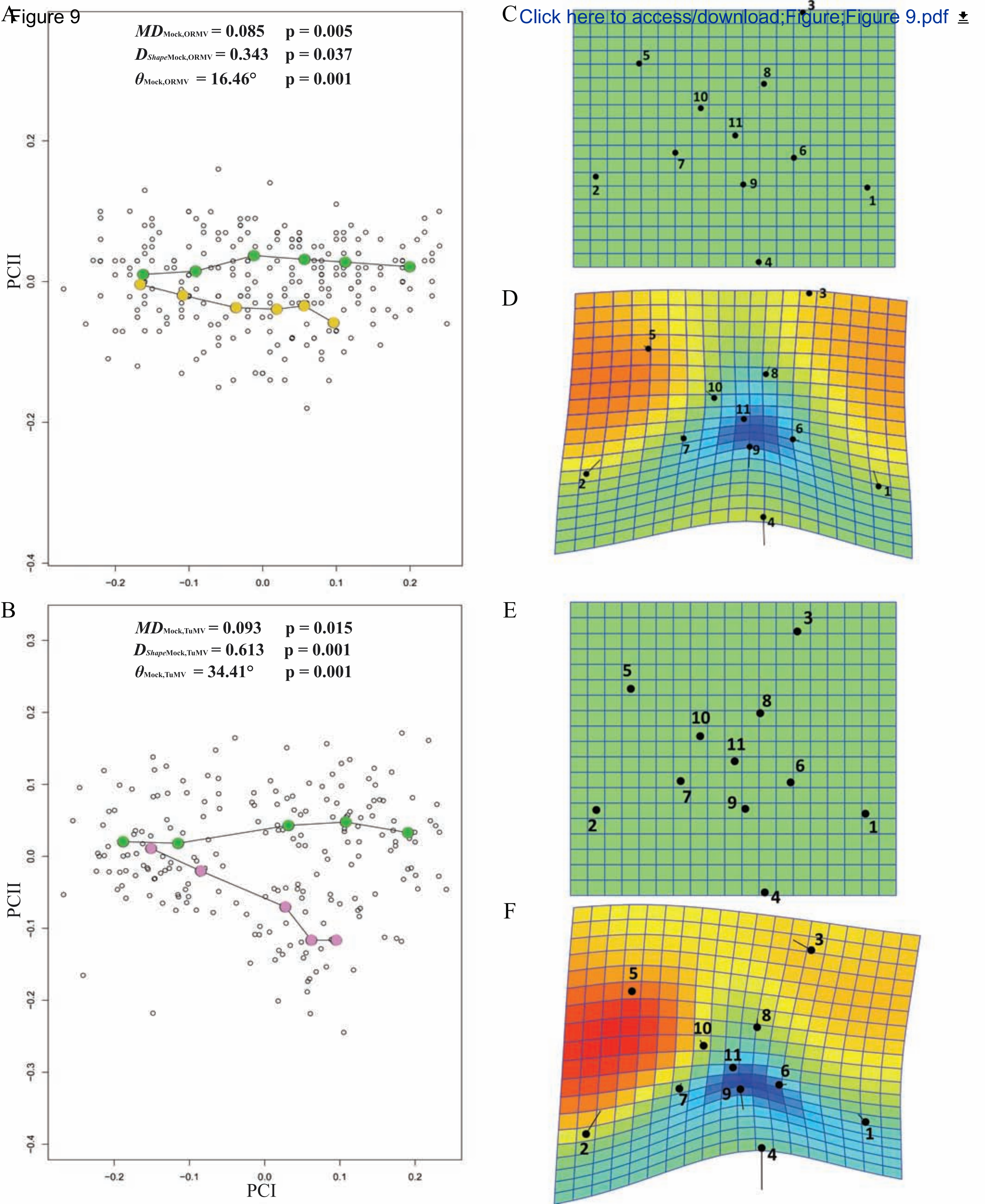

Click here to access/download
**Supplementary Material**
Supplementary Figure 1.jpg

Click here to access/download
**Supplementary Material**
Supplementary Figure 2.pdf

Click here to access/download
**Supplementary Material**
Supplementary Table 1.xlsx

Click here to access/download
**Supplementary Material**
Supplementary Table 2.xlsx

Click here to access/download
**Supplementary Material**
Supplementary Table 3.xlsx

Click here to access/download
**Supplementary Material**
Supplementary Table 4.xlsx

Click here to access/download
**Supplementary Material**
Supplementary Table 5.xlsx

Click here to access/download
**Supplementary Material**
Supplementary Table 6.xlsx

Click here to access/download
**Supplementary Material**
Supplementary file TuMV 2nd

Click here to access/download
**Supplementary Material**
Supplementary file ORMV

Click here to access/download
**Supplementary Material**
Supplementary file TuMV 1st
