# Supplementary figures and images for: Arabidopsis phenotyping through geometric morphometrics
Source: Gigascience. 2018 Jun 18;7(7):giy073. doi: 10.1093/gigascience/giy073 (PMC6041757; doi:10.1093/gigascience/giy073)

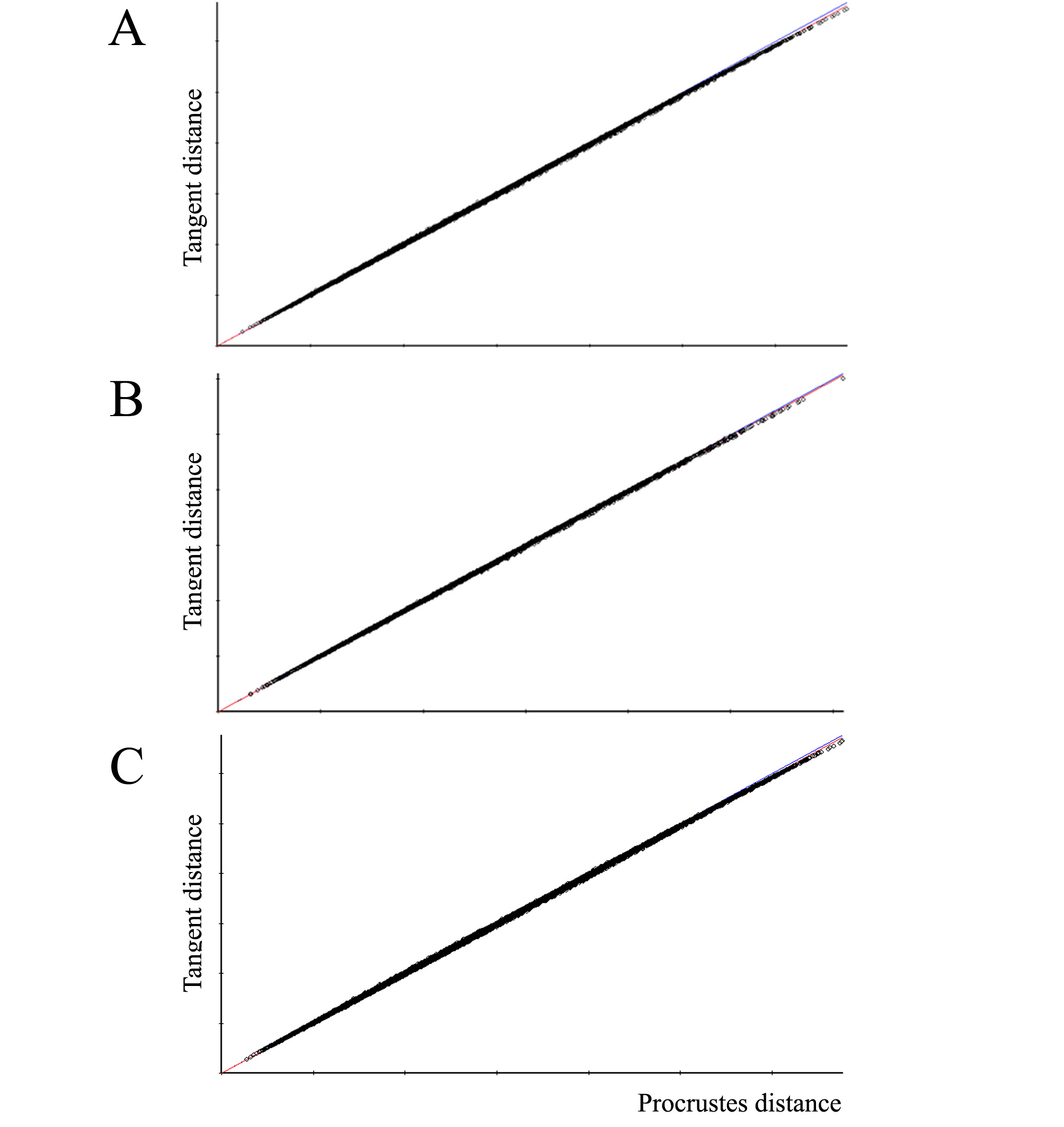

Supplement: Supplement Files [file giy073_supplement_files.zip › Supplementary Figure 1.jpg]

**A**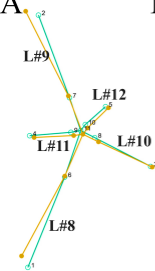**B**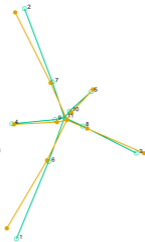**C**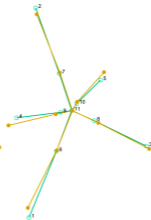**D**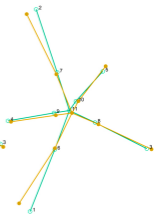**E**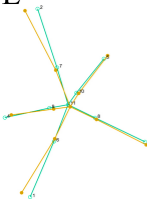**F**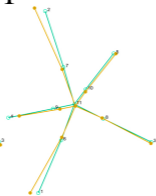**G**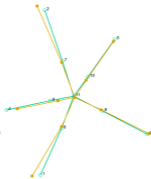**H**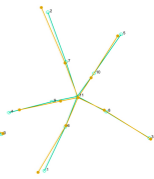

Supplement: Supplement Files [file giy073_supplement_files.zip › Supplementary Figure 2.pdf]
