# Supplementary material for: Arabidopsis phenotyping through geometric morphometrics
Source: Gigascience. 2018 Jun 18;7(7):giy073. doi: 10.1093/gigascience/giy073 (PMC6041757; doi:10.1093/gigascience/giy073)
Supplement: Response_to_Reviewer_Comments_Original_Submission.pdf [file giy073_response_to_reviewer_comments_original_submission.pdf]

Author's Response To Reviewer Comments

Close

Dear Dr. Hans Zauner,

We are pleased to resubmit the paper "Arabidopsis phenotyping through Geometric Morphometrics" after a complete revision of it based on yours and reviewers comments. We have completely reorganized the structure of the Manuscript, by removing entire paragraphs from the Results sections to Methods, to improve readability. Also bearing in mind the easiness of reading, we removed an entire sub-section (former Figure 7) that is not essential to the core of the work, and piles up more methods and information in an already long paper. New citation were included, to deepen explanation in some important topics raised by the Reviewers. Inaccuracies in description of GM tools and/or interpretation of results posed mainly by Dr. Cardini were addressed. Several minor details, English mistakes and some misleading sentences were also fixed. Minor Figures changes suggested by the Reviewers were performed.
As a result, the paper shows essentially same outcome, but many inaccuracies were fixed and the Results were ordered in a way that allows the reader to go through the experiments and main results without having to read all methodological caveats, now condensed in the Methods section for the interested reader.
Below, we answer Reviewers´ comments on the Manuscript point by point. More detailed responses to Dr. Cardini´s pdf sticky notes are found in his original notes file, as individual responses, point by point, in an effort to make it clear where we introduced the requested modifications. The new manuscript version include all the correction mentioned on those responses.
Acknowledging the reviewers for their constructive comments and suggestions, we believe that the manuscript has been considerably improved and we hope it will be of the required quality to be considered for publication in GIGAScience. Please let us know if more modifications needs to be done.

Yours sincerely,

Carlos A. Manacorda and Sebastián Asurmendi

Response to Dr. Savriama:
I would include this reference (Berger et al. 2017) in the paper since this the very first study coupling geometric morphometrics and Virus Induced Gene Silencing (VIGS) to quantify the phenotypic effects of knocking down a single CYC2 paralog, FgCYC2A, as well as the reporter gene, FgANS in symmetry of flowers. Therefore, there is a common experimental and statistical background to both studies that could be briefly mentioned/discussed in this paper.
R: We added the citation suggested by Dr. Savriama in the new version of the Manuscript while discussing symmetry issues and in the Discussion section.
Visualization grid obtained from thin plate splines (TPS) is potentially misleading for figure 9C. I am quoting a passage from Slice's book chapter to address this issue: "It is important that the initial grid cells be square so that deviations from "squareness" can be interpreted as oriented stretching within the cells of the resulting spline plot. This is not a mathematical requirement. It is just harder to assess how a cell has changed in a plot if you are unsure of

its initial shape and distinguishing between initial rectangles and resultant quadrilaterals is more difficult than spotting deviations from squareness." (Slice 2005). It is quite straightforward to re-do this graph using Rohlf's TpsRelw software by simply ticking a box.
R: Regarding the thin-plate-splines of the last Figure, we think Dr. Savriama made a good point indicating the need of the starting shape grid, which is a common practice in many GM works. We modified that Figure in order to now include the starting shapes (Mock-inoculated plants at 10 DPI in each (ORMV or TuMV) experiment), to serve as references against the deformed grids resulting from the superimposition. The grids were also axes-equalized and the exact same scale factor was used for both experiments (very small heatmap scale differences were present in the previous graphs).

Response to Dr. Cardini´s Main Points:
MAIN POINTS
- English is generally good but here and here and there requires improvements.
R: Corrected the vocabulary/gramatical errors detected by Dr. Cardini.
- There's a number of botanical terms and concepts that may require some clarification for the general reader: segmentation, which means completely different things in botany and image analysis, is a good example (but not the only one).
R: Clarified in the new version.
- In general, please, be careful with terminological accuracy. For instance, "point homology" is better being avoided. Even more important: Procrustes shape distances cannot be called "standard units"; they're specific to the landmark configuration being chosen.
R: Clarified in the new version. Procrustes distances are no more called “standard units” in the new version of the Manuscript.

- Besides terms specific to geometric morphometrics and botany, there are several, more general ones, that need to be better defined, as they may not be obvious for all readers (crucial especially in a journal with a broad readership). Just a couple of examples (see more in my pdf comments): "automatons" and high-throughput phenotyping".
R: Clarified in the new version, more bibliography added.
- Minor one: there are too many short paragraphs; quite a few of these can merged.
R: Several paragraphs were merged when appropriate.
- The landmark configuration needs a careful description: the picture in Fig. 1 is of such a low resolution that I cannot make any comment. A botanist with experience on geometric morphometrics would be a better judge on this, especially as the authors suggest that this is an objective and almost standard set of points.
R: The picture in Figure 1A we submitted (as .pdf) is not of low-resolution; we hope the new uploading/final conversion of the figures will allow the reviewer to see the selected landmarks in detail. Besides, we expanded more on the explanation of the selection of landmarks. We proposed this particular set of landmarks based on the purpose of our study (perhaps useful to many others as the stage of development selected here is often chosen to study this organism in the lab). But, whereas it is true that experts in the Arabidopsis field will be the best suited to judge the selection of landmarks, we did not propose that this selection should be universal for Arabidopsis studies (“Here, it is proposed a particular scheme of landmarks placement on Arabidopsis rosette images to study shape variation in the case study of viral infection processes” (Abstract, 17-19), “Also, as pointed out above, landmarks analyses come with the limitation of not being capable of extrapolate results to the regions between them without uncertainty. Because of that, the selection of a specific set of landmarks (covering the region of interest) must be well stated at the beginning of the experiment and be sound to study the problem of interest” (Discussion, 746-749), “…as the

choice of landmarks placement is arbitrary on a given structure, other experimental setups could place them differently to study different stages of growth or other anatomical regions of interest.” (Discussion, 770-772). We also now cited (Oxnard and O´Higgins, 2009) on this issue.
In sum, we tried to not give the impression that our landmark selection should necessarily be standard.
- The paper is presented as largely didactical and aimed at providing guidelines, and I really like this. However, it turns out to be something different (see below) and it's rather unbalanced, as some simple topics are described in details (e.g., that one uses Util to create TPS file and then Dig to load them and digitize landmarks) while others (e.g., 2D approximation, type I-II landmarks etc.) are mentioned without being explained. Finally, a few others purely theoretical issues (e.g., full and partial superimposition) are probably given too much space: in a practical guideline, practical issues should be central and theory explained clearly but briefly and only in relation to the most important analytical sections (thus, again as an example, less on Procrustes - but do provide references - but a bit more on the ANOVA).
R: We modified the manuscript to achieve a more balanced treatment of different topics. We added a paragraph at the end of background section to encourage the reader that require a more practical guidance of the used techniques to read the materials and methods section where most of techniques use, parameters and required consideration are briefly explained. Then the version propose two lanes for the reader -one straight to main data and conclusions and a second more didactical in material and methods without altering the line of main findings exposition with this type of comments.
- Measurement error is said to be assessed but only digitizing error, in fact, seems to be quantified. Again, in a didactical paper, and especially given the relatively complex and 3D structure being studied, it is crucial to be accurate, clear and exhaustive. The mistake of equating measurement and digitizing error is reiterated in the point about repeatability made in the Results subsection on landmarks. There are two ways to address this important shortcoming: 1) redo everything adding at least the assessment of positioning error (but this would be a huge task); 2) leave just the assessment of digitizing error but BE 100% CLEAR THAT THIS IS JUST ONE COMPONENT OF ERROR and say that it would be much better to include all of them.
R: We made it clear that we only measured digitizing error, in the new version. Also, we expanded and added bibliography about the 2D-3D approximation issue.
- p. 6: several issues with how to deal with (and describe) structures with symmetry, as it seems to be the case with the rosettes, given the need of mirror reflection for some specimens. I'd bear in mind that symmetry in plants is often much more complex than in animals: Klingenberg, Savriama et al. have published several important papers on this.
R: We discuss the pattern of symmetry in Arabidopsis rosettes, and cited bibliography regarding this interesting topic (Klingenberg, Savriama…).
- One can provide details on software and methods in a didactical paper, but these should not be in the Results: they'll be in the Methods. All those paragraphs, now largely in the Results, must be moved to the Methods. The paper will have to be reorganized accordingly. See also the comment about lack of balance in details (above).
R: The new version of the Manuscript was fully re-organized, aiming to overcome this problem and to facilitate the reading.
- The subsection describing what landmarks are, besides being inappropriately placed in the Results, is largely inaccurate. Some references on this (cited in several of my papers including, if I am correct, Viscosi & Cardini, 2011, Plos One): O'Higgins (1997 - book chapter on outline methods but full of valid information on why landmark methods were

developed and what landmarks are and are not); Klingenberg (2008); Oxnard & O'Higgins (2009).
R: We revised this subsection, read some additional bibliography and cited Oxnard & O'Higgins (2009) in the new version.
- The methods (again mostly explained in the Results) contain a number of inaccuracies and points which require clarifications (e.g., the ANOVAs).
R: Moved Methods from Results and clarified when pointed out in Dr Cardini´s notes.
- % of variation explained by the effect being tested must be reported for all effects. I'd also add a sentence on the importance of providing estimates of effect size and not just P values (in the Methods or Discussion).
R: "%SS explained" expanded in the new version to allow for comparisons between effects. An entire paragraph addressing the sample size problem is included in the Discussion, also discussing our results regarding that topic.
- The description of the PCA (which is, as many others, inappropriately placed in the Results) can be greatly improved: right now, it is over-simplistic/inaccurate and trivializes some important issues such as the selection of PCs. Besides, there's confusion between statistical analysis and its visualization in geometric morphometrics (read Rohlf's three steps of a GMM study: 1) feature extraction, 2) statistics and 3) visualization; also Bookstein puts it this way in his article definition for an online encyclopedia). Finally, saying that PCs below 5% of variance are biologically irrelevant or that PC1 is a "development-related" axis (and other similar statements) is wrong for the same reasons why, as the authors correctly wrote, PCs are not about group discrimination: PCs only maximize total variance and whether they pick up meaningful axes of variation cannot be said a priori and certainly, even if they align with interesting information, they are suboptimal in capturing that information. For instance, to look for developmental variation, one could more appropriately regress shape onto age.
R: Corrections to fulfil these requirements were done in the new version.
- See Viscosi & Cardini (Fig. 9, in particular) on why describing shape variation in terms of displacement of landmarks is potentially problematic. After suggesting to do this (using superimposed shapes), the authors themselves acknowledge that this is tricky: then, why suggesting it in the first place? Also, it's not tricky because of the relation with the mean shape: it is very difficult and potentially misleading because Procrustes is a statistically convenient but totally arbitrary choice of superimposition (with no biological model behind it).
R: We improved this paragraph and removed a potentially misleading sentence.
- We all agree that one of the strengths of GMM is the visualization. However, the way it is presented in this paper for the rosettes makes interpretations hard for anyone who is not a botanist with experience with that specific configuration of landmarks. For such a complex structure, MorphoJ's outline drawings (with all the necessary caveats) would have been much better.
R: We hope that the visualization of rosettes with the correct image definition will improve the understanding of the landmarks and its connections.
- Up to p. 13 much of the descriptions of shape changes focus on age-related variation and the effect of the virus. To those aims, a clever use of mean shapes (age and/or virus groups) would be much more appropriate and accurate.
R: We agree on that comment and is for this cause that we showed (Suppl Fig 2) the progression of the differences in age groups, to at least partially use mean shapes as recommended.
- The presentation of the DA (in the Results and possibly not even mentioned in the Methods, where it belongs) should start by saying if treatments are compared within age

groups: here too, readers cannot be left to guess until, maybe later, we know what was done. Again, R2s should accompany P values.
R: Clarified in the new version (comparisons were made within age groups).
We could not find any report on R2s on MorphoJ, neither in PAST (v. 3.14) regarding DAs. As this is a DA, not a CVA, there is no more than 1 axis of discrimination.
- Several inaccuracies also in the description (again placed in the Results) of allometry, which, for instance, does not imply linearity. The statistical model is incorrectly described (multivariate regression and not multiple correlation).
R: Corrected in the new version.
- In relation to the regression, on the practical side, it is not true that one cannot use averaged data in TPSRegr, as stated in the paper.
R: Corrected in the new version.
- Too many abbreviations: the number of abbreviations at some point has become so great that I am lost, and probably most readers will be as well.
R: We think that abbreviations are necessary to aid the reading. Moreover, we introduced the meaning of all abbreviations the first time they are used in the new version, following recommendations.
- There should be a table clearly detailing sample sizes of all groups. I may have missed it, however. This table will help having a better idea about issues with unbalanced samples, small samples and power, sample size and the number of variables in multivariate analyses etc.
R: Table 2 now includes that information. Issues regarding unbalanced designs, sample size and statistical power, are discussed in the context of our findings.
- Around p. 17-18, where new topics (disparity etc.) sprung up and bits of Discussion are mixed with Methods (all of them in the Result section), I had to stop the careful review and just gave a very superficial look at the rest of the paper. The story has become so convoluted and, I apologize for saying it this way, chaotic, that there's little point to go on reviewing it very carefully. It cannot be published in the present form and it's very hard even just to assess what's good and what's bad. This is a disservice to the readers and the authors themselves, as one cannot appreciate what's well done and interesting.
R: Though we do not agree with Dr. Cardini in his opinion that the paper is chaotically organized, we do think he has a strong point regarding the manuscript´s length and relative lack of easiness of reading. From the one part, we had tried to show as many approaches for the analyses of the data as we could, partially wanting to show to the Arabidopsis research community that so many GM tools are available, each one suitable for particular research approximations. For such reasons, analyses went from simple size comparisons (new Figure 2) to growth modeling (former Figure 7), to encompass, as wide as possible, several fields of study regarding plant stress. From the other side, however, and from a cold-minded perspective, it is true that such a huge amount of Figures and experiments could discourage readers from completing the reading, thus compromising the very purpose of the paper. Taken all suggestions into account, and further considering that former Figure 7 has a lot of complex information that is not essential to the core of our work, we decided to remove this Figure, along with all its caveats and Discussion, except for size calculations (former Figure 7D, now Figure 2) that is a basic measure often reported in GM studies and a key proxy for infection severity.
Also in the sub-section that is now the last one devoted to ORMV analysis, we changed the title to emphasize the homogeneity of aims between the approaches we used there, for investigating differences in patterns of morphospace occupation.
Several details on how particular analyses were performed were removed from Results section and are now in Methods, as suggested by this reviewer. We hope that the new

structure of the Manuscript will allow a faster assessment of the main findings by looking at a shorter Results section (with one less complex Figure to look at) whilst at the same time permitting the interested reader to search for more experimental and theoretical details in the Methods section.

In conclusion, when I started reading, I thought that the aim of the study was mainly that of exemplifying, in a clear way, how to measure the effect of viruses on rosettes during growth using GMM. That looked interesting and important. However, if this was the aim, it's being swamped by a poor organization (failing to provide a clear outline to the study, mixing sections, having inaccuracies etc.) and by piling up a variety of analyses that sometimes seem to have been added, as the work progressed, without a clear plan. Overall, the result is that this is neither a simple and clear didactical paper nor a well organized research paper. It does have a very good potential but I don't think it is publishable in the current form. Possibly, it should be split in a simplified example study and separately a more complex research paper: in both cases, it has to be organized properly, with rigour, clarity and a clear outline which is consistently followed in all section (each with only the material which is specific to that section).

I am sorry not to be able to be more supportive and I apologize for anything I wrote that may sound harsh. That would be unintentional and simply due to the lack of time to refine and 'nicify' all the comments.

R: Recognizing that the reviewer comments about the difficulty of reading a long paper with many Tables and Figures are right, we have reorganized the structure of the paper, placing a large part of the previous Results section in Methods, when appropriate, to improve the readability of the Manuscript. This way, as mentioned previously, the interested reader could inspect the Methods to deepen on methodological caveats without being forced to do so to go through the main findings.

Many inaccuracies found by Dr. Cardini were fixed at our best knowledge, along with same misleading sentences and English mistakes.

A complete section was removed (former Figure 7) as it does not affect the core results and, whilst appropriate to extract interesting growth and development parameters, it overall contributed to a difficult reading, piling up more and more approaches that could be best suited for a separate publication. We think it will be too contributing to a more friendly reading for both experts and non-experts in GMM.

Close
