# Supplementary material for: Arabidopsis phenotyping through geometric morphometrics
Source: Gigascience. 2018 Jun 18;7(7):giy073. doi: 10.1093/gigascience/giy073 (PMC6041757; doi:10.1093/gigascience/giy073)
Supplement: Response_to_Reviewer_Comments_Revision_1.pdf [file giy073_response_to_reviewer_comments_revision_1.pdf]

Author's Response To Reviewer Comments

Close

Dear Dr. Hans Zauner,

We are pleased to resubmit the paper “Arabidopsis phenotyping through Geometric Morphometrics” revision 2 .
We believe that the manuscript has been considerably improved and we hope it will were able to meet the requirements to be considered for publication in GIGAScience.

Yours sincerely,

Carlos A. Manacorda and Sebastián Asurmendi

Global response

Our aim in this work was to apply GM tools to address the general question of whether differences in rosette shape exist between two experimental conditions applied to Arabidopsis in an experimental context. This purpose aimed be able to statistically quantify the shapes differences between treatments in order to establish objective global comparison on a matter that is usually in some way subjective, as virus phenotype severity for example. Taking individual measurements (angles, petioles and/or laminae lengths, etc.), could indeed give valuable information, but the comparative analysis of shape as a whole needs the application of GM tools, whose mathematical approach allows to simultaneously consider the relative displacements of all selected landmarks taken together. By doing so, a single measure (Procrustes distance) gives the morphometric distance from one shape to the other one (the reference, for example, that could be the mean shape of control plants). Classic morphometric measures have their own problems. For example, it is indeed possible, using the same set of landmarks used here and the same software (TPSDig2), to take measurement distances from the center of the rosette to the end of one petiole, from that point to the tip of the leaf under study and from the center to the tip. However, when several measurements radiate from a single point their values cannot be completely independent; any error in locating that point affects all of these measurements. Such a data set contains less information than could have been collected with no greater effort because some directions are measured redundantly and many measurements overlap (Zelditch, 2012). Similar problems could arise if we had measured successive angles between leaves, as Tim Dickinson properly note (circularity of the data), and this is precisely what we had not done.

Particular responses

Although we do not agree with Dr. Dickinson´s statement that "The authors neglect to demonstrate a problem that their method solves with reference to actual plants." we have done an effort to clarify the understanding of this issue based on the reviewer comments.
We stated in the original manuscript (Page 3, last paragraph) that "This work aims to introduce the use of GM tools for the analysis of Arabidopsis rosette phenotypes in an objective and repeatable way. Viral infections are used as case studies to exemplify the potentially usefulness of these techniques to quantitatively reveal shape changes using this plant model".
We changed to

“This work aims to introduce the use of GM tools for the analysis of the Arabidopsis rosette. Its purpose is to statistically quantify the shape differences between treatments in order to establish objective global comparisons on a matter that is usually subjective, virus phenotype severity. Viral infections are used as case studies to exemplify the potentially usefulness of these techniques to quantitatively reveal shape changes using this plant model. As such, it is not intended to offer a complete introductory explanation of each GM tool, an objective that is beyond the scope of this paper. Such a task was already performed by [33] and for a complete introductory explanation of GM tools applied in biological systems it is recommended the lecture of [41]. Software used in this work frequently has its own user´s manual and informative examples [42–44]. Nevertheless, with the purpose to facilitate the comprehension of this work to newcomers in the field of GM, each tool is briefly described in the Methods section.”

We used actual plants, throughout the study, to demonstrate that viral infections not only affect rosettes´ size (Fig. 2) but also their shape (Figs. 4 to 9 and associated Tables and Suppl. Tables). This effect should not be taken for granted prior to size and shape separation by GM analyses (one cannot exclude a priori a “freezing effect” of the virus that affected size but not shape). Secondly, by performing three independent experiments, we found that the rossette shape is more severely impacted by TuMV than ORMV (Fig. 9A vs. B, D vs. F, and Table 1 vs. Suppl. Table 6). Again, this effect is by no means obvious prior to analyses. The comparison of phenotypic trajectories (PTA, Fig. 9A and B and the associated Table 2) quantified the difference and allowed us to find that, based on trajectory divergence angles and trajectory shape differences TuMV is a more severe virus regarding Arabidopsis rosette shape along the infection time frame. Of course more subtle problems could be addressed using these tools, but this initial work aims to illustrate the capabilities and potential of GM in the field.

Dr. Dickinson stated that “As I understand it, the authors seek to automate the comparison of rosettes so as to analyze the effect of the virus, and perhaps also detect plants in which virus symptoms have appeared”. Whereas he is right in saying that we are interested in the analyses of virus effect and the potential to detect infections by image, regarding automation we only suggested in the Discussion that GM tools could be added to existing software packages (or used to develop newly, dedicated ones), but we performed our landmarks assignation manually here, i. e., without any implementation of automation at this step (we stated it at the M&M section). As mentioned previously the main objective was to establish a quantitative measurement of global shape differences in Arabidopsis that could be automated later on.

Dr. Dickinson follows stating that: “If this really is their objective then I think they need more than the existing Fig. 1 in this ms to demonstrate what they are attempting to do. If the authors' Fig. 1 is revised to meet this concern, then readers can evaluate for themselves whether the authors have succeeded with the great lengths to which they have gone to employ geometric morphometric methods to detect changes in divergence angle between leaves in a rosette, and changes in petiole and blade lengths.”, we have deposited at journal database all images datasets for all three independent experiments at each DPI, along with the .TPS and .morphoJ files that are the input and output of the main analyses and upon which the main conclusion of this work is based on. The interested reader would be fully capable not only to visually inspect every rosette but also to re-run the entire analysis. Fig. 1 only depicts a representative rosette at 8 DPI, with illustrative aims. We modified a sentence in Fig. 1 caption to further clarify this point: Removed: Landmark configuration in an

Arabidopsis rosette. Introduced: Landmark configuration in a representative Arabidopsis rosette. We also slightly modified the sentence introducing Fig. 1 in the main text to make it clearer: (Removed: An 11-landmark configuration for the Arabidopsis rosette is shown in Figure 1A. Introduced : An 11-landmark configuration for a representative Arabidopsis rosette is shown in Figure 1A.).
For the demonstration that our approach here is capable of detecting infected plants, we have done DA analyses (Fig. 5, Suppl. Table 4 including comparison with Human Observers). Again, the interested reader can download the Dataset at every DPI to assign treatments visually and compare her results with those obtained here applying GM tools.
With respect to the last observation made by Dr. Dickinson here about divergence angles and petioles and blade lengths, we want again to emphasize that all differences found are relative to the whole shape change of the structure under consideration here (i.e., the rosette as spatially defined by the landmarks we positioned). We did not state that any structure is more or less lengthy, for example, in absolute terms, but in relative ones (relative to the shape change of the whole configuration). Nonetheless, we think that Dr. Dickinson raises an interesting question here that points out to the possible origin of the (relatively) distorted inter-leaves angles and how to measure them.

Because of that, we introduced a short paragraph in the Discussion to further clarify this issue ("It is important to note here that we did not perform absolute individual angle or distances measurements. This kind of traditional morphometric measurements could also be manually taken by using TPSDig2 software and would be an addition to the parameters presented here, where the focus is only on geometric morphometric tools. However, the normal leaf phyllotactic pattern (the arrangement of organs in regular patterns around the stem of a plant) seems not to be changed by these viruses (personal observations, data not shown). Instead, the distortion of the angle determined by the tip of two successive leaves (with its vertex in the center of the plant) appears to arise from the relative outgrowth of the distal part of the lamina"). Below we added a brief paragraph also stating that "In this particular study, the observance of the relative shortening of petioles or laminae, and the distorted inter-leaves angles could lead to further experiments to more precisely quantify these discrete phenotypes using also traditional morphometric measurements. These traditional measurements however should be carried on taking into account its intrinsic statistic limitations (see [41], Introduction)." We rearranged this section of the Discussion hoping the reader could better understand our point here.

As for the Editor´s comment on the exact meaning of "image segmentation", we clarified further what we meant, trying to aid the reader coming from other fields to fully understand our point here. In the Background section We Removed: "Freely available software that overcome the difficult task of rosette segmentation (an issue still under investigation) have been developed by different means (…)" and Introduced: "Freely available software that overcome the difficult task of image rosette segmentation (i. e. the computational separation of the living plant tissue from the substrate background) is an issue still under investigation and have been developed by different means (…)".

We also had our ms. revised and modified by a specialized person to improve its language and grammar.

We therefore hope we had clarified the points raised both by the Editor and Reviewer #3. We nonetheless remain open to further suggestions to improve our Manuscript.

Close
