# Supplementary material for: Arabidopsis phenotyping through geometric morphometrics
Source: Gigascience. 2018 Jun 18;7(7):giy073. doi: 10.1093/gigascience/giy073 (PMC6041757; doi:10.1093/gigascience/giy073)
Supplement: Reviewer_1_Report_(Original_Submission) -- Andrea Cardini — 12/06/2017 Reviewed [file giy073_reviewer_1_report_(original_submission).pdf]

Reviewer Report

Title: **Arabidopsis phenotyping through Geometric Morphometrics**

Version: **Original Submission** **Date:** 12/6/2017

Reviewer name: **Andrea Cardini**

Reviewer Comments to Author:

Dear Editor and Authors,
please, find my comments as pdf sticky notes in the pdf file (created and probably better viewed using the free software PDF-Xchange viewer). The main ones are in red/orange. Below, I briefly summarize the main issues. Please, bear in mind that, not being a botanist, I'll mainly focus on methods (mostly geometric morphometrics - GMM) and general issues.

Before starting with the comments, if I may, I'd like to make a suggestion for the editorial staff: having a dozens or so of separate supplementary files may be convenient for the publisher but very unfriendly and time-consuming for the reviewers. With the exception of the data files, it's not hard to put everything in a single high resolution pdf. Talking about resolution, that of the figures in the main pdf was so low to make some of them almost useless (check this yourself, please).
Reviewing a very long paper, such as this one, takes me more than a full day: not having to waste time in downloading and checking a dozen of different files would be of help.

MAIN POINTS
- English is generally good but here and here and there requires improvements.
- There's a number of botanical terms and concepts that may require some clarification for the general reader: segmentation, which means completely different things in botany and image analysis, is a good example (but not the only one).
- In general, please, be careful with terminological accuracy. For instance, "point homology" is better being avoided. Even more important: Procrustes shape distances cannot be called "standard units"; they're specific to the landmark configuration being chosen.
- Besides terms specific to geometric morphometrics and botany, there are several, more general ones, that need to be better defined, as they may not be obvious for all readers (crucial especially in a journal with a broad readership). Just a couple of examples (see more in my pdf comments): "automatons" and high-throughput phenotyping".
- Minor one: there are too many short paragraphs; quite a few of these can merged.
- The landmark configuration needs a careful description: the picture in Fig. 1 is of such a low resolution that I cannot make any comment. A botanist with experience on geometric morphometrics would be a better judge on this, especially as the authors suggest that this is an objective and almost standard set of points.
- The paper is presented as largely didactical and aimed at providing guidelines, and I really like this. However, it turns out to be something different (see below) and it's rather unbalanced, as some simple topics are described in details (e.g., that one uses Util to create TPS file and then Dig to load them and digitize landmarks) while others (e.g., 2D approximation, type I-II landmarks etc.) are mentioned without being explained. Finally, a few others purely theoretical issues (e.g., full and partial superimposition) are probably given too much space: in a practical guideline, practical issues should be central and theory explained clearly but briefly and only in relation to the most important analytical sections (thus, again as an example, less on Procrustes - but do provide references - but a bit more on the ANOVA).
- Measurement error is said to be assessed but only digitizing error, in fact, seems to be quantified. Again, in a didactical paper, and especially given the relatively complex and 3D structure being studied, it is crucial to be accurate, clear and exhaustive. The mistake of equating measurement and digitizing error is reiterated in the point about repeatability made in the Results subsection on landmarks. There are two ways to address

this important shortcoming: 1) redo everything adding at least the assessment of positioning error (but this would be a huge task); 2) leave just the assessment of digitizing error but BE 100% CLEAR THAT THIS IS JUST ONE COMPONENT OF ERROR and say that it would be much better to include all of them.
- p. 6: several issues with how to deal with (and describe) structures with symmetry, as it seems to be the case with the rosettes, given the need of mirror reflection for some specimens. I'd bear in mind that symmetry in plants is often much more complex than in animals: Klingenberg, Savriama et al. have published several important papers on this.
- One can provide details on software and methods in a didactical paper, but these should not be in the Results: they'll be in the Methods. All those paragraphs, now largely in the Results, must be moved to the Methods. The paper will have to be reorganized accordingly. See also the comment about lack of balance in details (above).
- The subsection describing what landmarks are, besides being inappropriately placed in the Results, is largely inaccurate. Some references on this (cited in several of my papers including, if I am correct, Viscosi & Cardini, 2011, Plos One): O'Higgins (1997 - book chapter on outline methods but full of valid information on why landmark methods were developed and what landmarks are and are not); Klingenberg (2008); Oxnard & O'Higgins (2009).
- The methods (again mostly explained in the Results) contain a number of inaccuracies and points which require clarifications (e.g., the ANOVAs).
- % of variation explained by the effect being tested must be reported for all effects. I'd also add a sentence on the importance of providing estimates of effect size and not just P values (in the Methods or Discussion).
- The description of the PCA (which is, as many others, inappropriately placed in the Results) can be greatly improved: right now, it is over-simplistic/inaccurate and trivializes some important issues such as the selection of PCs. Besides, there's confusion between statistical analysis and its visualization in geometric morphometrics (read Rohlf's three steps of a GMM study: 1) feature extraction, 2) statistics and 3) visualization; also Bookstein puts it this way in his article definition for an online encyclopedia). Finally, saying that PCs below 5% of variance are biologically irrelevant or that PC1 is a "development-related" axis (and other similar statements) is wrong for the same reasons why, as the authors correctly wrote, PCs are not about group discrimination: PCs only maximize total variance and whether they pick up meaningful axes of variation cannot be said a priori and certainly, even if they align with interesting information, they are suboptimal in capturing that information. For instance, to look for developmental variation, one could more appropriately regress shape onto age.
- See Viscosi & Cardini (Fig. 9, in particular) on why describing shape variation in terms of displacement of landmarks is potentially problematic. After suggesting to do this (using superimposed shapes), the authors themselves acknowledge that this is tricky: then, why suggesting it in the first place? Also, it's not tricky because of the relation with the mean shape: it is very difficult and potentially misleading because Procrustes is a statistically convenient but totally arbitrary choice of superimposition (with no biological model behind it).
- We all agree that one of the strengths of GMM is the visualization. However, the way it is presented in this paper for the rosettes makes interpretations hard for anyone who is not a botanist with experience with that specific configuration of landmarks. For such a complex structure, MorphoJ's outline drawings (with all the necessary caveats) would have been much better.
- Up to p. 13 much of the descriptions of shape changes focus on age-related variation and the effect of the virus. To those aims, a clever use of mean shapes (age and/or virus groups) would be much more appropriate and accurate.
- The presentation of the DA (in the Results and possibly not even mentioned in the Methods, where it belongs) should start by saying if treatments are compared within age groups: here too, readers cannot be left to guess until, maybe later, we know what was done. Again, R2s should accompany P values.
- Several inaccuracies also in the description (again placed in the Results) of allometry, which, for instance, does not imply linearity. The statistical model is incorrectly described (multivariate regression and not multiple correlation).
- In relation to the regression, on the practical side, it is not true that one cannot use averaged data in TPSRegr, as stated in the paper.
- Too many abbreviations: the number of abbreviations at some point has become so great that I am lost, and probably most readers will be as well.

- There should be a table clearly detailing sample sizes of all groups. I may have missed it, however. This table will help having a better idea about issues with unbalanced samples, small samples and power, sample size and the number of variables in multivariate analyses etc.
- Around p. 17-18, where new topics (disparity etc.) sprung up and bits of Discussion are mixed with Methods (all of them in the Result section), I had to stop the careful review and just gave a very superficial look at the rest of the paper. The story has become so convoluted and, I apologize for saying it this way, chaotic, that there's little point to go on reviewing it very carefully. It cannot be published in the present form and it's very hard even just to assess what's good and what's bad. This is a disservice to the readers and the authors themselves, as one cannot appreciate what's well done and interesting.

In conclusion, when I started reading, I thought that the aim of the study was mainly that of exemplifying, in a clear way, how to measure the effect of viruses on rosettes during growth using GMM. That looked interesting and important. However, if this was the aim, it's being swamped by a poor organization (failing to provide a clear outline to the study, mixing sections, having inaccuracies etc.) and by piling up a variety of analyses that sometimes seem to have been added, as the work progressed, without a clear plan. Overall, the result is that this is neither a simple and clear didactical paper nor a well organized research paper. It does have a very good potential but I don't think it is publishable in the current form. Possibly, it should be split in a simplified example study and separately a more complex research paper: in both cases, it has to be organized properly, with rigour, clarity and a clear outline which is consistently followed in all section (each with only the material which is specific to that section).
I am sorry not to be able to be more supportive and I apologize for anything I wrote that may sound harsh. That would be unintentional and simply due to the lack of time to refine and 'nicify' all the comments.

Sincerely

A. Cardini (signed review)

Methods

Are the methods appropriate to the aims of the study, are they well described, and are necessary controls included? No

Conclusions

Are the conclusions adequately supported by the data shown? No

Reporting Standards

Does the manuscript adhere to the journal's guidelines on minimum standards of reporting? No

Choose an item.

Statistics

Are you able to assess all statistics in the manuscript, including the appropriateness of statistical tests used? Yes, and I have assessed the statistics in my report.

Quality of Written English

Please indicate the quality of language in the manuscript: Needs some language corrections before being published

Declaration of Competing Interests

Please complete a declaration of competing interests, considering the following questions:

- Have you in the past five years received reimbursements, fees, funding, or salary from an organisation that may in any way gain or lose financially from the publication of this manuscript, either now or in the future?
- Do you hold any stocks or shares in an organisation that may in any way gain or lose financially from the publication of this manuscript, either now or in the future?
- Do you hold or are you currently applying for any patents relating to the content of the manuscript?
- Have you received reimbursements, fees, funding, or salary from an organization that holds or has applied for patents relating to the content of the manuscript?
- Do you have any other financial competing interests?
- Do you have any non-financial competing interests in relation to this paper?

If you can answer no to all of the above, write 'I declare that I have no competing interests' below. If your reply is yes to any, please give details below.

NA

I agree to the open peer review policy of the journal. I understand that my name will be included on my report to the authors and, if the manuscript is accepted for publication, my named report including any attachments I upload will be posted on the website along with the authors' responses. I agree for my report to be made available under an Open Access Creative Commons CC-BY license (http://creativecommons.org/licenses/by/4.0/). I understand that any comments which I do not wish to be included in my named report can be included as confidential comments to the editors, which will not be published.

I agree to the open peer review policy of the journal

To further support our reviewers, we have joined with Publons, where you can gain additional credit to further highlight your hard work (see: https://publons.com/journal/530/gigascience). On publication of this paper, your review will be automatically added to Publons, you can then choose whether or not to claim your Publons credit. I understand this statement.

Yes Choose an item.
