# Supplementary material for: Arabidopsis phenotyping through geometric morphometrics
Source: Gigascience. 2018 Jun 18;7(7):giy073. doi: 10.1093/gigascience/giy073 (PMC6041757; doi:10.1093/gigascience/giy073)
Supplement: Reviewer_2_Report_(Original_Submission) -- Yoland Savriama — 12/13/2017 Reviewed [file giy073_reviewer_2_report_(original_submission).pdf]

**Reviewer Report**

**Title:** **Arabidopsis phenotyping through Geometric Morphometrics**

**Version:** **Original Submission** **Date:** 12/13/2017

**Reviewer name:** **Yoland Savriama**

**Reviewer Comments to Author:**

Reviewer's report
Title: Arabidopsis phenotyping through Geometric Morphometrics
Authors: Carlos A. Manacorda and Sebastian Asurmendi
Date: 13th December 2017

Review:
This work proposes a complete analytical approach for phenotyping leaf variation in Arabidopsis using geometric morphometrics. In general, the manuscript is well written, thorough and straightforward to read. The hypotheses are clearly presented and the methods seem appropriate for the study. The figures are also neat and very clear.

I much appreciated that the analysis of morphological variation via geometric morphometrics was done in great depth and carefully executed. This paper strongly differs from traditional studies that simply use linear measurements and/or discrete characters for phenotyping. Therefore, it has a particular merit and should set an example for future studies in this field.
I would include this reference (Berger et al. 2017) in the paper since this the very first study coupling geometric morphometrics and Virus Induced Gene Silencing (VIGS) to quantify the phenotypic effects of knocking down a single CYC2 paralog, FgCYC2A, as well as the reporter gene, FgANS in symmetry of flowers. Therefore, there is a common experimental and statistical background to both studies that could be briefly mentioned/discussed in this paper.

- Visualization grid obtained from thin plate splines (TPS) is potentially misleading for figure 9C. I am quoting a passage from Slice's book chapter to address this issue: "It is important that the initial grid cells be square so that deviations from "squareness" can be interpreted as oriented stretching within the cells of the resulting spline plot. This is not a mathematical requirement. It is just harder to assess how a cell has changed in a plot if you are unsure of its initial shape and distinguishing between initial rectangles and resultant quadrilaterals is more difficult than spotting deviations from squareness." (Slice 2005). It is quite straightforward to re-do this graph using Rohlf's TpsRelw software by simply ticking a box.

In my opinion, the manuscript is suitable for publication after these minor modifications have been taken into account.

References
Berger, B. A., Ricigliano, V. A., Savriama, Y., Lim, A., Thompson, V., & Howarth, D. G. (2017). Geometric morphometrics reveals shifts in flower shape symmetry and size following gene knockdown of CYCLOIDEA and ANTHOCYANIDIN SYNTHASE. BMC Plant Biology, 17.
Slice D.E., 2005. Modern morphometrics. In: Slice D.E. (Ed.). Modern morphometrics in physical anthropology. Klewer Press, New York, pp. 1-45

**Methods**

Are the methods appropriate to the aims of the study, are they well described, and are necessary controls included? Yes

**Conclusions**

Are the conclusions adequately supported by the data shown? Yes

**Reporting Standards**

Does the manuscript adhere to the journal's guidelines on minimum standards of reporting? Yes

Choose an item.

**Statistics**

Are you able to assess all statistics in the manuscript, including the appropriateness of statistical tests used? Yes, and I have assessed the statistics in my report.

**Quality of Written English**

Please indicate the quality of language in the manuscript: Acceptable

**Declaration of Competing Interests**

Please complete a declaration of competing interests, considering the following questions:

- Have you in the past five years received reimbursements, fees, funding, or salary from an organisation that may in any way gain or lose financially from the publication of this manuscript, either now or in the future?
- Do you hold any stocks or shares in an organisation that may in any way gain or lose financially from the publication of this manuscript, either now or in the future?
- Do you hold or are you currently applying for any patents relating to the content of the manuscript?
- Have you received reimbursements, fees, funding, or salary from an organization that holds or has applied for patents relating to the content of the manuscript?
- Do you have any other financial competing interests?
- Do you have any non-financial competing interests in relation to this paper?

If you can answer no to all of the above, write 'I declare that I have no competing interests' below. If your reply is yes to any, please give details below.

I declare that I have no competing interests

I agree to the open peer review policy of the journal. I understand that my name will be included on my report to the authors and, if the manuscript is accepted for publication, my named report including any attachments I upload will be posted on the website along with the authors' responses. I agree for my report to be made available under an Open Access Creative Commons CC-BY license

(http://creativecommons.org/licenses/by/4.0/). I understand that any comments which I do not wish to be included in my named report can be included as confidential comments to the editors, which will not be published.

I agree to the open peer review policy of the journal

To further support our reviewers, we have joined with Publons, where you can gain additional credit to further highlight your hard work (see: https://publons.com/journal/530/gigascience). On publication of this paper, your review will be automatically added to Publons, you can then choose whether or not to claim your Publons credit. I understand this statement.

Yes Choose an item.
