# Supplementary material for: Arabidopsis phenotyping through geometric morphometrics
Source: Gigascience. 2018 Jun 18;7(7):giy073. doi: 10.1093/gigascience/giy073 (PMC6041757; doi:10.1093/gigascience/giy073)
Supplement: Reviewer_3_Report_(Revision_1) -- Tim Dickinson — 03/26/2018 Reviewed [file giy073_reviewer_3_report_(revision_1).pdf]

Reviewer Report

Title: **Arabidopsis phenotyping through Geometric Morphometrics**

Version: **Revision 1** **Date:** 3/26/2018

Reviewer name: **Tim Dickinson**

Reviewer Comments to Author:

What I see in this ms is something neither reviewer spoke to. The authors neglect to demonstrate a problem that their method solves with reference to actual plants. I think one may exist, when for example I look at a paper like Manacorda et al. (2013) Salicylic Acid Determines Differential Senescence Produced by Two Turnip mosaic virus Strains Involving Reactive Oxygen Species and Early Transcriptomic Changes. Molecular plant-microbe interactions 26:1486-1498 (https://apsjournals.apsnet.org/doi/pdfplus/10.1094/MPMI-07-13-0190-R) and its Fig. 1, and see how virus infection affects Arabidopsis rosettes. As I understand it, the authors seek to automate the comparison of rosettes so as to analyze the effect of the virus, and perhaps also detect plants in which virus symptoms have appeared. If this really is their objective then I think they need more than the existing Fig. 1 in this ms to demonstrate what they are attempting to do.

If the authors' Fig. 1 is revised to meet this concern, then readers can evaluate for themselves whether the authors have succeeded with the great lengths to which they have gone to employ geometric morphometric methods to detect changes in divergence angle between leaves in a rosette, and changes in petiole and blade lengths. Such a revision would make it clearer that with divergence angles, the authors are dealing with circular data, and may need to think about the constraints that imposes (see Zar's Handbook of Biological Statistics, chapters 26 and 27). If the effect on the phyllotactic spiral (divergence angles) is decoupled from the effects on individual leaves then there is no necessary reason to employ geometric morphometrics.

As I understand it, GigaScience publishes reviews as well as the paper itself, so the journal can incorporate my comments into their summary of the reviews the ms received, and publish this paper. Some readers will be encouraged to learn about geometric morphometrics and how to use ImageJ and other programs, and any shortcomings of the paper won't matter. More critical readers will benefit from Cardini's review, and evaluate the paper accordingly. My sense of the ms is that the authors are competent users of the methods they advocate, and it's up to readers to decide whether the authors successfully demonstrate their value in this particular application.

Methods

Are the methods appropriate to the aims of the study, are they well described, and are necessary controls included? Yes

Conclusions

Are the conclusions adequately supported by the data shown? Yes

Reporting Standards

Does the manuscript adhere to the journal's guidelines on minimum standards of reporting? Yes

Choose an item.

Statistics

Are you able to assess all statistics in the manuscript, including the appropriateness of statistical tests used? Yes, and I have assessed the statistics in my report.

Quality of Written English

Please indicate the quality of language in the manuscript: Acceptable

Declaration of Competing Interests

Please complete a declaration of competing interests, considering the following questions:

- Have you in the past five years received reimbursements, fees, funding, or salary from an organisation that may in any way gain or lose financially from the publication of this manuscript, either now or in the future?
- Do you hold any stocks or shares in an organisation that may in any way gain or lose financially from the publication of this manuscript, either now or in the future?
- Do you hold or are you currently applying for any patents relating to the content of the manuscript?
- Have you received reimbursements, fees, funding, or salary from an organization that holds or has applied for patents relating to the content of the manuscript?
- Do you have any other financial competing interests?
- Do you have any non-financial competing interests in relation to this paper?

If you can answer no to all of the above, write 'I declare that I have no competing interests' below. If your reply is yes to any, please give details below.

I declare that I have no competing interests

I agree to the open peer review policy of the journal. I understand that my name will be included on my report to the authors and, if the manuscript is accepted for publication, my named report including any attachments I upload will be posted on the website along with the authors' responses. I agree for my report to be made available under an Open Access Creative Commons CC-BY license (http://creativecommons.org/licenses/by/4.0/). I understand that any comments which I do not wish to be included in my named report can be included as confidential comments to the editors, which will not be published.

I agree to the open peer review policy of the journal

To further support our reviewers, we have joined with Publons, where you can gain additional credit to further highlight your hard work (see: https://publons.com/journal/530/gigascience). On publication of this paper, your review will be automatically added to Publons, you can then choose whether or not to claim your Publons credit. I understand this statement.

Yes Choose an item.
